# ASCEND-Eye: Rationale, design and baseline characteristics for a sub-study of the ASCEND randomised trial, exploring the effects of aspirin and omega-3 fatty acids on diabetic retinopathy and age-related macular degeneration

**DOI:** 10.1016/j.conctc.2023.101184

**Published:** 2023-07-05

**Authors:** Emily Sammons, Louise Bowman, William Stevens, Georgina Buck, Karl Wallendszus, Imen Hammami, Sarah Parish, Jane Armitage, ASCEND Collaborative Group

**Affiliations:** a Medical Research Council Population Health Research Unit, Clinical Trial Service Unit & Epidemiological Studies Unit, University of Oxford, Nuffield Department of Population Health, Richard Doll Building, Old Road Campus, Roosevelt Drive, Oxford, OX3 7LF, UK; b Medical Research Council Population Health Research Unit, Clinical Trial Service Unit & Epidemiological Studies Unit, University of Oxford, Nuffield Department of Population Health, Big Data Institute, Old Road Campus, Roosevelt Drive, Oxford, OX3 7LF, UK

**Keywords:** Diabetic retinopathy, Age-related macular degeneration, Aspirin, Omega-3 fatty acids, Randomized trial

## Abstract

**Background:**

Aspirin and omega-3 fatty acids (FAs) have potential disease-modifying roles in diabetic retinopathy (DR) and age-related macular degeneration (AMD), but randomized evidence of these effects is limited. We present the rationale and baseline characteristics of ASCEND-Eye, a sub-study of the double-blind, 2x2 factorial design, randomized placebo-controlled ASCEND (A Study of Cardiovascular Events iN Diabetes) trial of 100 mg aspirin daily and, separately, 1g omega-3 FAs daily for the primary prevention of serious cardiovascular events, in 15,480 British adults, aged 40 years or older with diabetes.

**Methods:**

Eye events will be derived from three sources: 1) participant follow-up questionnaires from ASCEND, 2) electronic NHS Diabetic Eye Screening Programme (DESP) data and 3) responses to the National Eye Institute's Visual Function Questionnaire-25 (NEI-VFQ-25) sent to a subset of participants after the main trial ended. Analytic cohorts and outcomes relevant to these data sources are described. The primary outcome is referable diabetic eye disease, a secondary outcome is incident AMD events.

**Results:**

Participant-reported events were ascertained for the full cohort of randomized individuals who were followed up over 7.4 years in ASCEND (n = 15,480). Linked DESP data were available for 48% of those (n = 7360), and 57% completed the NEI-VFQ-25 (n = 8839). The baseline characteristics of these three cohorts are presented.

**Discussion:**

Establishing the risks and benefits of drugs commonly taken by people with diabetes, the elderly, or both, and finding new treatments for DR and AMD is important. ASCEND-Eye provides the opportunity to evaluate the effect of aspirin and, separately, omega-3 FAs for both conditions.

**Study registration:**

Eudract No. 2004-000991-15; Multicentre Research Ethics Committee Ref No. 03/8/087; ClinicalTrials.gov No. NCT00135226; ISRCTN No. ISRCTN60635500.

## 1 Introduction

Diabetic retinopathy (DR) and age-related macular degeneration (AMD) are leading causes of registerable blindness among elderly and working-age adults worldwide [1]. Although a better understanding of the pathogeneses behind these conditions has led to some effective treatments, such as intravitreal anti-angiogenesis medication [2,3], other therapies to slow their progression are continually sought. Candidate treatments of interest include aspirin and omega-3 fatty acids (FA).

### 1.1 Diabetic retinopathy

An early hallmark of DR is retinal capillary occlusion by platelet thrombi, resulting in areas of retinal non-perfusion and microaneurysm formation [4,5]. Observational studies suggest that the anti-thrombotic or anti-inflammatory effects of aspirin might protect against capillary occlusion [[6], [7], [8], [9], [10], [11], [12]]; however, supportive randomized evidence is limited. The Early Treatment Diabetic Retinopathy Study (ETDRS; n = 3711) found that 650 mg aspirin daily did not prevent the development of high-risk retinopathic features, but provided no evidence of aspirin's effect in early disease due to the trial's eligibility criteria [13,14]. In contrast, the Dipyridamole Aspirin Microangiopathy of Diabetes study (DAMAD; n = 475), compared the effects of aspirin in people with minimal disease at baseline [15]. Those allocated 990 mg aspirin daily or dual antiplatelet therapy for three years, had marginally fewer microaneurysms per year than the placebo group, but the longer-term prognostic significance of this was unclear. Despite extensive randomized data on the effects of aspirin on cardiovascular disease [16], no other trials have specifically reported on DR outcomes.

Owing to some interconnected pathways, omega-3 FAs may complement the actions of aspirin on DR. Under adverse systemic conditions, cyclooxygenase (COX) and lipoxygenase enzymes release polyunsaturated fatty acids from retinal tissue stores and convert them into compounds with localised vaso- and neuroactive properties [17]. Mediators derived from docosanoid intermediaries of metabolised omega-3 FAs tend to be anti-inflammatory, vasodilating and anti-angiogenic and therefore, it has been suggested that omega-3 FAs supplementation would be beneficial for DR [17]. In the RETIPON trial of 467 high-risk adults with insulin-treated type II diabetes, microalbuminuria and non-proliferative DR, randomization to 600 mg alpha-linolenic acid daily (which is converted into omega-3 FAs) did not prevent clinically-significant macular oedema, but the study was underpowered to draw a robust conclusion [18]. Although several large trials have assessed the effects of omega-3 FAs [19], none have reported on eye outcomes.

### 1.2 Age-related macular degeneration

Aspirin has been considered of potential benefit in AMD owing to its complementary, non-selective and irreversible inhibitive effects on two isoforms of the COX enzyme: COX-1 and COX-2. The long-term suppression of platelet aggregation via acetylation of COX-1 slows atherosclerotic cardiovascular disease progression, which might also protect against retinal arteriolar narrowing and the deposition of lipids in Bruch's membrane [20,21]. Meanwhile, COX-2 inhibition may reduce the platelet-mediated release of vascular endothelial growth factors [[21], [22], [23]] and the expression of pro-inflammatory prostaglandins involved in the biogenesis of drusen [[24], [25], [26], [27], [28], [29]]. Collectively, these actions might protect against choroidal neovascularisation and disruption of the retinal pigment epithelium. Conversely, the vasoconstrictive effect of COX-2-suppressed prostacyclin synthesis may cause hypoxia in older people with narrowed choroidal blood vessels, becoming the stimulus for neovascularisation and the development of the wet form of AMD [30].

AMD shares some of the processes implicated in the pathogenesis of DR, except that the main vascular network affected is the choriocapillaris instead of the retinal capillaries [17]. Thus, omega-3 FAs have been considered of potential benefit in AMD for the same reasons they have been proposed for DR. The chemical structure of omega-3 FAs might also preserve the elasticity of choriocapillaris vessels, maintaining perfusion, fluid permeability and nutrient transport to retinal tissues, which could be important in ageing eyes.

Observational studies of the association between aspirin and, separately, omega-3 FAs and AMD are difficult to interpret due to the complex, multifactorial nature of the disease, where the level of risk conferred by different genetic, inflammatory, vascular or environmental variables is difficult to quantify. Randomized evidence for these treatments are also limited. In the case of aspirin, the subsidiary analyses of incident AMD from two large cardiovascular primary prevention trials found a non-significant trend towards a protective effect, but had too few events among their relatively young study population to draw robust conclusions [28,31]. A sub-study of the ASPirin in Reducing Events in the Elderly (ASPREE) randomized placebo-controlled trial of 100 mg daily aspirin in approximately 5000 healthy people aged 70 years or older, has used retinal photography to examine the incidence and progression of AMD over a 5-year follow-up [32,33]. The results are expected soon. Meanwhile, three randomized placebo-controlled trials of omega-3 FAs found no statistically-significant protective effects against progressive AMD, but all were underpowered to detect smaller but potentially important effects [[34], [35], [36]].

The ASCEND (A Study of Cardiovascular Events iN Diabetes) randomized trial [[37], [38], [39]] conducted in the UK between 2011 and 2017, including 15,480 people with diabetes but no evidence of cardiovascular disease, provides the opportunity to investigate the effects of aspirin and, separately, omega-3 FAs on DR and AMD in a population with contemporary standards of glycaemic and blood pressure control. The ASCEND-Eye sub-study links to national retinopathy screening records with the primary aim of determining the effect of study treatment on time to first referable diabetic eye disease post-randomization. A secondary aim is to compare the effect of each treatment on incident AMD events.

## 2 Methods

### 2.1 Organisation, data management and confidentiality

The Clinical Trial Service Unit at the University of Oxford coordinates ASCEND-Eye and has overall responsibility for its administration. All research staff comply with the requirements of the General Data Protection Regulation and Data Protection Act 2018 concerning the collection, storage and processing of personal information. Privacy rights are readily available via the Privacy Notice on the ASCEND website: https://ascend.medsci.ox.ac.uk/about/privacy-notice-ascend-eye.

### 2.2 ASCEND

The design of ASCEND has been described in detail elsewhere [[37], [38], [39], [40]]. Briefly, the trial used mail-based methods to randomize and follow up 15,480 participants with diabetes from the UK, who were recruited between 2005 and 2011. Men and women with type 1 or type 2 diabetes mellitus aged at least 40 years with no previous history of cardiovascular disease, no clear contraindication to aspirin and no other significant medical condition that could limit adherence to the trial regimen for five years were eligible. After a 2-month placebo run-in, participants were randomized in a 2 × 2 factorial, blinded design between 100 mg aspirin daily and matching placebo and, separately, between 1g omega and 3 FAs (containing 0.41g eicosapentaenoic acid and 0.34g docosahexaenoic acid) daily and matching placebo. Randomization was in a 1:1 ratio in both treatment arms; a minimization algorithm was used to ensure balance for important prognostic variables (age, sex, duration of diabetes, history of treated hypertension, smoking status, ethnic origin, total cholesterol, HbA1c, and urinary albumin/creatinine ratio) between active and placebo groups in each arm [41]. Six-monthly follow-up questionnaires sought information about the trial's main cardiovascular endpoints, other serious adverse events, bleeding episodes requiring medical attention, and adherence to the study treatments. Participants could also report this information directly to the coordinating office via a dedicated telephone service.

### 2.3 ASCEND-Eye

ASCEND-Eye will generate information on eye health via three different data sources:

#### 2.3.1 Participant-reported outcomes

Participants could report new eye bleeding, AMD and diabetic macular oedema events on their six-monthly ASCEND follow-up questionnaires (see supplementary materials). Supporting evidence was sought from participants’ general practitioners and these events were adjudicated against pre-specified criteria by trial clinicians who were blind to the study treatment allocations. Incident eye events could also be reported on a Visual Function Questionnaire (VFQ) sent to a subset of participants after the scheduled treatment period in ASCEND was completed. However, events originating from this source were not adjudicated. Allowance for this difference in the event verification is made in the definitions of outcomes in a pre-specified Data Analysis Plan (DAP; see supplementary materials).

#### 2.3.2 Linkage to the NHS Diabetic Eye Screening Programme

Longitudinal information on diabetic retinopathy (R) and maculopathy (M) grades and best corrected visual acuity was sought by electronic linkage to NHS Diabetic Eye Screening Programme (DESP) data in England and Wales. The NHS currently invites all GP-registered persons aged 12 years and above with diabetes to attend an annual screening appointment, which entails flash colour and red-free digital retinal photography through a dilated pupil [42]. Images are graded using standardised coding practices by trained and quality-controlled graders, as defined by the National Screening Committee (NSC) [43]. Those with no retinopathy or background DR (R_0_ or R_1_, respectively) or no maculopathy (M_0_) maintain a yearly screening schedule, whereas those with pre-proliferative or proliferative lesions (R_2_ or R_3a/s_, respectively) or maculopathy (M_1_) are referred to care pathways overseen by a Consultant Ophthalmologist with medical retina expertise, for further assessment and treatment [44,45]. NHS numbers were chosen as the sole linkage identifier because they are a unique pseudonym that remains the same throughout an individual's lifetime and have previously been shown to be valid and complete for the majority of secondary and primary care records in England [46]. Due to the complexity of obtaining separate regulatory approvals in Scotland and Northern Ireland, a pragmatic decision was made to exclude the 3% of participants who resided in these countries (n = 506). Those who withdrew consent during ASCEND (n = 60), those without an NHS number (n = 6), and those who attended a GP practice that did not register their data with Public Health England (n = 1252) were also excluded, leaving 13,656 participants who were eligible for linkage. Without a single central repository of data in England, separate applications were sent to access data from 58 DESPs in England and the Diabetic Eye Screening Service for Wales. Where there was a lack of expertise or capacity to prepare a linkage script, DESPs were given the option of allowing their eye screening software provider (Northgate Ltd or Health Intelligence Ltd) to do so on their behalf.

#### 2.3.3 Visual Function Questionnaire

All surviving participants who agreed to follow up at the end of ASCEND were sent a VFQ (see supplementary materials). The questionnaire consisted of two parts: a bespoke page of questions which explicitly sought incident diagnoses of serious eye conditions, including AMD, cataracts, glaucoma and retinal vein thrombosis, followed by the standard National Eye Institute Visual Function Questionnaire-25 (NEI-VFQ-25) [47].

### 2.4 Preliminary blinded analyses of the DESP-linkage data

After the DESP-linked data were obtained, preliminary blinded analyses were conducted to assess the extent of missing data and to investigate laterality of the retinopathy vs. maculopathy components over time. Due to changing geographical boundaries of the NHS DESPs during ASCEND, and proximity of the trial's recruitment (2005) to the initiation and expansion of the national service since 2003, there was incomplete longitudinal coverage of participants' screening records before randomization. Therefore, it became apparent that analyses of the DESP data restricted by pre-randomization disease status would involve a much smaller cohort with less power to detect differences between treatment groups. We also ignored responses from ASCEND's randomization questionnaire about pre-existing DR because it was less well-defined than, and discordant with, the presence of DR recorded by the screening service; however, this information is presented in the baseline characteristics tables. Hence, the primary efficacy outcome will be of time to the first occurrence of referable disease, defined in the next section, among the largest cohort with in-trial eye screening data; secondary efficacy outcomes involving the linkage data, will compare incident disease among those with a baseline DESP record. These exploratory blinded assessments also informed definitions of the treatment duration, baseline and censoring dates, which are presented in the DAP (see supplementary materials).

### 2.5 ASCEND-Eye outcomes and analysis cohorts

A DAP was published on the ASCEND trial website (https://ascend.medsci.ox.ac.uk/) before unblinding the ASCEND-Eye results, except for previously published results for sight-threatening eye bleeding events, which were included in the composite primary safety outcome of “major haemorrhage” for ASCEND [37].

The DAP defines three discrete participant populations, reflecting the three data sources described earlier. The hierarchy of outcomes to be assessed in ASCEND-Eye and their data source is summarised in Table 1. Outcomes comprising participant-reported events, such as AMD and eye bleeds, shall include every randomized participant in ASCEND (n = 15,480). In contrast, outcomes derived from the DESP-linkage data (n = 7360) and NEI-VFQ-25 (n = 8839) involve subsets of the full population. The linkage cohort will be further divided for specific analyses. Given the overlapping data sources and analysis populations for some of the outcomes, we intend to publish separate manuscripts for the AMD, DESP-linkage and NEI-VFQ-25 analyses.

**Table 1:** Summary of outcomes, data sources and analysis populations.

Data source (Hierarchy and cohort size)	Outcome Definition
**Efficacy**	
**DESP-linked data**	
1° efficacy (n = 7360)	Referable diabetic eye disease (R_2_, R_3a/s_ or M_1_) for those with in-trial retinopathy data
2° efficacy (n = 2558)	Referable diabetic eye disease restricted to those with a baseline record (R_0_ or R_1_) and M_0_ →(R_2_, R_3a/s_ or M_1_)
2° efficacy (n = 2852)	Any progression in retinopathy grade

**Participant questionnaires**^a^	
2° efficacy (n = 15,480)	Incident diagnoses of AMD

**NEI-VFQ-25**	
2° efficacy (n = 8839)	Composite scores from the NEI-VFQ-25

a Either ASCEND follow-up questionnaires or the VFQ.

#### 2.5.1 Primary efficacy outcome

DESP-linkage data will be used to compare time to the first post-randomization occurrence of referable disease, defined as the composite of referable diabetic retinopathy (R_2_ or R_3a/s_) or referable diabetic maculopathy (M_1_) in either eye, based on the NSC grading criteria [45,48].

#### 2.5.2 Secondary efficacy outcomes

• Participant-reported events derived from ASCEND follow-up questionnaires (adjudicated) or the VFQ (unadjudicated) will be used to compare time to a post-randomization confirmed or unrefuted incident diagnosis of AMD.
• DESP-linkage data will be used to compare:
  o Time to the first post-randomization referable disease in those without DR (R_0_) or with only background diabetic retinopathy (R_1_), and no maculopathy (M_0_), in both eyes at baseline; Analysed within the following strata of baseline retinopathy and as an overall stratified analysis: R_0_/R_0_, R_0_/R_1_ or R_1_/R_0_ and R_1_/R_1_.
  o Time to the first post-randomization progression in retinopathy grade in either eye, where progression is defined as an increase by one step or more in R grade, based on the NSC scoring protocol for retinopathy, excluding those with proliferative disease (R_3a/s_/R_3a/s_) in both eyes on their baseline record; Analysed within the following strata of baseline retinopathy and as an overall stratified analysis: R_0_/R_0_, R_0_/R_1_ or R_1_/R_0_, R_1_/R_1_ and >R_1_/R_any_
• Composite scores from the NEI-VFQ-25 in the subset of participants who returned a VFQ.

#### 2.5.3 Primary safety outcome

Participant-reported events derived from ASCEND follow-up questionnaires will be used to compare time to the first post-randomization confirmed or unrefuted incident diagnosis of sight-threatening eye bleeding, defined as clinically-significant bleeding in the eye resulting in unresolved visual loss and/or requiring an urgent intervention such as laser photocoagulation, vitreoretinal surgery or intraocular injection(s) of anti-angiogenesis therapies.

#### 2.5.4 Other outcomes

Some tertiary and sensitivity analyses are also planned, including time-to-event analyses of incident diabetic maculopathy and cross-sectional comparisons of duplex retinopathy grades on the last available eye screening record during the scheduled treatment period. These are defined in more detail in the DAP (see supplementary materials).

Based on blinded preliminary analyses there were 1061 primary efficacy outcomes, providing 81% power to detect 15% proportional reductions in the incidence of referable diabetic eye disease at 2P < 0.05 (Table 2). ASCEND-Eye has insufficient power to detect plausible effects of aspirin or omega-3 FAs on AMD because there are too few events among the relatively young population of ASCEND (mean baseline age = 63.3) [37]. From blinded analyses, there were 260 AMD events in total, providing only 62% power to detect a 25% proportional difference in the risk of incident AMD at 2P < 0.05.

**Table 2:** Power of ASCEND-Eye to detect different effects of the interventions on referable disease among the cohort of 7360 participants with linkage data available during the scheduled treatment period.

Proportional reduction of risk in the active arm compared to the placebo arm	N with event		Power at 2P = 0.05	Power at 2P = 0.01
	Active (N approx. 3680)	Placebo (N approx. 3680)		
25%	455	606	100%	99%
20%	472	589	97%	90%
15%	487	574	81%	60%
10%	503	558	44%	22%

### 2.6 Statistical methods

All comparisons of the main efficacy outcomes will be between those allocated aspirin daily *versus* matching placebo, and separately, omega-3 FAs supplement daily versus matching placebo, during the scheduled treatment period. Every randomized participant will be compared, regardless of whether they took all, some or none of their allocated treatment (i.e. intention-to-treat analyses). The factorial design of ASCEND does not affect the statistical sensitivity with which the effects of each treatment arm can be assessed [49,50]. Moreover, no clinically-significant interactions between the study treatments are anticipated [51]. Comparisons of the aspirin arm will therefore be made without stratification by omega-3 FAs allocation (and vice versa for omega-3 FAs analyses). All analyses, conducted using SAS version 9.4, will be of time from randomization to the first occurrence of each outcome, except for the cross-sectional comparisons of duplex retinopathy grades and scores from the NEI-VFQ-25.

Logrank [50,52] methods and, where appropriate, stratified logrank [53] methods, will be used in time-to-event analyses to calculate the two-sided P-values between the active and placebo groups of each treatment arm. Average event rate ratios and their 95% confidence intervals will be calculated using the one-step method from the “observed minus expected” numbers of events (O-E) and their variances (V) outputted from the SAS LIFETEST procedure (event rate ratio = exp(O-E/V)) [52]. The results will be represented graphically in the form of Kaplan-Meier and Forest plots, respectively.

Proportional ordinal logistic regression models will be fitted to composite scores from the NEI-VFQ-25 grouped into a 5-point ordinal scale as the outcome variable: ≥90, 80–89, 70–79, 60–69 and < 60. A common odds ratio with 95% confidence intervals will be used to interpret the average effect size over the total ordinal scale, of allocation to the active and placebo groups in each treatment arm.

A two-tailed P-value <0.05 will be considered to indicate statistical significance for the primary efficacy and safety outcomes. No adjustment for multiplicity shall be made for the secondary or tertiary analyses. The results from these analyses will be interpreted cautiously and in the context of existing studies, the number of comparisons undertaken, the number of events, and if the upper and lower confidence intervals are further away from zero (which would be associated with a more extreme p-value).

## 3 Results

Multi-centre Research Ethics Committee (MREC) approval was granted for ASCEND-Eye by the North West MREC in October 2016. Separate research governance approvals were later granted by the Health Research Agency, Research Advisory Committees for Public Health England and Public Health Wales, and individual controllers of the NHS DESP data.

### 3.1 Baseline characteristics of the full ASCEND population

The clinical and demographic characteristics of the complete randomized population in ASCEND (n = 15,480) are shown in supplementary Figures S1 and S2 and Table S1. At baseline, they had a mean age of 63.3 years (SD 9.2), 63% were male, and they had been diagnosed with diabetes (94% type 2) for a median of 7 years before randomization. 16% of the participants managed their diabetes through dietary measures alone, 58% used oral hypoglycaemic agents, but not insulin, and 25% were insulin-treated. The majority (82%) were overweight or obese based on their BMI, and 62% reported taking treatment for hypertension. The baseline characteristics between treatment arms were well-balanced for characteristics associated with an increased risk of AMD, including age, smoking status and sex.

### 3.2 Baseline characteristics of the DESP-Linked Cohort

**Fig. 1:**
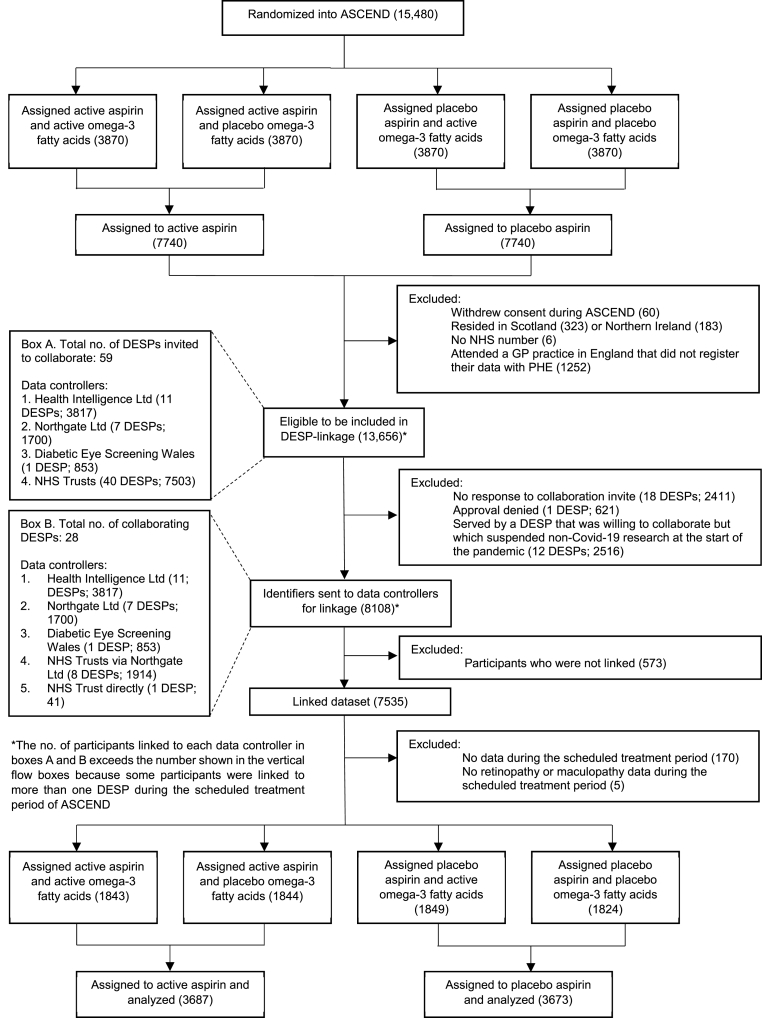
Consort Diagram for the DESP-Linked Cohort (Aspirin Arm) FAs = Fatty acids.

Fig. 1 and S3 are consort diagrams for the subset of participants who will be included in the primary efficacy analyses. Out of 13,656 participants who were eligible for the linkage exercise, identifiers were sent to be linked for 8108 participants who attended one of 28 collaborating DESPs, and 7535 were successfully matched. 2411 (17.7%) attended one of 18 DESPs that never responded to invitations to collaborate, 2516 (18.4%) attended one of 12 DESPs that suspended the processing of all non-Covid-19 research applications in March 2020, and 621 (4.5%) attended one DESP that declined to collaborate. After excluding those without data during the scheduled treatment period of ASCEND, the cohort size for the primary efficacy analyses included 7360 participants.

The mean age of participants in the linkage cohort was 63.5 years (SD 8.9), 62% were male, and they had been diagnosed with diabetes (95% type 2) for a median of 6 years before randomization. Of those for whom the data were available, 62% reported taking treatment for hypertension, 55% were former/current smokers, and 85% were overweight or obese (Table 3 is a truncated version of the full baseline characteristics shown in Table S2). 34% reported regular aspirin use before entering ASCEND, which they stopped to take part in the trial, 58% were on prescribed ACE-inhibitor therapy, and 76% were taking statins. The baseline characteristics between treatment arms were well-balanced for characteristics associated with an increased risk of DR, including duration of diabetes, glycaemic control, blood pressure, urinary albumin:creatinine ratio and smoking status. The composition of those with linkage data was representative of the full ASCEND population; however, there was statistically significant heterogeneity between those included in and excluded from the linkage exercise for some characteristics (Table S3). Those excluded were marginally younger, with a longer duration of diabetes, and more had type 1 diabetes. More frequently, they had less favourable lipid and renal parameters, were non-smokers, or had worse glycaemic control. There were no significant differences in sex, blood pressure, BMI or ethnicity.

**Table 3:** Baseline characteristics of the DESP-Linked population.

Baseline Characteristic	Aspirin				Omega-3 FAs				Overall (n = 7360)	
	Active (n = 3687)		Placebo (n = 3673)		Active (n = 3692)		Placebo (n = 3668)			
**Age at randomization (years)**										
Mean (SD)	63.5 ± 8.9		63.5 ± 9.0		63.5 ± 8.9		63.5 ± 9.0		63.5 ± 8.9	

**Sex**										
Male	2314	(63%)	2280	(62%)	2301	(62%)	2293	(63%)	4594	(62%)
Female	1373	(37%)	1393	(38%)	1391	(38%)	1375	(38%)	2766	(38%)

**Type of diabetes**^a^										
Type 1	186	(5%)	187	(5%)	181	(5%)	192	(5%)	373	(5%)
Type 2	3501	(95%)	3486	(95%)	3511	(95%)	3476	(95%)	6987	(95%)

**Duration of diabetes (years)**										
Median (IQR)	7 (3–12)		6 (3–12)		7 (3–12)		6 (3–12)		6 (3–12)	

**Systolic blood pressure (mmHg)**^b^										
Mean (SD)	136.1 ± 15.2		136.0 ± 15.3		135.9 ± 15.2		136.1 ± 15.2		136.1 ± 15.2	

**Diastolic blood pressure (mmHg)**^b^										
Mean (SD)	77.0 ± 9.4		77.2 ± 9.4		77.1 ± 9.3		77.1 ± 9.5		77.1 ± 9.4	

**Body mass index (kg/m**^**2**^**)**^c^										
Mean (SD)	30.7 ± 6.2		30.6 ± 6.2		30.6 ± 6.1		30.7 ± 6.2		30.7 ± 6.2	

**HbA1c**										
DCCT (%) mean (SD)	7.1 ± 1.2		7.1 ± 1.2		7.1 ± 1.2		7.1 ± 1.2		7.1 ± 1.2	
IFCC (mmol/mol) mean (SD)	54.3 ± 12.9		54.3 ± 12.5		54.4 ± 12.6		54.2 ± 12.7		54.3 ± 12.7	

**Cigarette smoking**										
Current	281	(8%)	296	(8%)	294	(8%)	283	(8%)	577	(8%)
Former	1703	(46%)	1714	(47%)	1728	(47%)	1689	(46%)	3417	(46%)
Never	1656	(45%)	1620	(44%)	1629	(44%)	1647	(45%)	3276	(45%)
Unknown	47	(1%)	43	(1%)	41	(1%)	49	(1%)	90	(1%)

**CKD-EPI estimated GFR (ml/min/1.73m** [2]**)**^d^										
Mean (SD)	85.0 ± 20.6		84.3 ± 20.7		84.7 ± 20.7		84.7 ± 20.6		84.7 ± 20.6	

**Urinary albumin:creatinine ratio (mg/mmol)**^e^										
Median (IQR)	0.6 (0.0–1.3)		0.5 (0.2–1.3)		0.6 (0.2–1.3)		0.5 (0.0–1.3)		0.5 (0.2–1.3)	

**Ethnic origin**										
White	3553	(96%)	3530	(96%)	3554	(96%)	3529	(96%)	7083	(96%)

DCCT = Diabetes Control and Complications Trial; FAs = Fatty acids; GFR = Glomerular Filtration Rate; HDL=High-density lipoprotein; IFCC =International Federation of Clinical Chemistry; IQR = Interquartile range; SD = Standard Deviation.

Figures presented are counts with percentages unless otherwise stated. Percentages may not total 100 because of rounding.

a The presence of type 2 diabetes was based on a broad clinical definition involving the participant's age at the diagnosis of diabetes, the use of insulin within one year after diagnosis, and the body-mass index.

b From blood and urine consent forms, generally before randomization.

c The body-mass index (the weight in kilograms divided by the square of the height in metres) was based on values for height and weight the participants reported on their randomization questionnaires.

d Calculated from blood cystatin c concentration using the CKD-EPI formula [56].

e There was an analysis rule in ASCEND which stated that those with a below detectable threshold albumin component of their urinary albumin creatinine ratio would be recorded as zero. This applied to just over 25% of participants in the active group of the aspirin arm, and to just under 25% of participants in the placebo group. Hence the interquartile range included zero.

### 3.3 Baseline characteristics of the VFQ cohort

**Fig. 2:**
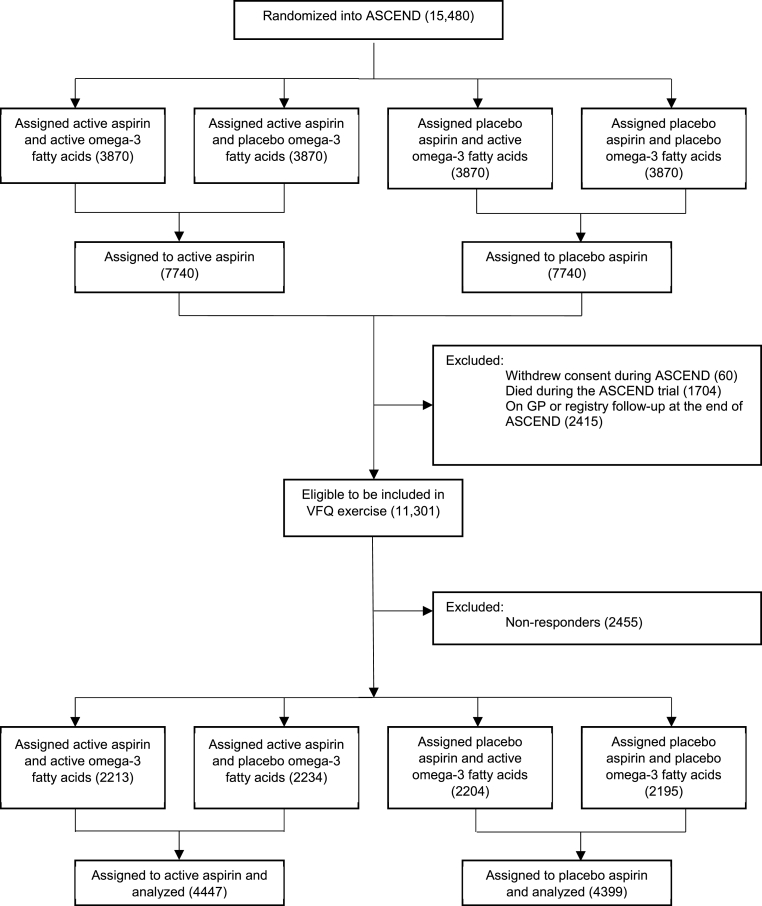
Consort Diagram for the VFQ Cohort (Aspirin Arm) FAs = Fatty acids.

Fig. 2 and S4 are consort diagrams for the VFQ cohort in the aspirin arm and omega-3 FAs arm, respectively. Those who withdrew consent (n = 60), died during the ASCEND trial (n = 1704), or were on GP or registry follow-up at the end of ASCEND (n = 2415) were excluded from taking part, leaving 11,301 (73.0% of the randomized ASCEND population), who were eligible to be sent a questionnaire. Of those, 8846 (78%) participants returned the questionnaire; 8839 responded to both parts of the questionnaire, whilst 7 answered questions about eye diagnoses but did not complete the NEI-VFQ-25. All 8846 were included in the VFQ baseline characteristic comparisons we now describe.

The mean age of participants in the VFQ cohort was 62.5 years (SD 8.3), 63% were male and they had been diagnosed with diabetes (94% type 2) for a median of 7 years before randomization. Of those for whom the data were available, 61% reported taking treatment for hypertension, 51% were former/current smokers, and 85% were overweight or obese (Table 4 is a truncated version of the full baseline characteristics shown in Table S4). The baseline characteristics between treatment arms were well-balanced. Overall, the composition of respondents was representative of the full ASCEND population; however, there was statistically significant heterogeneity between those included in and excluded from the exercise for some characteristics (Table S5). Those excluded were slightly older, less affluent, with a longer duration of diabetes, and fewer had type 1 diabetes. More frequently, they reported having hypertension at baseline, had less favourable lipid and renal parameters, were current or former smokers, or had worse glycaemic control. There was no significant heterogeneity for sex or pre-trial use of antiplatelet therapy.

**Table 4:** Baseline characteristics of the VFQ population.

Baseline Characteristic	Aspirin				Omega-3 FAs				Overall (n = 8846)	
	Active (n = 4447)		Placebo (n = 4399)		Active (n = 4417)		Placebo (n = 4429)			
**Age at randomization (years)**										
Mean (SD)	62.4 ± 8.3		62.5 ± 8.3		62.4 ± 8.3		62.5 ± 8.3		62.5 ± 8.3	

**Sex**										
Male	2785	(63%)	2744	(62%)	2758	(62%)	2771	(63%)	5529	(63%)
Female	1662	(37%)	1655	(38%)	1659	(38%)	1658	(37%)	3317	(38%)

**Type of diabetes**^a^										
Type 1	270	(6%)	289	(7%)	288	(7%)	271	(6%)	559	(6%)
Type 2	4177	(94%)	4110	(93%)	4129	(94%)	4158	(94%)	8287	(94%)

**Duration of diabetes (years)**										
Median (IQR)	7 (3–12)		6 (3–12)		7 (3–12)		6 (3–12)		7(3–12)	

**Systolic blood pressure (mmHg)**^b^										
Mean (SD)	135.7 ± 15.1		135.9 ± 14.9		135.9 ± 15.1		135.7 ± 14.9		135.8 ± 14.9	

**Diastolic blood pressure (mmHg)**^b^										
Mean (SD)	77.3 ± 9.3		77.6 ± 9.1		77.5 ± 9.2		77.4 ± 9.1		77.4 ± 9.2	

**Body mass index (kg/m**^**2**^**)**^c^										
Mean (SD)	30.8 ± 6.3		30.4 ± 5.9		30.6 ± 6.1		30.6 ± 6.1		30.6 ± 6.1	

**HbA1c**										
DCCT (%) mean (SD)	7.1 ± 1.1		7.1 ± 1.1		7.1 ± 1.1		7.1 ± 1.1		7.1 ± 1.1	
IFCC (mmol/mol) mean (SD)	53.9 ± 12.1		54.2 ± 12.2		54.2 ± 12.1		53.9 ± 12.3		54.0 ± 12.2	

**Cigarette smoking**										
Current	264	(6%)	291	(7%)	278	(6%)	277	(6%)	555	(6%)
Former	1995	(45%)	1952	(44%)	1959	(44%)	1988	(45%)	3947	(45%)
Never	2139	(48%)	2104	(48%)	2131	(48%)	2112	(48%)	4243	(48%)
Unknown	49	(1%)	52	(1%)	49	(1%)	52	(1%)	101	(1%)

**CKD-EPI estimated GFR (ml/min/1.73m** [2]**)**^d^										
Mean (SD)	87.9 ± 19.6		87.7 ± 19.9		87.7 ± 20.2		87.8 ± 19.3		87.8 ± 19.8	

**Urinary albumin:creatinine ratio (mg/mmol)**^e^										
Median (IQR)	0.5 (0.0–1.2)		0.5 (0.0–1.1)		0.5 (0.0–1.2)		0.5 (0.0–1.1)		0.5 (0.0–1.1)	

**Ethnic origin**										
White	4309	(97%)	4255	(97%)	4279	(97%)	4285	(97%)	8564	(97%)

DCCT = Diabetes Control and Complications Trial; FAs = Fatty acids; GFR = Glomerular Filtration Rate; HDL=High-density lipoprotein; IFCC =International Federation of Clinical Chemistry; IQR = Interquartile range; SD = Standard Deviation.

Figures presented are counts with percentages unless otherwise stated. Percentages may not total 100 because of rounding.

a The presence of type 2 diabetes was based on a broad clinical definition involving the participant's age at the diagnosis of diabetes, the use of insulin within one year after diagnosis, and the body-mass index.

b From blood and urine consent forms, generally before randomization.

c The body-mass index (the weight in kilograms divided by the square of the height in metres) was based on values for height and weight the participants reported on their randomization questionnaires.

d Calculated from blood cystatin c concentration using the CKD-EPI formula [56].

e There was an analysis rule in ASCEND which stated that those with a below detectable threshold albumin component of their urinary albumin creatinine ratio would be recorded as zero. This applied to just over 25% of participants in the active group of the aspirin arm, and to just under 25% of participants in the placebo group. Hence the interquartile range included zero.

## 4 Discussion

ASCEND-Eye will be one of the largest randomized assessments of the effects of aspirin and omega-3 FAs on eye outcomes. Aspirin is the world's most commonly used drug due to its proven efficacy in the management of occlusive cardiovascular disease [16]. It is also under investigation as a chemopreventative therapy for colorectal and other types of cancer [54]. In addition, it has been estimated that approximately 8% of adults regularly supplement their diet with omega-3 FAs [55]. Evaluating the balance of risks and benefits of aspirin and omega-3 FAs on eye health is important given their potential as low-cost interventions and widespread use by individuals with diabetes, those who are elderly, or both, who are at risk of DR and AMD, respectively.

The strengths of ASCEND-Eye include its randomized design, large size, long duration, and well-defined exposure to both treatments, as well as good completeness of follow-up and compliance in the parent ASCEND trial. Limitations include its reliance on participant reports for the AMD, diabetic macular oedema and eye bleeding outcomes without imaging such as fundus photography or optical coherence tomography, which would enable more accurate phenotyping. Therefore, it will not be possible to ascertain early versus late AMD events. Randomization ensures that these factors should be balanced across treatment groups and should not introduce bias in the comparisons. Although ASCEND-Eye will be underpowered to confirm statistically-significant effects of the treatments on AMD, it overcomes the limitations of previous observational studies and will contribute reliable data to future meta-analyses. Finally, the generalisability of the results in a real-world setting may be limited by a lack of ethnic diversity and the relative under-representation of women in ASCEND.

Extensive efforts, predating the Covid-19 pandemic, were made to involve every English DESP in the linkage activity. Therefore, it is frustrating that the data for 5548 participants who gave their consent could not be obtained for bureaucratic reasons. Despite these challenges, ASCEND-Eye is the largest randomized trial of aspirin and the first prospective test of omega-3 FAs on DR to date. To the best of our knowledge, application of the NEI-VFQ-25 also represents the largest survey of vision-targeted health-related quality of life in people with diabetes yet.

## 5 Conclusion

Based on an existing large randomized trial, ASCEND-Eye will provide reliable evidence of the effects of aspirin and omega-3 FAs on DR and AMD. The results, which are important and relevant to those living with diabetes and elderly individuals, are anticipated to be available by late 2023.

## Data availability

All requests for data sharing should be addressed to the corresponding author and will be handled in line with the data access and sharing policy of the Nuffield Department of Population Health, University of Oxford (www.ndph.ox.ac.uk/about/data-access-policy).

## Financial statement

The ASCEND study was funded by grants to the University of Oxford from the British Heart Foundation (SP/03/002, SP/08/010/25939, SP/14/3/31114, PG/05/013/18296). The Clinical Trial Service Unit at the University of Oxford receives support from the UK Medical Research Council (which funds the MRC Population Health Research Unit in a strategic partnership with the University of Oxford, MC_UU_00017/3, MC_UU_00017/5), the British Heart Foundation, and Cancer Research UK. The supply of aspirin and matching placebo was provided by Bayer Healthcare AG, and the supply of omega-3 FA and matching placebo capsules by Abbott Products Operations AG (formerly Solvay Pharmaceuticals), with some funding from each company to cover drug packaging. Funding for the ASCEND-Eye study was obtained from the Macular Society and British Heart Foundation. The design of the study and collection, analysis, and interpretation of data and writing the manuscript were carried out independently of the funding bodies. For the purpose of open access, the authors have applied a Creative Commons Attribution (CC BY) license to any Author Accepted Manuscript version arising.

The Clinical Trial Service Unit at the University of Oxford receives support from the UK Medical Research Council (which funds the MRC Population Health Research Unit in a strategic partnership with the University of Oxford, MC_UU_00017/3, MC_UU_00017/5), the British Heart Foundation and Cancer Research UK. ES received separate funding from the British Heart Foundation and Macular Society for this work.

Financial sources have no authority over the design of the study and collection, analysis or interpretation of data, or the decision to submit manuscripts for publication.

## Author contributions

JA and LB were co-Principal Investigators of the ASCEND trial, and together with ES, have made substantial contributions to the design of the ASCEND-Eye study. ES obtained regulatory and information governance approvals, established collaborations with NHS Diabetic Eye Screening Programmes and their software providers, and wrote the manuscript we present. WS, KW, GB, SP and IH gave statistical advice and made significant contributions to the data analysis plans for ASCEND-Eye. All authors read and approved the final manuscript.

## Declaration of competing interest

The authors declare that they have no known competing financial interests or personal relationships that could have appeared to influence the work reported in this paper.
