# Supplementary material for: ASCEND-Eye: Rationale, design and baseline characteristics for a sub-study of the ASCEND randomised trial, exploring the effects of aspirin and omega-3 fatty acids on diabetic retinopathy and age-related macular degeneration
Source: Contemp Clin Trials Commun. 2023 Jul 5;35:101184. doi: 10.1016/j.conctc.2023.101184 (PMC10517364; doi:10.1016/j.conctc.2023.101184)
Supplement: Multimedia component 1 [file mmc1.docx]

**Supplementary Appendix**

This supplement contains the following items:

ASCEND-Eye Study Collaborative Group

ASCEND-Eye Data Analysis Plan

ASCEND Follow-up Questionnaire

ASCEND-Eye Visual Function Questionnaire

Figure S1 Consort diagram for the full randomized ASCEND population (Aspirin arm)

Figure S2 Consort diagram for the full randomized ASCEND population (Omega-3 arm)

Figure S3 Consort diagram for the DESP-linked Cohort (Omega-3 arm)

Figure S4 Consort diagram for the VFQ Cohort (Omega-3 arm)

Table S1 Baseline Characteristics of the Full ASCEND Population (Cohort A)

Table S2 Baseline Characteristics of the DESP-Linked Population (Cohort B1)

Table S3 Heterogeneity of baseline characteristics between those included and excluded from the DESP-linked cohort (B1)

Table S4 Baseline Characteristics of the VFQ Responders (Cohort C)

Table S5 Heterogeneity of baseline characteristics between those included and excluded from the VFQ cohort

## ASCEND-Eye Collaborative Group

**Writing Committee**

Emily Sammons (*Corresponding author)*, Louise Bowman, William Stevens, Georgina Buck, Imen Hamammi, Sarah Parish, Jane Armitage

**Steering Committee**

*Chairman:* R Collins, *Study coordinator:* J Armitage; *Clinical coordinator:* L Bowman; *Statisticians:* S Parish, R Peto; *Administrative coordinator:* J Barton; *Lay member:* D Simpson; *Other members:* A Adler, T Aung, C Baigent, HJ Bodansky, A Farmer, R Haynes, R McPherson, M Mafham, HAW Neil, N Samani, P Sleight, P Weissberg.

**Data Monitoring Committee**

*Chair:* P Sandercock, *Members:* H Gerstein, R Gray, C Hennekens.

**Coordinating Office (Clinical Trial Service Unit, Nuffield Department of Population Health, University of Oxford):**

*Administration and support:* J Barton, L Fletcher, K Murphy (coordinators); S Hurley, R Lee, S Pickworth, M Willett, M Wincott.

*Clinical support and adjudication:* J Armitage, L Bowman, M Mafham, E Sammons

*Statistics and computing:* M Lay, S Parish; G Buck, A Murawska, W Stevens, K Wallendszus, A Young, I Hammami

*Research support:* K Melham, G Brown, J Latham-Mollart, A Brewer

**Collaborators**

*Ophthalmologists:* P Scanlon^1^, P Patel^2^

*Public Health England* (now the UK Health Security Agency): M Olson^3^

*Public Health Wales^4^:* J Kay, S Banerjee, L Evans, A Davies, M Griffiths, H Clayton

*Health Intelligence Ltd^5^:* P Kirby. M Pennington^8^, D Clarke

*Northgate Ltd^6^:* J Anslow, A Hallam, J Witts, S Egan, A Wharton

*Cheshire DESP^7^:* A Sachdev, A Derbyshire, E Williamson, K Hepplestone

*East and North Hertfordshire DESP^8^:* S Mithra, S Oliver, P Wiatrak-Olszewska

*Greater Nottingham DESP^9^:* T Gazis, K Alvey, E Wu

*Humber DESP^10^:* H Cook, N Gregory, P Parkinson

*North East London DESP^11^:* J Anderson, L Bolter

*North Nottinghamshire DESP^12^:* P Maharajan, R McFee, L Allsop, D Sowter, D Hodgson

*North Yorkshire DESP^13^:* J Thow, J Featonby, R Furnival

*Oxfordshire DESP^14^:* P Scanlon, H Lipinski, H Benjamin, T McAfee

*South East Sussex DESP^15^:* E Payne, L Still

**Funding**

The Macular Society^16^

British Heart Foundation^17^

1. Department of Ophthalmology, Gloucestershire Hospitals NHS Foundation Trust, Cheltenham General Hospital, Sandford Road, Cheltenham, GL53 7AN
2. Moorfields Eye Hospital, 162 City Road, London, EC1V 2PD
3. Health Information Consulting Ltd, Low Barn, Homestall Lane, Faversham, Kent, ME13 8UT
4. Public Health Wales Research and Evaluation Division, Knowledge Directorate, Floor 5, 2 Capital Quarter, Tyndall Street, Cardiff, CF10 4BZ
5. InHealth Intelligence, Unity House, Road 5, Winsford Industrial Estate, Winsford, Cheshire, CW7 3RB
6. Northgate Public Services (UK) Ltd, Crome Lea Business park, Madingley road, Coton, CB23 7PH
7. Cheshire Diabetic Eye Screening Programme, Eagle Bridge Health and Wellbeing Centre, Dunwoody Way, Crewe, CW1 3AW
8. East and North Hertfordshire Diabetic Eye Screening Programme, Administration Centre H4, Hertford County Hospital, North Road, Hertford, SG14 1LP
9. Greater Nottinghamshire Diabetic Eye Screening Programme, 2nd Floor, Ropewalk House, 113 The Ropewalk, Nottingham, NG1 5DU
10. Humber Diabetic Eye Screening Programme, Alderson House, Hull Royal Infirmary, Hull, HU3 2JZ
11. North East London Diabetic Eye Screening Programme, The Eye Screening Centre, Homerton University Hospital, Homerton Row, London
12. North Nottinghamshire Diabetic Eye Screening Programme, Kings Mill Hospital, Trust Admin Building - Level 2, Mansfield Road, Sutton In Ashfield, Notts, NG17 4JL
13. North Yorkshire Diabetic Eye Screening Programme, 2 Cayley Court, George Cayley Drive, Clifton Moor, York, YO30 4WH
14. Oxfordshire Diabetic Eye Screening Programme, Level 0, West Wing, John Radcliffe Hospital, Headley Way, Headington, Oxford, OX3 9DU
15. South East Sussex Diabetic Eye Screening Programme, Bexhill Hospital, East Sussex Healthcare NHS Trust, Hollier's Hill, Bexhill-on-Sea, TN40 2DZ
16. Macular Society, Crown Chambers, South Street, Andover SP10 2BN
17. British Heart Foundation, Greater London House, 5th Floor, 180 Hampstead Road, London, UK, NW1 7AW

## ASCEND-Eye Data Analysis Plan

**ASCEND-Eye**

**Data Analysis Plan**

**Version 1.0**

Contents

[List of Abbreviations 3](#_Toc109649785)

[1 Administrative Information 4](#_Toc109649786)

[1.1 Version History 4](#_Toc109649787)

[1.2 Title and Trial Registration 4](#_Toc109649788)

[2 Introduction and Study Aims 4](#_Toc109649789)

[3 Study Design 5](#_Toc109649790)

[3.1 Roles and Responsibilities 5](#_Toc109649791)

[3.2 Data Sources 5](#_Toc109649792)

[3.2.1. Patient-Reported Outcomes 5](#_Toc109649793)

[3.2.2 Linkage to the NHS Diabetic Eye Screening Programme Data 6](#_Toc109649794)

[3.2.3 Visual Function Questionnaire (NEI-VFQ-25) 7](#_Toc109649795)

[3.3 Analysis Populations 7](#_Toc109649796)

[3.3.1 Cohort A (ASCEND) 7](#_Toc109649797)

[3.3.2 Cohort B (DESP-Linked) 7](#_Toc109649798)

[3.3.3 Cohort C (VFQ-Responders) 8](#_Toc109649799)

[3.3.4 Consort Diagrams for the Analysis Cohorts 8](#_Toc109649800)

[3.4 Baseline Characteristics 8](#_Toc109649801)

[3.4.1 Baseline Characteristics of Cohort B Analysis Subsets 9](#_Toc109649802)

[3.4.2 Comparison of Participants Included and Excluded from Analysis Cohorts 10](#_Toc109649803)

[3.5 Definitions for Efficacy and Safety Outcomes 10](#_Toc109649804)

[3.5.1 General Principles 10](#_Toc109649805)

[3.5.2 Hypotheses 11](#_Toc109649806)

[3.5.3 Primary Efficacy Endpoint 11](#_Toc109649807)

[3.5.4 Primary Safety Endpoint 11](#_Toc109649808)

[3.5.5 Secondary Efficacy Endpoints 11](#_Toc109649809)

[3.5.6 Secondary Safety Endpoint 12](#_Toc109649810)

[3.5.7 Tertiary Endpoints 12](#_Toc109649811)

[3.5.8 Exploratory Assessments 13](#_Toc109649812)

[3.5.9 Power Calculations for the Primary and Secondary DESP Endpoints 13](#_Toc109649813)

[3.6 Statistical Methodology 14](#_Toc109649814)

[3.6.1 Definitions of Treatment Duration and Censoring Dates 14](#_Toc109649815)

[3.6.2 Definitions of Baseline 15](#_Toc109649816)

[3.6.3 Definition of the Final Eye Screening Record 15](#_Toc109649817)

[3.6.4 Units of Analysis for the DESP Data 15](#_Toc109649818)

[3.6.5 Study Average Compliance 15](#_Toc109649819)

[3.6.6 Completeness and Duration of Follow-up 16](#_Toc109649820)

[3.6.7 Baseline Characteristics 17](#_Toc109649821)

[3.6.8 Analyses of Time to First Event - Efficacy analyses involving cohorts B1-3 and B5, and the safety analyses involving cohort A and C 17](#_Toc109649822)

[3.6.9 Analyses of Ordinal Data 17](#_Toc109649823)

[3.6.10 Multiplicity 18](#_Toc109649824)

[3.6.11 Effect of Adjudication on Event Categorisation 18](#_Toc109649825)

[3.6.12 Sensitivity Analyses 18](#_Toc109649826)

[3.6.13 Tests for Heterogeneity and Trends in Effect 19](#_Toc109649827)

[3.6.14 Handling Missing Data 19](#_Toc109649828)

[3.6.15 Presentation of the Results 20](#_Toc109649829)

[Appendix 1: Calculation of NEI-VFQ-25 Scores 21](#_Toc109649830)

[Appendix 2: Summary of DESP Analysis Cohort Restrictions 23](#_Toc109649831)

[Appendix 3: Summary of Objectives, Outcomes, Data Sources and Analysis Populations 24](#_Toc109649832)

[Appendix 4: Visual Acuity 25](#_Toc109649833)

[Appendix 5: Other Eye Events 26](#_Toc109649834)

[Appendix 6: Post-Adjudication Read Code Categories 27](#_Toc109649835)

[References 28](#_Toc109649836)

# List of Abbreviations

| AE | Adverse event |
| --- | --- |
| AMD | Age-related macular degeneration |
| ASCEND | A Study of Cardiovascular Events iN Diabetes |
| BCVA | Best corrected visual acuity |
| DAP | Data Analysis Plan |
| DESP | Diabetic Eye Screening Programme |
| DMO | Diabetic macular oedema |
| DR | Diabetic retinopathy |
| ETDRS | Early Treatment Diabetic Retinopathy Study |
| FU | Follow-up |
| GP | General practitioner |
| HDL | High density lipoprotein |
| IQR | Interquartile range |
| LDL | Low density lipoprotein |
| NEI-VFQ-25 | National Eye Institute Visual Function Questionnaire-25* |
| NPDR | Non-proliferative diabetic retinopathy |
| NSC | National Screening Committee |
| PDR | Proliferative diabetic retinopathy |
| RMP | A reference made to a participant’s retinopathy grade, maculopathy status and photocoagulation status |
| SD | Standard deviation |
| SOP | Standard Operating Procedure |
| VA | Visual acuity |

*Note that to enhance the writing style, VFQ and NEI-VFQ-25 are used interchangeably

# 1 Administrative Information

## 1.1 Version History

| 1.0 | Initial version | Created by: Emily Sammons  Approved on: 25^th^ July 2022 |
| --- | --- | --- |

## 1.2 Title and Trial Registration

| Title: | ASCEND-Eye: A sub-study of the ASCEND randomised placebo-controlled trial, exploring the effect(s) of aspirin and omega-3 fatty acids on eye disease. |
| --- | --- |
| Eudract number: | 2004-000991-15 |
| MREC number: | North West- Haydock REC 03/08/087 |
| ClinicalTrials.gov number: | NCT00135226 |
| Sponsor: | Clinical Trial Service Unit & Epidemiological Studies Unit (CTSU), University of Oxford |
| Funder: | British Heart Foundation, Bayer, Macular Society |
| Protocol: | ASCEND-Eye is included in the ASCEND documentation: Protocol version 10.2_2018-07-23 |

# 2 Introduction and Study Aims

This Data Analysis Plan (DAP) describes the strategy, rationale and statistical methods that will guide assessment of the clinical efficacy and safety of aspirin and omega-3 fatty acids on eye disease in a sub-study of the ASCEND randomised, placebo-controlled trial, called ASCEND-Eye. Details of the ASCEND trial have been published elsewhere^1-4^. All analyses described in this DAP were pre-specified prior to unblinding of the ASCEND-Eye results, with the exception of sight-threatening eye bleeding events, which were included in the main ASCEND trial safety analyses. The latter were pre-specified prior to unblinding the ASCEND results^2^. The structure of this document conforms with guidelines for the contents of statistical analysis plans developed in collaboration with the network of UK Clinical Research Collaboration-registered trial units^5^.

The main aims of ASCEND-Eye are:

1. To determine whether aspirin or omega-3 fatty acids alter the course of diabetic retinopathy or diabetic maculopathy
2. To explore longitudinal differences in visual acuity between the study treatment arms
3. To determine the role of aspirin and separately, omega-3 fatty acids on incident diagnoses of age-related macular degeneration (AMD)
4. To compare differences between treatment arms in composite visual function scores derived from the National Eye Institute’s Visual Function Questionnaire-25 (NEI-VFQ-25)
5. To characterise the occurrence and severity of participant-reported eye bleeding events by treatment allocation
6. To identify the clinical and demographic characteristics that are associated with lower composite and subdomain scores on the NEI-VFQ-25, and to consider how concerns about eye health impact activities of daily living and emotional well-being*
7. To describe incident reports of other eye diseases between the treatment arms, such as, but not limited to, cataracts, glaucoma, retinal vein thrombosis, infections and ocular nerve palsies

In addition, exploratory assessments will be made of other possible beneficial or adverse effects of aspirin and omega-3 fatty acids during the scheduled treatment period among particular subgroups, based on data recorded at the randomisation visit.

*The observational analyses relevant to aim vi) are not described in this DAP.

# 3 Study Design

## 3.1 Roles and Responsibilities

All analyses for reports, presentations and publications will be prepared by the coordinating centre at the Clinical Trial Service Unit, University of Oxford (the regulatory sponsor of the ASCEND trial and its sub-studies).

## 3.2 Data Sources

Data on eye health was derived from the following three sources:

### 3.2.1. Patient-Reported Outcomes

Participants were able to report diagnoses of serious eye conditions on the routine ASCEND follow-up questionnaires they were sent every 6 months after randomisation. Prior to unblinding the ASCEND results the following events were adjudicated against supporting information provided by participants’ general practitioners, using pre-specified criteria:

- Bleeding in the eye^[[1]](#footnote-1)^
- Age-related macular degeneration
- Blindness
- Diabetic macular oedema
- Retinal detachment
- Visual disturbance

A further opportunity to report eye events was on the visual function questionnaire (VFQ) that was sent to a subset of eligible participants when ASCEND had concluded (see section 3.2.3 and 3.3.3). An additional page of questions was added to the standard NEI-VFQ-25 which asked participants to report the following types of event:

- Age-related macular degeneration
- Cataract
- Glaucoma
- Retinal vein thrombosis
- Other eye problems^[[2]](#footnote-2)^

Eye events that were reported on the VFQ were not subject to adjudication, unlike those reported on in-trial follow-up questionnaires in ASCEND. Allowance for the difference in event verification is made in the definitions of primary and secondary outcome (see section 3.5), and in the statistical methodology for treatment comparisons, that is described in Section 3.6.

#### 3.2.1.1 Coding of Adverse Events

All adverse events (AEs) were coded using the Read coding system, using software developed in-house.

### 3.2.2 Linkage to the NHS Diabetic Eye Screening Programme Data

Longitudinal information about ASCEND participants’ diabetic retinopathy and maculopathy grades, their photocoagulation status and best corrected visual acuity, was sought by electronic linkage to the NHS Diabetic Eye Screening Programme (DESP) data in England and Wales.

The National Screening Committee (NSC) for England and Wales grades retinal changes based on the clinical features shown in Table 1^6^. Reference is made to both the NSC grade and its corresponding international term elsewhere in the DAP.

Table 1 Retinal Grading System Recommended by the National Screening Committee for England and Wales

| **NSC Grade** | **International Term** | **Clinical Features** | **Treatment Pathway** |
| --- | --- | --- | --- |
| **Retinopathy (excluding the macula)** | | | |
| R0 | No DR | Normal retina | Annual rescreen |
| R1 | Mild NPDR (background DR) | Microaneurysms and intraretinal microvascular abnormalities (IRMAs) | Annual rescreen |
| R2 | Moderate-severe NPDR (pre-proliferative DR) | Extensive microaneurysms. Hard exudate, IRMAs, blot haemorrhages, cotton wool spots, venous beading | Refer to Ophthalmology |
| R3S | PDR (stable treated) | No haemorrhages, exudates or new vessels and evidence of treated disease | Annual rescreen |
| R3a | PDR (active) | Neovascularisation of the optic disc (NVD) or elsewhere (NVE), vitreous haemorrhage, glaucoma, retinal detachment | Refer to Ophthalmology |
| **Maculopathy** | | | |
| M0 | No DMO | No diabetic maculopathy | Annual rescreen |
| M1 | DMO | Macular oedema within 500µm of the foveal centre | Refer to Ophthalmology |
| **Photocoagulation status** | | | |
| P0 | No photocoagulation | Photocoagulation scars absent | - |
| P1 | Photocoagulation | Photocoagulation scars present | - |

DR = diabetic retinopathy; NPDR = non-proliferative diabetic retinopathy; PDR = proliferative diabetic retinopathy; DMO = diabetic macular oedema

Upon receipt of the DESP dataset, it became apparent that there was incomplete longitudinal coverage of screening results per participant, per year, per eye and/or per component of the screening assessment (i.e. R, M, and P grades and visual acuity score). Given the complex nature of these data, some blinded preliminary analyses were conducted, to determine the limits for inclusion in the DESP-linkage analyses and trial endpoints. The analysis plan presented reflects decisions that were made after this initial exploration of the data.

### 3.2.3 Visual Function Questionnaire (NEI-VFQ-25)

All surviving participants of ASCEND, who were on web- or mail-based follow-up at the end of the trial (31^st^ July 2017), were sent the National Eye Institute Visual Function Questionnaire-25 (NEI-VFQ-25)^7^. The NEI-VFQ-25 is a standardised and frequently-used questionnaire which was developed by the National Eye Institute in the US^7^. The NEI-VFQ-25 seeks information from participants about the effect of visual impairment on activities of daily living and emotional well-being, and from this a composite score and 11 sub-scale scores are derived: general vision, ocular pain, near vision, distance vision, social function, mental health, role limitations, dependency, driving, colour vision and peripheral vision.. Each score ranges from 0 to 100 with higher scores representing better vision-specific quality of life.

This multi-domain instrument has been shown to be a reliable and valid questionnaire across a range of chronic eye conditions^7^. Composite and subdomain scores derived from the questionnaire for near-vision and distance-vision have previously been shown to be strongly and independently correlated with visual acuity^7^. Although the latter is an objective measure of macular function, it may not reflect the full extent to which retinal diseases, such as diabetic retinopathy and age-related macular degeneration, affect other dimensions of visual function including contrast sensitivity, colour perception and stereoscopic vision^8^. Therefore the NEI-VFQ-25 has been recommended by regulators for use as a more holistic endpoint, and for vision-targeted cost-utility analyses in ocular intervention trials^9^.

Calculation of the NEI-VFQ-25 composite and sub-domain scores will follow the scoring algorithm prescribed by the instrument developers (see appendix 1)^7^.

## 3.3 Analysis Populations

The analyses described henceforth refer to three discrete patient populations, which reflect the three different sources of data collection described in section 3.2:

- Those involving the full cohort of randomised ASCEND participants (Cohort A)
- Those involving participants with linked data from the NHS Diabetic Eye Screening Programme (DESP) for the in-trial period of ASCEND (Cohort B)
- Those involving participants who returned a Visual Function Questionnaire (VFQ) (Cohort C)

### 3.3.1 Cohort A (ASCEND)

This cohort includes the entire population of randomised participants from the ASCEND trial and shall be relevant to analyses of safety, incident AMD events and other patient-reported eye outcomes.

### 3.3.2 Cohort B (DESP-Linked)

This cohort includes randomised participants from the ASCEND trial who attended eye screening appointments at a collaborating DESP. The following participants are excluded:

- Those who withdrew their consent or were lost to follow-up in the ASCEND trial
- Those who wished to opt out of the DESP linkage exercise
- Those who resided in Scotland or Northern Ireland
- Those served by a DESP that was unwilling or failed to respond to invitations to collaborate on ASCEND-Eye
- Those who attended a GP practice in England that did not register their data with Public Health England
- Those without an NHS number

Elsewhere in this document the linkage cohort is divided into cohorts B1-7 for specific analyses, as described in appendix 2.

### 3.3.3 Cohort C (VFQ-Responders)

This cohort includes randomised participants from the ASCEND trial who were eligible to receive the VFQ and who returned it. Those sent the VFQ included anyone who was alive and on web-, mail- or telephone-based follow-up at the end of ASCEND (31^st^ July 2017). It excludes the following individuals:

- Those who were known to be dead prior to beginning mailings of the VFQ on 20^th^ February 2018
- Those on GP- or central registry-follow-up at the end of ASCEND
- Those who withdrew their consent or were lost to follow-up at the end of ASCEND

### 3.3.4 Consort Diagrams for the Analysis Cohorts

The presentation of results for each of the three main analysis cohorts (A, B1, C) will begin with a consort diagram which summarises the number and percentage of participants who were:

- Valid for inclusion in the dataset used to estimate the primary outcomes and, where relevant, the secondary and tertiary outcomes (by reason)
- Excluded from the dataset used to estimate the primary outcomes and, where relevant, the secondary and tertiary outcomes (by reason)

Truncated consort diagrams showing those who were excluded from cohort B1 in DESP subsets B2-7, and the reason, will also be produced.

## 3.4 Baseline Characteristics

To assess the balance of important demographic and clinical variables between randomised treatment arms; the following baseline characteristics recorded at the time of randomisation into ASCEND will be presented for analysis cohorts A, B1 and C, overall and by randomised treatment allocation (Table 2):

Table 2 Baseline Characteristics to be tabulated

| **Characteristic** | **Stratification** | **Units** | **Summary statistics** |
| --- | --- | --- | --- |
| Age | <60 years  ≥60<70 years  ≥70 years | Years | Mean ± SD  Count (%) |
| Sex | Male  Female | - | Count (%) |
| Type of diabetes | Type 1  Type 2 | - | Count (%) |
| Duration of diabetes | 0 to <5 years  ≥5 <10 years  ≥10 to <20 years  ≥20 years | Years | Median  Inter-quartile range  Count (%) |
| Diabetes management | Diet-only  Oral hypoglycaemic agent(s) only  Insulin ± oral hypoglycaemic agent(s) | - | Count (%) |
| Patient-reported diabetic retinopathy | Yes  No  Unknown | - | Count (%) |
| Patient-reported hypertension | Yes  No  Unknown | - | Count (%) |
| Systolic blood pressure | <130  ≥130<140  ≥140  Not available | mmHg | Mean ± SD  Count (%) |
| Diastolic blood pressure | <75  ≥75<85  ≥85  Not available | mmHg | Mean ± SD  Count (%) |
| Body Mass Index | <25  ≥25<30  ≥30<35  ≥35  Not available | kg/m^2^ | Mean ± SD  Count (%) |
| Cigarette smoking | Current  Former  Never  Unknown | - | Count (%) |
| Non-study Medication^†^ | ACE inhibitor or ARB  Aspirin use before screening  Thiazide or related diuretic  Calcium channel blocker  Statin | - | Count (%) |
| Total cholesterol | <4.0  ≥4.0<5.0  ≥5.0  Not available | mmol/l | Mean ± SD  Count (%) |
| HDL-cholesterol | <1.0  ≥1.0<1.5  ≥1.5  Not available | mmol/l | Mean ± SD  Count (%) |
| Non-HDL cholesterol | <2.5  ≥2.5<3.5  ≥3.5  Not available | mmol/l | Mean ± SD  Count (%) |
| Glycosylated haemoglobin (HbA1c) | <6.5 (48)  ≥6.5 (48) <8.0 (64)  ≥8.0 (64)  Unavailable | DCCT(%) (IFCC (mmol/mol) | Mean±SD  Count (%) |
| CKD-EPI estimated GFR | ≥90  ≥60<90  <60  Not available | ml/min/1.73m^2^ | Mean±SD  Count (%) |
| Urinary albumin:creatinine ratio | <3  ≥3  Not available | mg/mmol | Median  Count (%) |
| Townsend Deprivation Index* | <-3  ≥-3<0  ≥0<2  ≥2<4  ≥4<6  ≥6  Not available | - | Count (%) |
| Ethnic origin | White  Indian/Pakistani/Bangladeshi  African/Caribbean  Other/unknown | - | Count (%) |

^†^Concomitant medications will be coded and grouped by class in the same way they were in the ASCEND trial^2^.

*The Townsend Deprivation Index derives from four key variables in census data: households without a car, overcrowded households, households not owner-occupied and persons unemployed. A score of 0 represents an area with a UK average value, positive values indicate relative deprivation and negative values indicate relative affluence.

### 3.4.1 Baseline Characteristics of Cohort B Analysis Subsets

To compare differences between the baseline characteristics of B1 and its analysis subsets (B2-7), the following key determinants of diabetic retinopathy progression, recorded at the time of randomisation into ASCEND will be tabulated for cohorts B1-7 overall (Table 3). To compare the balance between treatment arms, the same truncated baseline characteristics are to be tabulated for cohorts B2-7 overall.

Table 3 Truncated Baseline Characteristics Table for Cohort B Analysis Subsets

| **Characteristic** | **Stratification (where relevant)** | **Units** | **Summary statistics** |
| --- | --- | --- | --- |
| Age | <65 years  ≥65<70 years  ≥70 years | Years | Mean ± SD  Count (%) |
| Sex | Male  Female | - | Count (%) |
| Type of diabetes | Type 1  Type 2 | - | Count (%) |
| Duration of diabetes |  | Years | Median  Inter-quartile range |
| Systolic blood pressure |  | mmHg | Mean±SD |
| Diastolic blood pressure |  | mmHg | Mean±SD |
| Glycosylated haemoglobin (HbA1c) |  | DCCT(%) (IFCC (mmol/mol) | Mean±SD |
| CKD-EPI estimated GFR |  | ml/min/1.73m^2^ | Mean±SD |
| Urinary albumin:creatinine ratio |  | mg/mmol | Median |
| Ethnic origin | White | - | Count (%) |

### 3.4.2 Comparison of Participants Included and Excluded from Analysis Cohorts

Analysis cohorts B1-7 (DESP-linkage) and C (VFQ) represent subsets of the full ASCEND population (cohort A). The baseline characteristics of those excluded from analysis cohorts B1 and C will be presented overall, without stratification for treatment allocation, in order to assess differences with the full ASCEND population. Formal testing for heterogeneity between the baseline characteristics of those included in and excluded from analysis cohorts B1 and C will use the Chi-squared test for categorical variables, the Mantel-Haenszel Chi-square test for trend for ordinal variables (e.g. Townsend Deprivation Index), and the t-test or Wilcoxon-Mann-Whitney U test for continuous variables with a normal (summarised by mean±SD) or non-normal (summarised by median and inter-quartile range) distribution respectively.

## 3.5 Definitions for Efficacy and Safety Outcomes

### 3.5.1 General Principles

All comparisons of the main efficacy outcomes will be between those individuals allocated aspirin daily versus matching placebo, and separately, omega-3 fatty acid supplement daily versus matching placebo, during the scheduled treatment period. The factorial design of ASCEND should not have an effect on the statistical sensitivity with which the effects of each treatment arm can be assessed^10,11^. Comparisons of the aspirin arm will therefore be made without stratification by omega-3 fatty acid allocation (and vice versa for omega-3 fatty acid analyses).

Every randomised participant will be compared, regardless of whether they took all, some or none of their allocated treatment (i.e. intention-to-treat analyses). A participant may contribute to more than one assessment if they have more than one type of event. All data will be reviewed, blind to treatment allocation, by coordinating centre clinical staff and where relevant, coded in accordance with pre-specified criteria.

### 3.5.2 Hypotheses

For all statistical tests, the null hypothesis will be that allocation to aspirin and, separately, allocation to omega-3 fatty acids, on each outcome of interest, is the same as the effect of allocation to placebo. Therefore, the alternative hypothesis is that there is a difference in effect by treatment allocation.

Objectives and outcomes, and the relevant source of the following pre-specified data are summarised in Appendix 3.

### 3.5.3 Primary Efficacy Endpoint

The primary assessment will include a comparison of the effects of allocation to aspirin versus placebo, and separately, omega-3 fatty acid supplement versus placebo, on:

- **Referable disease** during the scheduled treatment period, where referable disease is defined as the composite of referable diabetic retinopathy (R_2_ or R_3a/s_) or referable diabetic maculopathy (M_1_), in either eye, among all those with in-trial retinopathy data (*Analysis cohort B1*)

### 3.5.4 Primary Safety Endpoint

It is anticipated that aspirin may increase the risk of sight-threatening eye-bleeding. Therefore, the primary safety assessment will include a comparison of the effects of allocation to aspirin versus placebo, on:

- **Confirmed sight-threatening eye bleeds** in either eye (*Analysis cohort A*)

### 3.5.5 Secondary Efficacy Endpoints

The secondary assessments will include a comparison of the effects of allocation to aspirin versus placebo, and separately, omega-3 fatty acid supplement versus placebo, on:

- **Confirmed or unrefuted incident diagnoses of AMD** (*Analysis cohorts A and C*)
- **Referable disease** (as defined above) during the scheduled treatment period, restricted to those with both retinopathy and maculopathy data, for both eyes at baseline and at the final eye screening record, who are either without disease (R_0_) or with only background diabetic retinopathy (R_1_), and no maculopathy (M_0_), in both eyes at baseline. *(Analysis cohort B2)*

To be analysed within the following strata of baseline retinopathy, and as an overall stratified analysis:

- - R_0_/R_0_
  - R_0_/R_1_ or R_1_/R_0_
  - R_1_/R_1_
- **Progression in retinopathy grade** in either eye during the scheduled treatment period, where progression is defined as an increase by 1 step or more in R grade, based on the NSC scoring protocol for retinopathy, excluding those with proliferative disease in both eyes on their baseline record (i.e. R3_a/s_/R3 _a/s_), and those with data in one eye only at baseline and/or the final eye screening record. (*Analysis cohort B3)*

To be analysed within the following strata of baseline retinopathy, and as an overall stratified analysis:

- 1. R_0_/R_0_
  2. R_0_/R_1_ or R_1_/R_0_
  3. R_1_/R_1_
  4. R_2_/R_1_ or R_1_/R_2,_ R_2_/R_2_, or R_2_/R_3_ or R_3_/R_2_

No attempt shall be made to distinguish the eye which progresses, in those with a different R grade in each eye on their baseline record.

- **Composite NEI-VFQ-25 score** (*Analysis cohort C*)

### 3.5.6 Secondary Safety Endpoint

The secondary safety assessment will include a comparison of the effects of allocation to aspirin versus placebo, on:

- **Any eye bleed**, overall and subdivided by severity: (*Analysis cohorts A and C*)
  - Sight-threatening eye bleeds (*Analysis cohort A*)
  - Non-serious eye bleeds (*Analysis cohort A*)
  - Unrefuted eye bleeds (*Analysis cohorts A and C*)

### 3.5.7 Tertiary Endpoints

Tertiary analyses are planned to assess the effects of allocation to aspirin versus placebo, and separately, omega-3 fatty acid supplement versus placebo, on:

- **Incident reports of diabetic macular oedema**, overall and subdivided by the source of this information^[[3]](#footnote-3)^:
  - Those events that were either confirmed or unrefuted from the 6-monthly follow-up questionnaires in ASCEND (*Analysis cohort A)*
  - Those with referable maculopathy (M_1_) in either eye among those without maculopathy (M_0_) in both eyes at baseline *(Analysis cohort B2)*
  - Those events that were reported on the visual function questionnaire (*Analysis cohort C*)
- **Final retinopathy grade** on the final eye screening record, in the following 4 combinations: R_0_/R_0_, R_1_/R_0_ or R_o_/R_1_, R_1_/R_1_, ≥R_2_/≥R_0_ or ≥R_0_/≥R_2_^[[4]](#footnote-4)^. This analysis will exclude those with data in neither eye or one eye only at their final eye screening record, and those whose final retinopathy data on each eye was more than one year apart (*Analysis cohort B4*)
- **Worsening of visual acuity** in either eye during the scheduled treatment period, compared to baseline, defined as an increase of an ASCEND-Eye Recode Score^[[5]](#footnote-5)^ of at least 3 points^[[6]](#footnote-6)^(*Analysis cohort B5*)
- The primary efficacy assessment by baseline characteristic (as listed in section 3.4 of this document)(*Analysis cohort B1*)
- 11 vision-targeted sub-domain scores from the NEI-VFQ-25 (*Analysis cohort C*)

### 3.5.8 Exploratory Assessments

Summary figures for incident reports of other patient-reported eye diseases will be presented, overall and by randomised treatment allocation. Specific event Read codes to be included in these analyses are listed in appendix 5.

In addition to the pre-specified comparisons, other analyses may be performed with due allowance for their data-dependent nature, including tabulations demonstrating the completeness of longitudinal DESP data and correlations between this and the number of endpoints.

### 3.5.9 Power Calculations for the Primary and Secondary DESP Endpoints

The following power calculations were based on the non-significant 23% risk reduction for development of proliferative retinopathy (R_3_), seen in a pre-specified subset of those with the least severe retinopathy (Level ≤35; n=609), who were assigned to aspirin in the ETDRS trial^12^. The power is shown in 5% increments up to this risk reduction. There is no precedent for omega-3 fatty acids on which separate power calculations could be based.

The formula used was for comparisons of survival curves between two groups under the Cox proportional-hazards model^13^. Assuming a 10% difference, estimates for the number of events in arm treatment arm were derived from the formula: b=N/2.1 and a = b x 1.1, where N = no. of events, a = placebo and b = active.

#### 3.5.9.1 Primary Efficacy Endpoint (Cohort B1)

Blinded preliminary analyses identified 1061 participants in cohort B1 who experienced a primary efficacy event. Based on this, there would be 75% to 95% power to detect 15% to 20% proportional reductions in the incidence of referable diabetic eye disease at 2p<0.05 (**Table 4**).

**Table 4 Power of ASCEND-Eye to detect different effects of the interventions on referable disease among the cohort of 7360 participants with in-trial retinopathy data**

| **Change in risk** | **N with event** | | **Power at 2P = 0.05** | **Power at 2P = 0.01** |
| --- | --- | --- | --- | --- |
|  | **Active**  **(N approx. 3680)** | **Placebo**  **(N approx. 3680)** |  |  |
| 25% | 471 | 589 | >99% | 98% |
| 20% | 482 | 578 | 95% | 85% |
| 15% | 493 | 567 | 75% | 53% |
| 10% | 505 | 556 | 40% | 20% |

#### 3.5.9.2 Secondary Efficacy Endpoint - Referable Disease (Cohort B2)

Based on blinded preliminary analyses, in which there were 240 events overall, there would be 24% to 41% power to detect 15% to 20% proportional reductions in the incidence of referable diabetic eye disease at 2p<0.05 (Table 5).

Table 5 Power of the study to detect different effects of the interventions on referable disease among the cohort of 2558 participants with in-trial retinopathy data and a baseline eye screening record

| **Change in risk** | **N with event** | | **Power at 2P = 0.05** | **Power at 2P = 0.01** |
| --- | --- | --- | --- | --- |
|  | **Active**  **(N approx. 1279)** | **Placebo**  **N approx. 1279)** |  |  |
| 25% | 107 | 134 | 60% | 36% |
| 20% | 109 | 131 | 41% | 20% |
| 15% | 112 | 129 | 24% | 9% |
| 10% | 114 | 125 | 13% | 4% |

#### 3.5.9.3 Secondary Efficacy Endpoint – Retinopathy Progression (Cohort B3)

Based on blinded preliminary analyses, in which there were 1249 events overall among the cohort of 2852 participants with in-trial and baseline retinopathy data, there would be 82% to 98% power to detect 15% to 20% proportional reductions in the incidence of retinopathy progression at 2p<0.05 (Table 6).

Table 6 Power of the study to detect different effects of the interventions on retinopathy progression among the cohort of 2852 participants

| **Change in risk** | **N with event** | | **Power at 2P = 0.05** | **Power at 2P = 0.01** |
| --- | --- | --- | --- | --- |
|  | **Active**  **(N approx. 1426)** | **Placebo**  **(N approx. 1426)** |  |  |
| 25% | 555 | 694 | >99% | 99% |
| 20% | 568 | 682 | 98% | 91% |
| 15% | 581 | 668 | 82% | 61% |
| 10% | 595 | 655 | 46% | 24% |

## 3.6 Statistical Methodology

### 3.6.1 Definitions of Treatment Duration and Censoring Dates

The duration of the scheduled treatment period will be defined as day 1 post-randomisation up to and including the censoring date in ASCEND^2^. The latter was the earliest of:

- An unrefuted death report (i.e. an adjudicated fatal serious adverse event)
- A final follow-up conducted by mail-based or electronic questionnaire, telephone to the participant, their relative or carer, or GP follow-up
- For anyone without a final follow-up via one of the methods listed above, until the end of the final follow-up window: 31^st^ March 2017*; or
- The date of withdrawal of consent to direct or third party follow-up

*The main method of follow-up of these individuals was through a central registry. Participants were considered to be on central registry follow-up if:

- Their last known postcode was in England or Wales on 31^st^ March 2017
- They were not known to have moved abroad before 31^st^ March 2017
- They had not withdrawn consent to follow-up

For those analyses involving cohort A, the censoring date will be the same as that which applied in the main ASCEND trial. For analyses involving cohort B, participants will be censored at the date of the final eye screening record with retinopathy data in at least one eye before the end of the scheduled treatment period.

### 3.6.2 Definitions of Baseline

For analysis cohorts A, B1 and C, the baseline is defined as the date of randomisation in ASCEND.

Relevant to the secondary DESP efficacy analyses (cohorts B2 and B3), the baseline record is defined as the eye screening assessment closest to randomisation in each eye that is on the date of randomisation or up to 2 years before randomisation. The majority of individuals had retinopathy data in both eyes on the same baseline record. If retinopathy data is missing for one eye in the baseline record closest to randomisation, an earlier record may be used for the missing eye, so long as both records occurred in the two years prior to randomisation. To simplify the narrative, both scenarios shall be referred to as the “baseline record”, irrespective of whether the data derived from eye screening assessments on different dates for different eyes. For all of the DESP analyses, pre-baseline and post-trial records will be ignored.

For all analysis cohorts, the demographic and clinical information recorded by participants on their randomisation questionnaire or blood sample, constituted their baseline characteristic data.

### 3.6.3 Definition of the Final Eye Screening Record

For cohort B, the final eye screening record is defined as the final post-randomisation eye screening record with retinopathy data in at least one eye before the end of the scheduled treatment period. The majority of individuals had retinopathy data in both eyes on the same final record. If retinopathy is missing for one eye on the final record, an earlier post-randomisation record will be used as the final grade in the missing eye. To simplify the narrative, both scenarios shall be referred to as the “final record”, irrespective of whether the last available post-randomisation retinopathy data came from screening assessments on different dates for different eyes.

### 3.6.4 Units of Analysis for the DESP Data

Diabetic retinopathy is a bilateral disease in which lesions presenting in the left and right eye are usually correlated, whereas most statistical methods assume independence of the sampling units^14,15^. Some ophthalmology trials mitigate this by measuring outcomes in only one eye, but this can be a cause of selection bias, or result in a loss of statistical power^15^. Therefore, the unit of analysis for all endpoints that involved the DESP data was to be the participant, however, data from either eye could contribute once in the time-to-first event analyses (B1-3, B5); a summary measure of the participant-dependent retinopathy grade combination in both eyes is to be used for those analyses involving cohort B4.

Within the endpoint descriptions and all results tables, R_x_/R_y_ denotes the combination of retinopathy grades in both eyes per participant; however, the ordering of these scores does not attempt to distinguish between the right and left eye.

### 3.6.5 Study Average Compliance

Study average compliance will be considered when interpreting all pre-specified analyses. ASCEND-Eye will observe the same rules regarding analyses of study average compliance as those defined in the main ASCEND trial DAP^2^. Here it is stated “participants at risk of the event in question are considered to be definitely or probably compliant if a follow-up form was received that indicates the participant took their treatment “every day” or “most days” during the follow-up period. Participants were also considered compliant if they had previously been compliant, were still receiving medication and had not reported stopping treatment, and information was received within the previous 7 months.”

Study-average reported definite or probable compliance with study treatment shall be reported for each of the three main analysis populations (A, B1, C), in each treatment arm by randomised allocation, and by:

- Time post-randomisation in the following intervals and overall: <3, ≥3<5, ≥5<7, ≥7 years.
- Baseline characteristics (see section 3.4)

A summary study average reported definite or probable compliance shall be reported for cohorts B2-7, overall and by randomized treatment allocation, but without stratifying by 3 year intervals or the baseline characteristics.

In the main ASCEND analyses, an average compliance weighted by person-years at risk of the primary outcome was estimated, because there was an expectation that following a vascular event, participants would stop their allocation in the aspirin arm in order to take prescribed aspirin. In ASCEND-Eye, compliance estimates will only be based on person-years in the study, i.e. with censoring for death or the end of the scheduled treatment period, because neither study medication is likely to be prescribed by third parties following the eye events. Hence, the compliance estimates presented for cohort A will differ slightly from those included in the ASCEND Clinical Study Report and publications.

#### 3.6.5.1 Use of Non-Study Antiplatelet or Anticoagulant Therapy

The use of non-study antiplatelet or anticoagulant therapy shall be reported for analysis populations A, B1 and C, by randomised allocation to aspirin versus placebo, overall, and by the number of years post-randomisation in the following intervals: <3, ≥3<5, ≥5<7, ≥7 years.

#### 3.6.5.2 Reasons for Stopping Treatment

Reasons for stopping treatment shall be reported for analysis populations A, B1 and C, by randomised allocation to aspirin versus placebo and, separately, omega-3 versus placebo, and by:

- Serious adverse event (by type and overall)
- Non-serious adverse event (by type and overall)
- Other reasons (by type and overall)
- Total stopped for any reason

### 3.6.6 Completeness and Duration of Follow-up

ASCEND-Eye will observe the same rules regarding analyses of the completeness of follow-up as those defined in the main ASCEND trial data analysis plan^2^.

Completeness of follow-up shall be reported for analysis population A, in separate tables for each treatment arm, by randomised allocation, and by the proportion of those with:

- Complete follow-up information – overall, and in the following categories:
  - Final follow-up visit completed by the participant or a carer
  - Final follow-up completed by the participant’s GP
  - Final follow-up completed by linkage to a central registry.
  - Died (follow-up prior to morbidity complete)
- Incomplete follow-up information – overall, and in the following categories:
  - Consent withdrawn
  - Moved abroad
  - Died (follow-up prior to morbidity incomplete)
  - No final follow-up information

Duration of follow-up shall be reported for analysis population A in each treatment arm by randomised allocation, and by:

- Mean length of follow-up (SD; years)
- Median length of follow-up (IQR; years)
- Person-years of follow-up overall, and by follow-up method
  - Participant
  - General practitioner
  - Registry linkage-only
  - Died with complete follow-up
  - Incomplete follow-up information

Only the mean and median length of follow-up, in each treatment arm and by randomized allocation will be presented for cohorts B1-7 and C

### 3.6.7 Baseline Characteristics

Univariate analyses will be used to describe baseline characteristics for all three analysis cohorts. These will be presented as counts (percentage) for categorical variables, as mean (standard deviation; SD) for normally distributed continuous variables, and as median (interquartile range; IQR) for non-normally distributed continuous variables.

### 3.6.8 Analyses of Time to First Event - Efficacy analyses involving cohorts B1-3 and B5, and the safety analyses involving cohort A and C

Logrank^11,16^ and stratified logrank methods^17^ will be used to estimate the average event rate ratio, 95% confidence intervals and two-sided p-values, in those allocated active aspirin vs. placebo, and separately, omega-3 fatty acids *vs.* placebo. For the primary safety assessments, all analyses will be based on confirmed incident sight-threatening eye bleed diagnoses, defined as any relevant patient-reported outcome, verified at the time of adjudication by medical record evidence of the diagnosis, and subsequent to the date of randomisation. It will exclude events for which no supporting documentation was received from the participants’ GP and those that derive from the VFQ which were not eligible to be adjudicated (i.e. unrefuted events). The secondary safety analyses will include both confirmed and unrefuted events derived from ASCEND’s 6-monthly follow-up questionnaires and the VFQ.

### 3.6.9 Analyses of Ordinal Data

#### 3.6.9.1 Tertiary Analysis of Final Retinopathy Grade (Cohort B4)

To compare the net severity of retinopathy grades across both eyes between treatment arms, a proportional odds logistic regression model^18,19^ will be fitted to duplex final retinopathy grades grouped into a 5-point ordinal scale as the outcome variable: R_0_/R_0_, R_1_/R_0_ or R_0_/R_1_, R_1_/R_1_, ≥R_2_/≥R_0_ or ≥R_0_/≥R_2_, ≥R_2_/≥R_2_. A common odds ratio with 95% confidence intervals will be used to estimate the average effect size over the total ordinal scale, caused by allocation to aspirin versus placebo and, separately, omega-3 fatty acids versus placebo.

#### .3.6.9.2 Secondary Efficacy and Tertiary Analyses of Dependent Variables from the VFQ (Cohort C)

The calculation of NEI-VFQ-25 subdomain and composite score will be performed using the “NEI-VFQ-25 Scoring Algorithm – August 2000”^7^ (see appendix 1). The composite and 11 subdomain scores deriving from the VFQ will be summarised descriptively for each treatment arm. A proportional odds logistic regression model^18,19^ will be fitted to the composite scores grouped into a 5-point ordinal scale as the outcome variable: ≥90, 80-89, 70-79, 60-69 and <60. These categories were chosen because preliminary, blinded analyses confirmed that the distribution of mean composite scores was negatively skewed. A common odds ratio with 95% confidence intervals will be used to interpret the effect size of allocation to aspirin versus placebo and, separately, omega-3 fatty acids versus placebo. The same approach will be used to analyse the 11 subdomains. A proportional odds logistic regression model will be fitted to subdomain scores grouped into ordinal scales, shown table 7, as the outcome variable. Groupings were based on a blinded preliminary assessment of the subdomain score distributions.

**Table 7: Composite and vision-targeted subdomain scoring categories**

| **VFQ Component** | **Ordinal scale** |
| --- | --- |
| **Composite score** | ≥90, 80-89, 70-79, 60-69 and <60 |
| **Subdomain score** |  |
| General Vision | ≥100,80-99,60-79 and <60 |
| Ocular Pain | ≥90,80-89, 70-79,60-69 and <60 |
| Near Activities | 100, 90-99, 80-89, 70-79 and <70 |
| Distance Activities | 100, 90-99, 80-89, 70-79 and <70 |
| Social Functioning | ≥90, 80-89, 70-79 and <70 |
| Mental Health | ≥90, 80-89, 70-79, 60-69 and <60 |
| Role Dependency | ≥90, 80-89, 70-79 and <70 |
| Vision-Specific Dependency | 100, <100 |
| Driving | 100, 80-99, 60-79 and <60 |
| Colour Vision | 100,≥75<100 and <75 |
| Peripheral Vision | 100,≥75<100 and <75 |

#### 3.6.9.3 Exploratory Analyses of Other Eye Events

First occurrences of other eye events listed in appendix 5 will be summarised descriptively for each treatment arm. No inferential testing of these is planned.

### 3.6.10 Multiplicity

The testing of multiple hypotheses simultaneously, increases the likelihood of a result being due to chance alone^10,11^. For the primary efficacy and safety analyses, two-sided p-values (2P) <0.05 will be considered statistically significant. No adjustment for multiplicity shall be made for the secondary or tertiary analyses. The results from these analyses will be interpreted with due caution, and in the context of existing studies. A true effect of treatment allocation will be considered to be more likely if the comparisons are based on a large number of events, and if the upper and lower confidence intervals are further away from zero (which would be associated with a more extreme p-value).

### 3.6.11 Effect of Adjudication on Event Categorisation

To interpret the impact of adjudication on secondary efficacy, tertiary and safety outcomes, a contingency table shall be produced which shows the pre-adjudicated and post-adjudication outcome categorisation of the following events:

- Any eye bleed
- Retinal detachment
- Macular oedema
- Age-related macular degeneration
- Blindness
- Visual deterioration

Read codes relevant to the post-adjudication categorisation of each of these events are presented in appendix 6.

### 3.6.12 Sensitivity Analyses

#### 3.6.12.1 Ordinal Logistic Regression of Final Retinopathy Grades in those with Baseline Eye Screening Records

A sensitivity analysis is planned to assess whether restricting comparisons of final retinopathy grade to those with a baseline record, impacts the results of this tertiary endpoint (cohort B6; a subset of cohort B4). As described in section 3.6.9.1, a proportional odds logistic regression model^18,19^, will be applied to duplex final retinopathy grades grouped into a 5-point ordinal scale and a common odds ratio with 95% confidence interval will be used to estimate the average effect size over the total ordinal scale, caused by allocation to aspirin versus placebo and, separately, omega-3 fatty acids versus placebo.

#### 3.6.12.2 Ordinal Logistic Regression of the Highest Retinopathy Grade Reached

A sensitivity analysis is planned to assess whether analyses of the highest retinopathy grade reached, impacts the results of the tertiary endpoint analysis of final retinopathy grades. As described in section 3.6.9.1, a proportional odds logistic regression model^18,19^, will be applied to duplex final retinopathy grades grouped into a 5-point ordinal scale and a common odds ratio with 95% confidence interval will be used to estimate the average effect size over the total ordinal scale, caused by allocation to aspirin versus placebo and, separately, omega-3 fatty acids versus placebo. This analysis will be restricted to those with retinopathy data in both eyes post-randomisation (cohort B7).

Tabulations demonstrating the completeness of longitudinal DESP data and correlations between this and the number of screening assessments and endpoint events will be produced in conjunction with these analyses.

### 3.6.13 Tests for Heterogeneity and Trends in Effect

A limited number of AMD are expected in the ASCEND-Eye study due to the relatively young age of participants in ASCEND (mean 63.3; SD 9.2)^2^. Hence chance alone may result in differences in the treatment effect size on particular outcomes if comparisons are made in smaller subgroups^10,11,20^. Therefore, heterogeneity of the proportional effect of the ASCEND medications on AMD events, in subgroups of baseline characteristics will not be formally tested^21^. However, event rates will be summarised descriptively, for each treatment arm, in the following categories: (*Analysis cohorts A and C)*

- - Age: <65 years, ≥65<70 years and ≥70 years
  - Sex: male, female
  - Smoking status: current, former, never, unknown

### 3.6.14 Handling Missing Data

The following rules will be applied to categorise participants into subgroups of the baseline characteristics where the information about that variable is not known:

1. Where a substantial number of participants have missing or unknown information on the variable, a subgroup of “Unknown” should be analysed separately.
2. Where only a small number of participants have missing information, those with missing information should be counted in the median category for continuous variables and in the modal group for categorical variables.

The same rules were applied in the ASCEND trial analyses^2^.

#### 3.6.14.1 Partial Dates

The same rules regarding partial dates that were applied to the main ASCEND study^2^, shall be applied to the ASCEND-Eye study:

- Where there is incomplete information for the day, for both serious and non-serious adverse events, the day should be assigned as the 15^th^ of the month unless the month and year were the same as the month and year of randomisation, in which case, the mid-point between the date of randomisation and end of the month was to be used.
- Where there is incomplete information for the day and month, the missing day will be imputed as described above and the missing month will be imputed as June. Next, replacement data will be used according to the worst-case scenario:
  - In the case of missing data from a safety variable (e.g. eye bleeding events), if the replaced start date is prior to randomisation, the start date will be set to the date of randomisation (i.e. it will be considered to be a post-randomisation event)
  - In the case of missing data for efficacy outcomes, if the replaced date is prior to randomisation, the start date will be set to the day before randomisation (i.e. it will be considered to be a pre-randomisation event)
- Where information is missing for the day, month and year replacement data will be used according to the worst-case scenario as described above.

Where the same type of event has been reported during the main ASCEND trial follow-up and on the NEI-VFQ-25 form, the ‘first event’ used for the analyses shall be the earliest occurrence that has been confirmed by adjudication. If an event reported during the main ASCEND trial did not undergo adjudication, the first event shall be regarded as the earliest occurrence reported by either source.

### 3.6.15 Presentation of the Results

A conventional approach of rounding numbers ending in 0-4 down and numbers ending 5-9 up will be used to present the ASCEND-Eye results. Rounding will occur as the last step in producing tables and figures; it shall not be used in intermediate calculations.

The formatting of p-values will follow the New England Journal of Medicine guidelines, which are:

- Where p>0.01, show 2 decimal places
- Where p ≤0.01 and p ≥0.001, show 3 decimal places
- Where p <0.001, display “<0.001”

# Appendix 1: Calculation of NEI-VFQ-25 Scores

The calculation of NEI-VFQ-25 sub-scales and total score will be performed using the “NEI-VFQ-25 Scoring Algorithm – August 2000”^7^.The NEI-VFQ-25 consists of 25 questions representing 11 vision-targeted sub-scales and an additional single general health rating question. Scoring of the VFQ is a two-step process:

1. Step 1: Numeric values from the survey are recoded following the scoring rules shown in table A. All items are scored so that a high score represents better functioning. In this format, scores represent the achieved percentage of the possible total, e.g. a score of 25 represents 25% of the highest possible score.
2. Step 2: Items within each subscale are averaged together. Table B indicates which items contribute to each sub-domain.

To calculate an overall composite score, the sub-scale scores are averaged, excluding the general health rating question (Q1). By averaging sub-scale scores rather than individual items, equal weight is given to each visual domain, whereas averaging the items would give more weight to scales with more items.

| **Table A: Recoding of responses (analysis step 1)** | |  |
| --- | --- | --- |
| **Question Number** | **Change response category** | **To recoded value of** |
| 1,3,4,15c | 1 | 100 |
|  | 2 | 75 |
|  | 3 | 50 |
|  | 4 | 25 |
|  | 5 | 0 |
| 2 | 1 | 100 |
|  | 2 | 80 |
|  | 3 | 60 |
|  | 4 | 40 |
|  | 5 | 20 |
|  | 6 | 0 |
| 5,6,7,8,9,10,11, 12, 13, 14, 16, 16a, | 1 | 100 |
|  | 2 | 75 |
|  | 3 | 50 |
|  | 4 | 25 |
|  | 5 | 0 |
|  | 6 | * |
| 17, 18, 19, 20, 21, 22, 23, 24, 25 | 1 | 0 |
|  | 2 | 25 |
|  | 3 | 50 |
|  | 4 | 75 |
|  | 5 | 100 |
|  |  |  |

*Response choice “6” indicates that the person does not perform the activity because of non-vision related problems. If this choice is selected, the item is coded as missing.

**Data analysis rules to guide analyses of responses to the driving questions**

Questions 15c, 16 and 16a are the only questions included in the driving subdomain, however participants navigate to Q15c via three screening questions which are intended to clarify whether they are currently driving or not. Where there is missing data or where a nonsensical combination of answers has been given for questions 15 to 16a, ASCEND-Eye will apply the following data analysis rules:

In analyses of Q15c, include those who gave positive responses to both Q15 and Q15c plus negative responses to both Q15a and Q15b. Q15c has 4 response levels, but this is expanded to a 5-level response using Q15b. According to the NEI-VFQ-25 scoring algorithm:

- If 15b = 1, then 15c should be recoded to 0
- If 15b = 2, then 15c should be recoded to missing
- If 15b = 3, then 15c should be recoded to missing.

Questions 16 and 16a may be answered by anyone who has ever driven and therefore includes those who are currently driving (as in question 15), and those who have previously driven but gave up (question 15a). Questions 16 and 16a offer a second opportunity to indicate that an individual has stopped driving at night or in adverse weather conditions respectively, due to visual impairment or other reasons. Therefore, individuals should be included in analyses of questions 16 and 16a if an answer has been given, irrespective of their responses to questions 15, 15b or 15c, but should exclude those who have never driven in response to question 15a.

**Averaging of questions to generate NEI-VFQ-25 sub-scales**

| **Table B: average scores within each subscale** | |  |
| --- | --- | --- |
| **Scale** | **Number of questions** | **Questions to be averaged (after recoding)** |
| General health | 1 | 1 |
| General vision | 1 | 2 |
| Ocular pain | 2 | 4, 19 |
| Near activities | 3 | 5, 6, 7 |
| Distance activities | 3 | 8, 9. 14 |
| Vision-specific: social functioning | 2 | 11,13 |
| Vision-specific: mental health | 4 | 3, 21, 22, 25 |
| Vision-specific: role difficulties | 2 | 17, 18 |
| Vision-specific: dependency | 3 | 20, 23, 24 |
| Driving | 3 | 15c, 16, 16a |
| Colour vision | 1 | 12 |
| Peripheral vision | 1 | 10 |
|  |  |  |

**Formula:**

Mean = (score for each item with a non-missing answer)/total no. of items with non-missing answers

Note: 100 = best possible score, 0 = worst possible score.

Per Analysis algorithm provided by NEI - items that are left blank (missing data), are not taken into account when calculating the scale scores. Sub-scales with at least one item answered can be used to generate a sub-scale score. Hence, scores represent the average for all items in the subscale that the respondent answered.

# Appendix 2: Summary of DESP Analysis Cohort Restrictions

|  | **Restrictions** | | **Summary of exclusions*** |
| --- | --- | --- | --- |
|  | **Final visit**** | **Baseline***** |  |
| **Primary Efficacy Endpoint Analysis – Any referable disease (cohort B1)** | Not relevant | Not relevant | Those without any retinopathy data during the scheduled treatment period |
| **Secondary Efficacy Endpoint Analysis – Referable disease in those without referable disease at baseline and**  **Tertiary Analysis – Referable maculopathy (cohort B2)** | Those with both retinopathy and maculopathy data in both eyes | Those with both retinopathy and maculopathy data in both eyes | Those with retinopathy data in neither eye or one eye only at baseline and/or the final record  Those with maculopathy data in neither eye or one eye only at baseline and/or the final record  Those with referable disease at baseline in either eye (R_2_, R_3_ and/or M_1_) |
| **Secondary Efficacy Analysis – Any retinopathy progression**  **(cohort B3)** | Those with retinopathy data in both eyes | Those with retinopathy data in both eyes | Those with retinopathy data in neither eye or one eye only at baseline and/or the final record  Those with proliferative disease in both eyes at baseline (i.e. R_3a/s_/R_3 a/s_) |
| **Tertiary Analysis – Final retinopathy grade (cohort B4)** | Those with retinopathy data in both eyes (if on different dates then within a year of each other) | Not relevant | Those with retinopathy data in neither eye or one eye only at the final record  Those with final retinopathy data on each eye more than 1 year apart |
| **Tertiary Analysis – loss of visual acuity (cohort B5)** | Those with visual acuity data in both eyes | Those with visual acuity data in both eyes | Those with visual acuity data in neither eye or one eye only at baseline and/or the final record |
| **Sensitivity Analysis – Final retinopathy grade in those with a baseline record (a subset of cohort B4) (cohort B6)** | Those with retinopathy data in both eyes (if on different dates then within a year of each other) | Those with retinopathy data in both eyes | Those with retinopathy data in neither eye or one eye only at baseline and/or the final record  Those with final retinopathy data on each eye more than 1 year apart |
| **Sensitivity Analysis – highest ever post-randomisation retinopathy grade (cohort B7)** | Those with retinopathy data in both eyes | Not relevant | Those with retinopathy data in neither eye or one eye only post-randomisation |

*All analyses will ignore pre-baseline and post-trial retinopathy and maculopathy data.

**The majority of individuals had retinopathy data in both eyes on the same final record. If retinopathy is missing for one eye on the final record, an earlier (post-randomisation) record will be used as the final grade in the missing eye

***The majority of individuals had retinopathy data in both eyes on the same baseline record. If retinopathy data is missing for one eye in the baseline record closest to randomisation, an earlier record may be used for the missing eye, so long as both baseline records occurred in the two years prior to randomisation.

# Appendix 3: Summary of Objectives, Outcomes, Data Sources and Analysis Populations

| **Objective** | **Outcomes** | **Data source** | **Analysis cohort** |
| --- | --- | --- | --- |
| i) To determine whether aspirin or omega-3 fatty acids alter the course of diabetic retinopathy or diabetic maculopathy | **1^o^ efficacy:** Referable diabetic eye disease for those with in-trial retinopathy data (R_2_,R_3a_ or M_1_) | DESP-linkage | B1 |
|  | **2^o^ efficacy:** Referable diabetic eye disease restricted to those with a baseline record (R_0_ or R_1_) and M_0_ 🡪(R_2_,R_3a_ or M_1_) | DESP-linkage | B2 |
|  | **2^o^ efficacy:** Any progression in retinopathy grade | DESP-linkage | B3 |
|  | **2^o^ efficacy:** all incident reports of DMO (M_1_ from the DESP-linkage dataset and F426 codes from either the VFQ form or ASCEND follow-up forms) | Pt-reported outcomes, VFQ and DESP-linkage | A, B2 and C |
|  | **3^o^ efficacy:** 1^o^ efficacy by baseline characteristic | DESP-linkage | B1 |
|  | **3^o^ efficacy:** Final retinopathy grade | DESP-linkage | B4 |
| ii) To compare differences in visual acuity scores between treatment arms | **3^o^ efficacy**: worsening of visual acuity by ≥3 points (≥15 ETDRS letters) | DESP-linkage | B5 |
| iii) To determine the role of aspirin and separately, omega-3 fatty acids on incident diagnoses of age-related macular degeneration | **2^o^ efficacy:** Incident diagnoses of AMD | Pt-reported outcomes and VFQ | A and C |
| iv) To compare differences between treatment arms in composite visual function scores derived from the National Eye Institute’s Visual Function Questionnaire-25 (NEI-VFQ-25) | **2^o^ efficacy:** Composite NEI-VFQ-25 score | VFQ | C |
|  | **3^o^ efficacy:** Sub-domain scores from the NEI-VFQ-25 | VFQ | C |
| v) To characterise the occurrence and severity of participant-reported eye bleeding events by treatment allocation | **1^o^ safety:** Confirmed sight-threatening eye bleeds | Pt-reported outcomes | A |
|  | **2^o^ safety:** Any eye bleed subdivided by severity | Pt-reported outcomes | A and C |
| vi) To identify the clinical and demographic characteristics that are associated with lower composite and subdomain scores on the NEI-VFQ-25, and to consider how concerns about eye health impact activities of daily living and emotional well-being  **(Analyses relevant to this objective are not defined in this DAP)** | **Exploratory:** Multivariate analyses of clinical and demographic characteristics associated with lower composite and subdomain scores on the NEI-VFQ-25. | VFQ | C |
| vii) To describe incident reports of other eye diseases between the treatment arms, such as cataracts, glaucoma, retinal vein thrombosis, infections and ocular nerve palsies | **Exploratory outcome:** descriptive data on incident reports of other eye diseases of interest | Pt-reported outcomes | A and C |

# Appendix 4: Visual Acuity

| **LogMar Chart** | **Snellen Chart (UK)** | **Snellen Chart (US)** | **ETDRS Letter Score** | **VAS Score (Visual acuity score)** | **ASCEND-Eye Recoded Score** |
| --- | --- | --- | --- | --- | --- |
| -0.2 | 6/3.8 | 20/12.5 | 95 | 110 | 1 |
| -0.18 | 6/4 | 20/13 |  |  | 2 |
| -0.1 | 6/4.8 | 20/16 | 90 | 105 | 3 |
| -0.08 | 6/5 | 20/17 |  |  | 4 |
| 0 | 6/6 | 20/20 | 85 | 100 | 5 |
| 0.1 | 6/7.5 | 20/25 | 80 | 95 | 6 |
| 0.2 | 6/9.5 | 20/32 | 75 | 90 | 7 |
| 0.3 | 6/12 | 20/40 | 70 | 85 | 8 |
| 0.4 | 6/15 | 20/50 | 65 | 80 | 9 |
| 0.48 | 6/18 | 20/60 |  |  | 10 |
| 0.5 | 6/19 |  | 60 | 75 | 11 |
| 0.6 | 6/24 | 20/80 | 55 | 70 | 12 |
| 0.7 | 6/30 | 20/100 | 50 | 65 | 13 |
| 0.78 | 6/36 | 20/120 |  |  | 14 |
| 0.8 | 6/38 | 20/125 | 45 | 60 | 15 |
| 0.9 | 6/48 | 20/160 | 40 | 55 | 16 |
| 1 | 6/60 | 20/200 | 35 | 50 | 17 |
| 1.1 | 6/76 | 20/250 | 30 | 45 | 18 |
| 1.2 | 6/95 | 20/320 | 25 | 40 | 19 |
| 1.3 | 3/60 | 20/400 | 20 | 35 | 20 |
| 1.4 | 3/75 | 20/500 | 15 | 30 | 21 |
| 1.48 | 2/60 |  |  |  | 22 |
| 1.5 | 2/63 | 20/630 | 10 | 25 | 23 |
| 1.6 | 2/80 | 20/800 | 5 | 20 | 24 |
| 1.7 | 2/100 | 20/1000 |  | 15 | 25 |
| 1.78 | 1/60 |  |  |  | 26 |
| 1.8 | 1/63 | 20/1250 |  | 10 | 27 |
| 1.9 | 1/79 | 20/1600 |  | 5 | 28 |
| 2.0 | 1/100 | 20/2000 | 2 | 0 | 29 |
| (CF) Counts fingers at 1m | CF |  |  |  | 30 |
| (HM) Hand movements | HM |  |  |  | 31 |
| (PL) Perceives light | PL |  |  |  | 32 |
| (NPL) No perception of light | NPL |  |  |  | 33 |
| Enucleated eye | Enucleated eye |  |  |  | 34 |

# Appendix 5: Other Eye Events

| **Read code** | **Description** |
| --- | --- |
| F40 | Eye infection |
| F401 | Other ophthalmitis |
| F41Z | Retinal detachment/surgery |
| F423 | Amaurosis fugax/transient visual loss |
| F430 | Eye inflammation unspecified |
| F440 | Iritis/iridocyclitis/uveitis |
| F45 | Glaucoma/surgery for/trabeculectomy |
| F48 | Visual disturbances |
| F490 | Blindness |
| F4B | Corneal disorder |
| F4C0 | Conjunctivitis |
| F4E | Eyelid disorder |
| G745 | Retinal artery occlusion/retinal thrombosis/visual loss thought ischaemic in nature |
| G755 | Giant cell arteritis/temporal arteritis |
| G825 | Retinal vein occlusion/blood clot in eye |
| F32Z, F310 | Cranial nerve palsy |

NB: the Read codes that are to be used for the relevant secondary and tertiary analyses include:

- F425 Macular/posterior pole degeneration
- F426 Macular oedema

# Appendix 6: Post-Adjudication Read Code Categories

| **Category** | **Description** | **Read Code** |
| --- | --- | --- |
| Sight-threatening eye bleed | - | F6xJx, F6xLx (excluding F6ALx codes), F6xSx (excluding F6ASx codes) |
| Non-serious eye bleed | - | F6xWN, F6xWU, F6ALx, F6ASx, F6xUN |
| Other medical retinal or uveal tract diagnosis | Other ophthalmitis | F401 |
|  | Retinal/posterior vitreous detachment | F41Z |
|  | Diabetic retinopathy/laser therapy for | F420 |
|  | Macular/posterior pole degeneration | F425 |
|  | Macular oedema | F426 |
|  | Iritis/iridocyclitis/uveitis | F440 |
|  | Glaucoma/surgery for/trabeculectomy | F45 |
|  | Retinitis pigmentosa | F7 |
| Eye procedure or investigation | Eye investigation | 1B8X |
|  | Eye injection | 724 |
|  | Eyelid/eyebrow operation | 721 |
|  | Other eye operation | 723 |
|  | Cataract operation | 7263 |
|  | Operation on the vitreous body/vitrectomy | 7270 |
| Orbital injury | Eye injury/trauma | SD8 |
| Cranial nerve palsy | - | F32Z, F310 |
| Cerebrovascular event | Amaurosis fugax | F423 |
|  | Retinal artery occlusion | G475 |
|  | Stroke codes | G60C, G640C,G640A |
| Other vascular | Retinal vein occlusion | G825 |
|  | Giant cell arteritis/temporal arteritis | G755 |
| Other non-serious eye condition | Conjunctivitis | F4C0 |
|  | Eyelid disorder | F4E |
|  | Eye infection | F40 |
|  | Visual loss/eyesight deterioration | 1B75 |
|  | Eye inflammation | F430 |
|  | Corneal disorder | F4B |
| Other |  |  |

## References

1. Aung T, Haynes R, Barton J*, et al.* Cost-effective recruitment methods for a large randomised trial in people with diabetes: A Study of Cardiovascular Events iN Diabetes (ASCEND). *Trials* 2016; 17(1): 286.

2. Bowman L, Mafham M, Stevens W*, et al.* ASCEND: A Study of Cardiovascular Events iN Diabetes: Characteristics of a randomized trial of aspirin and of omega-3 fatty acid supplementation in 15,480 people with diabetes. *American Heart Journal* 2018; 198: 135-44.

3. Bowman L, Mafham M, Wallendszus K*, et al.* Effects of Aspirin for Primary Prevention in Persons with Diabetes Mellitus. *N Engl J Med* 2018; 379(16): 1529-39.

4. Bowman L, Mafham M, Wallendszus K*, et al.* Effects of n-3 Fatty Acid Supplements in Diabetes Mellitus. *N Engl J Med* 2018; 379(16): 1540-50.

5. Gamble C, Krishan A, Stocken D*, et al.* Guidelines for the Content of Statistical Analysis Plans in Clinical Trials. *JAMA* 2017; 318(23): 2337-43.

6. Public Health England. NHS Diabetic Eye Screening Programme: grading definitions for referable disease. 2017. <https://assets.publishing.service.gov.uk/government/uploads/system/uploads/attachment_data/file/582710/Grading_definitions_for_referrable_disease_2017_new_110117.pdf> (accessed 05/04/2019).

7. Mangione CM, Lee PP, Gutierrez PR, *et al.* Development of the 25-list-item National Eye Institute Visual Function Questionnaire. *Archives of Ophthalmology* 2001; 119(7): 1050-8.

8. Klein R, Moss SE, Klein BE, Gutierrez P, Mangione CM. The NEI-VFQ-25 in people with long-term type 1 diabetes mellitus: the Wisconsin Epidemiologic Study of Diabetic Retinopathy. *Arch Ophthalmol* 2001; 119(5): 733-40.

9. European Medicines Agency. EU regulatory workshop – ophthalmology: visual function endpoints in clinical trials. (2012). <http://www.ema.europa.eu/docs/en_GB/document_library/Report/2012/09/WC500131815.pdf> (accessed 21/03/2018).

10. Peto R, Pike MC, Armitage P*, et al.* Design and analysis of randomized clinical trials requiring prolonged observation of each patient. I. Introduction and design. *British Journal of Cancer* 1976; 34(6): 585-612.

11. Peto R, Pike MC, Armitage P*, et al.* Design and analysis of randomized clinical trials requiring prolonged observation of each patient. II. analysis and examples. *British Journal of Cancer* 1977; 35(1): 1-39.

12. Early Treatment Diabetic Retinopathy Study Research Group. Effects of aspirin treatment on diabetic retinopathy. ETDRS Report Number 8. *Ophthalmology* 1991; 98: 757-65.

13. Comprehensive R Archive Network. <https://search.r-project.org/CRAN/refmans/powerSurvEpi/html/powerCT.default0.html> (accessed 27/04/2022).

14. Bunce C, Patel KV, Xing W, Freemantle N, Doré CJ, Group ObotOS. Ophthalmic statistics note 1: unit of analysis. *British Journal of Ophthalmology* 2014; 98(3): 408-12.

15. Armstrong RA. Statistical guidelines for the analysis of data obtained from one or both eyes. *Ophthalmic and Physiological Optics* 2013; 33(1): 7-14.

16. Yusuf S, Peto R, Lewis J, Collins R, Sleight P. Beta blockade during and after myocardial infarction: an overview of the randomized trials. *Progress in Cardiovascular Diseases* 1985; 27(5): 335-71.

17. Lee Johnson L, Shih JH. Chapter 23 - An introduction to survival analysis. In: Gallin JI, Ognibene FP, eds. Principles and Practice of Clinical Research (Third Edition). Boston: Academic Press; 2012: 285-93.

18. Valenta Z, Pitha J, Poledne R. Proportional odds logistic regression--effective means of dealing with limited uncertainty in dichotomizing clinical outcomes. *Stat Med* 2006; 25(24): 4227-34.

19. Roozenbeek B, Lingsma HF, Perel P*, et al.* The added value of ordinal analysis in clinical trials: an example in traumatic brain injury. *Critical Care* 2011; 15(3): R127.

20. Collins R, MacMahon S. Reliable assessment of the effects of treatment on mortality and major morbidity, I: clinical trials. *The Lancet* 2001; 357(9253): 373-80.

21. Gail M, Simon R. Testing for qualitative interactions between treatment effects and patient subsets. *Biometrics* 1985; 41(2): 361-72.

22. Inker LA, Schmid CH, Tighiouart H*, et al.* Estimating Glomerular Filtration Rate from Serum Creatinine and Cystatin C. *New England Journal of Medicine* 2012; 367(1): 20-9.

## ASCEND Follow-Up Questionnaire


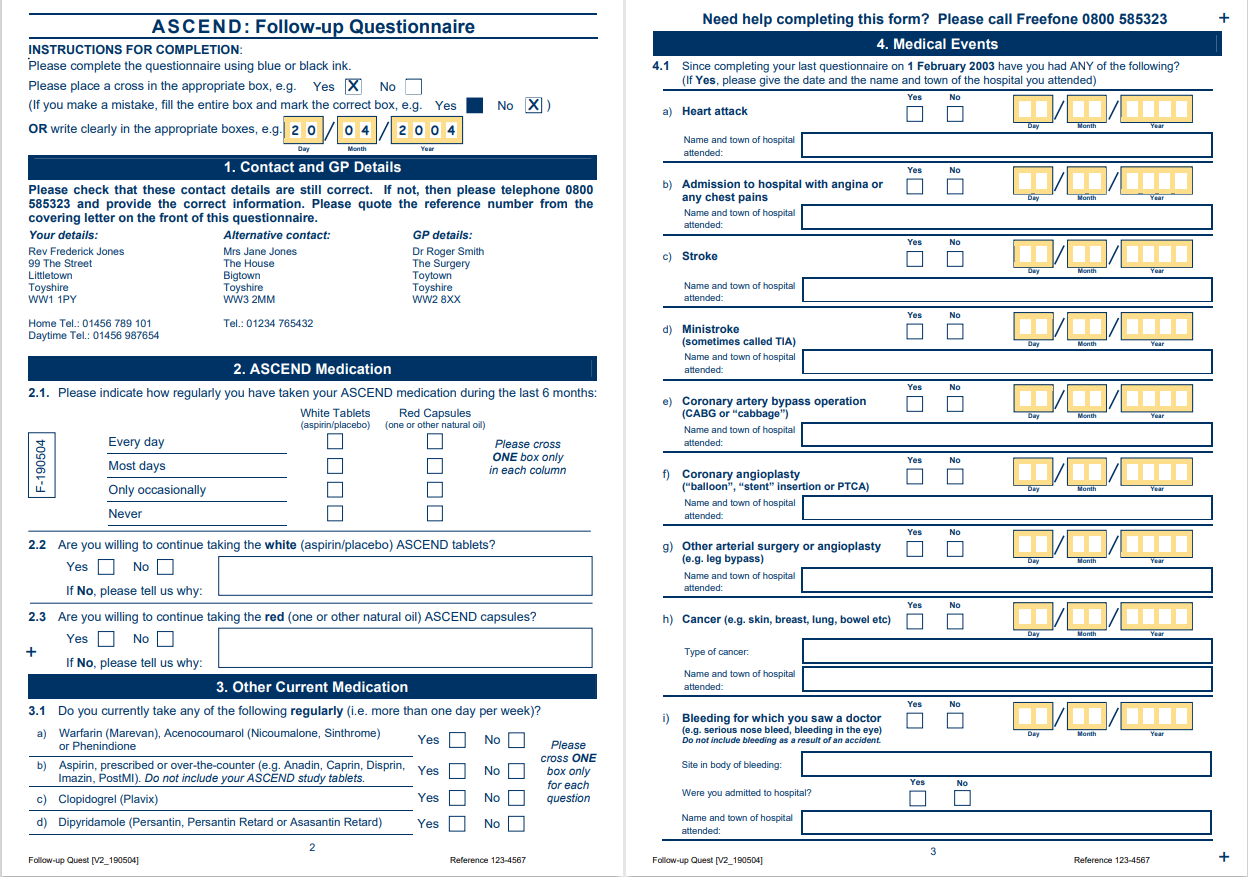


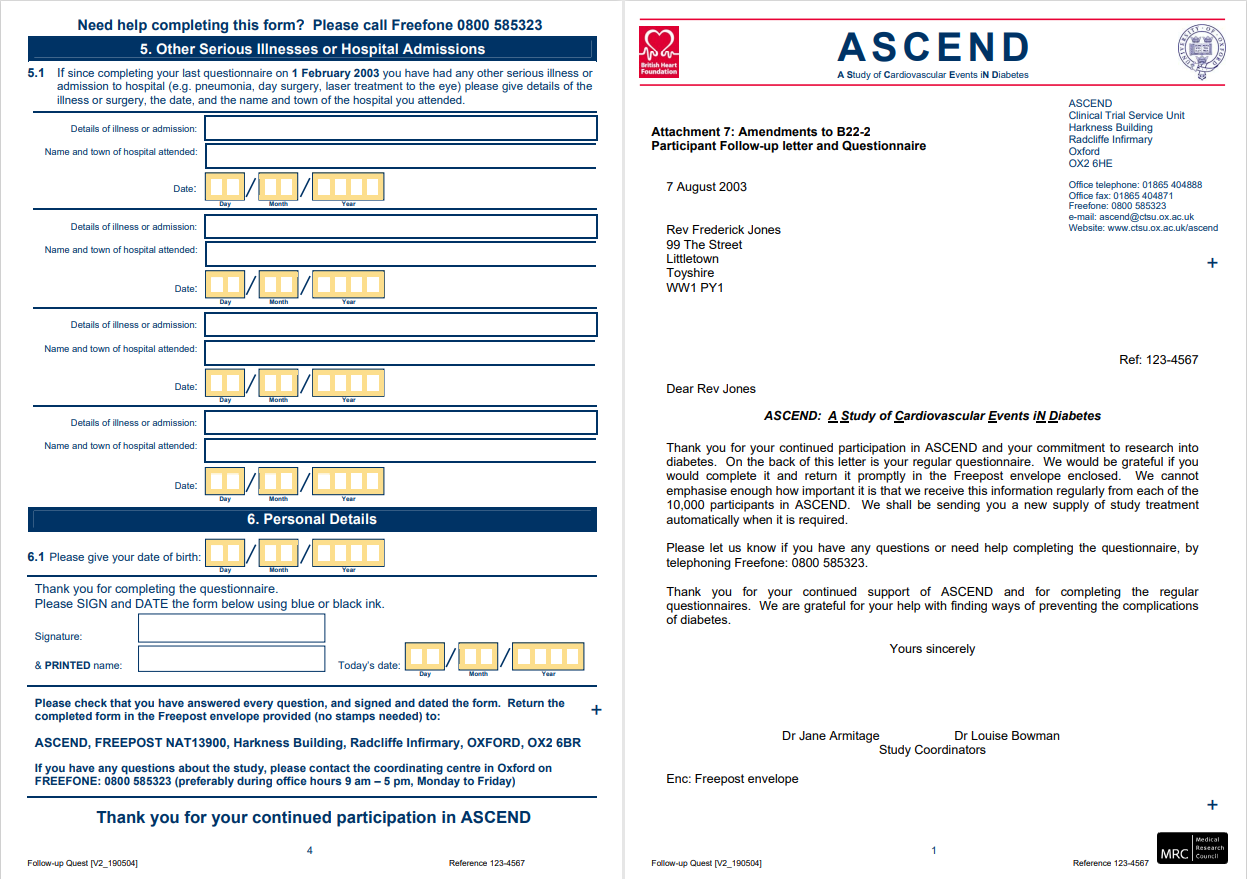


## ASCEND-Eye Visual Function Questionnaire


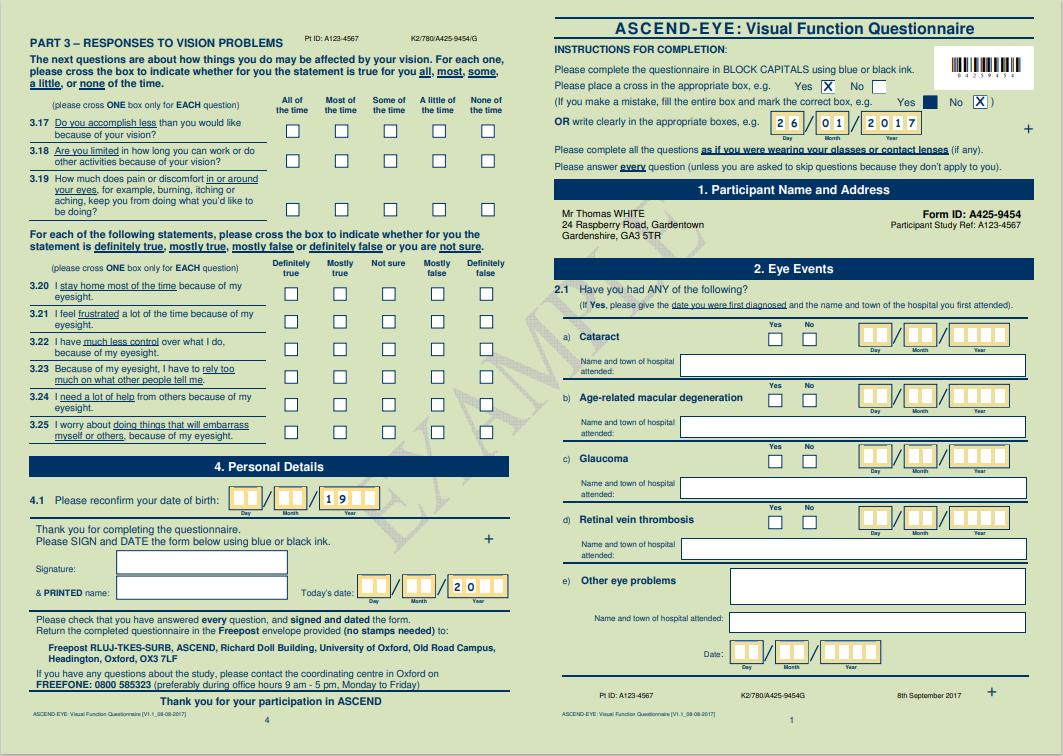


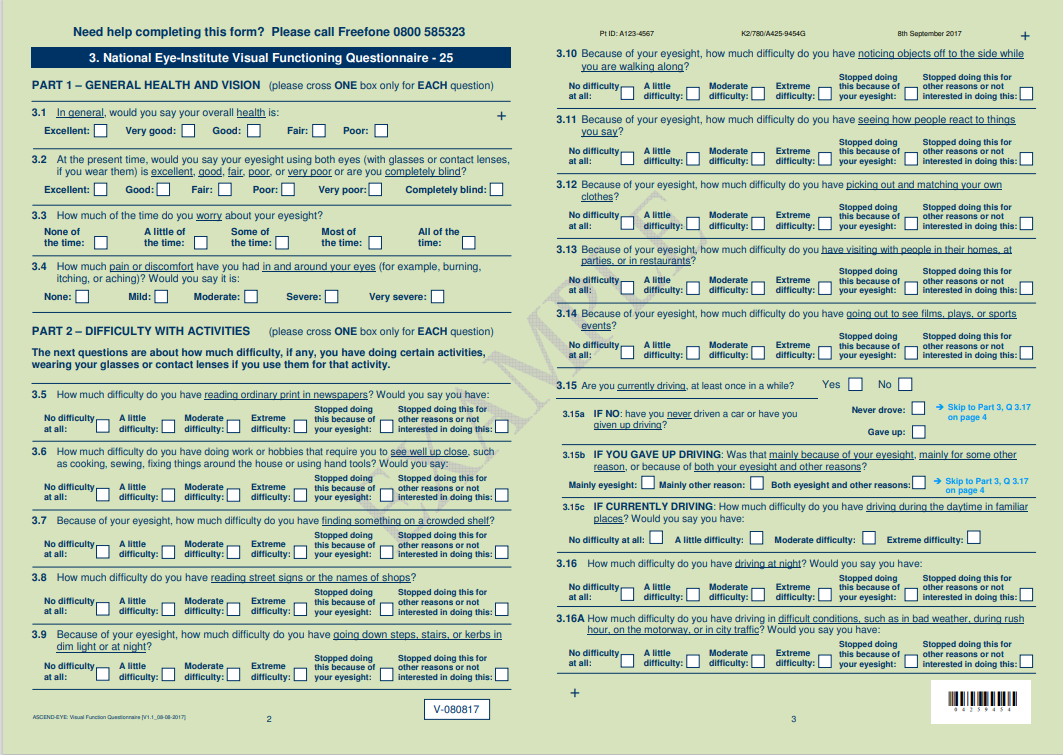


## Figure S1 Consort diagram for the full randomised ASCEND population – Aspirin arm

Excluded (10,982)

Unwilling to continue (5345)

Randomization questionnaire not returned (2832)

Ineligible (1453)

Medical advice (556)

Side effects of study medication (419)

Other reasons (377)

Assigned active aspirin and active omega-3 FAs (3870)

Assigned active aspirin and placebo omega-3 FAs (3870)

Assigned placebo aspirin and placebo omega-3 FAs (3870)

Assigned placebo aspirin and active omega-3 FAs (3870)

Assigned to active aspirin (7740)

Assigned to placebo aspirin (7740)

Incomplete follow-up information (69)

Withdrew consent (35)

Moved abroad (17)

Lost to follow-up (17)

Complete follow-up information (7671)

Incomplete follow-up information (70)

Withdrew consent (30)

Moved abroad (14)

Lost to follow-up (26)

Complete follow-up information (7670)

Analyzed (7740)

Analyzed (7740)

Excluded (94,792)

Declined to join the study (66,498)

Ineligible (14,451)

Incomplete questionnaire (2465)

Recruitment target reached (11,378)

Assessed for eligibility (121,254)

Entered run-in (26,462)

Randomized (15,480)

## Figure S2 Consort diagram for the full randomised ASCEND population – Omega-3 FAs arm

Excluded (10,982)

Unwilling to continue (5345)

Randomization questionnaire not returned (2832)

Ineligible (1453)

Medical advice (556)

Side effects of study medication (419)

Other reasons (377)

Excluded (94,792)

Declined to join the study (66,498)

Ineligible (14,451)

Incomplete questionnaire (2465)

Recruitment target reached (11,378)

Assigned placebo omega-3 FAs and placebo aspirin (3870)

Assigned placebo omega-3 FAs and active aspirin (3870)

Assigned active omega-3 FAs and placebo aspirin (3870)

Assigned active omega-3 FAs and active aspirin (3870)

Assigned to active omega-3 FAs (7740)

Assigned to placebo omega-3 FAs (7740)

Incomplete follow-up information (68)

Withdrew consent (26)

Moved abroad (19)

Lost to follow-up (23)

Complete follow-up information (7672)

Incomplete follow-up information (71)

Withdrew consent (39)

Moved abroad (12)

Lost to follow-up (20)

Complete follow-up information (7669)

Analyzed (7740)

Analyzed (7740)

Assessed for eligibility (121,254)

Entered run-in (26,462)

Randomized (15,480)

## Figure S3 Consort diagram from the DESP-linked population - Omega-3 FAs arm

Randomized into ASCEND (15,480)

Assigned active omega-3 FAs and active aspirin (3870)

Assigned active omega-3 FAs and placebo aspirin (3870)

Assigned placebo omega-3 FAs and active aspirin (3870)

Assigned placebo omega-3 FAs and placebo aspirin (3870)

Assigned to active omega-3 FAs (7740)

Assigned to placebo omega-3 FAs(7740)

Excluded:

Withdrew consent during ASCEND (60)

Resided in Scotland (323) or Northern Ireland (183)

No NHS number (6)

Attended a GP practice in England that did not register their data with PHE (1252)

Excluded:

No data during the scheduled treatment period (170)

No retinopathy or maculopathy data during the scheduled treatment period (5)

Excluded:

Participants who were not linked (573)

Linked dataset (7535)

Box B. Total no. of collaborating DESPs: 28

Data controllers:

1. Health Intelligence Ltd (11; DESPs; 3817)
2. Northgate Ltd (7 DESPs; 1700)
3. Diabetic Eye Screening Wales (1 DESP; 853)
4. NHS Trusts via Northgate Ltd (8 DESPs; 1914)
5. NHS Trust directly (1 DESP; 41)

Box A. Total no. of DESPs invited to collaborate: 59

Data controllers:

1. Health Intelligence Ltd (11 DESPs; 3817)

2. Northgate Ltd (7 DESPs; 1700)

3. Diabetic Eye Screening Wales (1 DESP; 853)

4. NHS Trusts (40 DESPs; 7503)

Identifiers sent to data controllers for linkage (8108)*

Eligible to be included in DESP-linkage (13,656)*

Excluded:

No response to collaboration invite (18 DESPs; 2411)

Declined collaboration (1 DESP; 621)

Served by a DESP that was willing to collaborate but which suspended non-Covid-19 research at the start of the pandemic (12 DESPs; 2516)

*The no. of participants linked to each data controller in boxes A and B exceeds the number shown in the vertical flow boxes because some participants were linked to more than one DESP during the scheduled treatment period of ASCEND

Assigned active omega-3 FAs and active aspirin (1843)

Assigned active omega-3 FAs and placebo aspirin (1849)

Assigned placebo omega-3 FAs and active aspirin (1844)

Assigned placebo omega-3 FAs and placebo aspirin (1824)

Assigned to active omega-3 FAs and analyzed (3692)

Assigned to placebo omega-3 FAs and analyzed (3668)

## Figure S4 Consort Diagram for VFQ Cohort - Omega-3 FAs Arm

Randomiszd into ASCEND (15,480)

Assigned active omega-3 FAs and active aspirin (3870)

Assigned active omega-3 FAs and placebo aspirin (3870)

Assigned placebo omega-3 FAs and active aspirin (3870)

Assigned placebo omega-3 FAs and placebo aspirin (3870)

Assigned to active omega-3 FAs (7740)

Assigned to placebo omega-3 FAs (7740)

Excluded:

Withdrew consent during ASCEND (60)

Died during the ASCEND trial (1704)

On GP or registry follow-up at the end of ASCEND (2415)

Eligible to be included in VFQ exercise (11,301)

Excluded:

Non-responders (2455)

Assigned active omega-3 FAs and active aspirin (2213)

Assigned active omega-3 FAs and placebo aspirin (2204)

Assigned placebo omega-3 FAs and active aspirin (2234)

Assigned placebo omega-3 FAs and placebo aspirin (2195)

Assigned to active omega-3 FAs and analyzed (4417)

Assigned to placebo omega-3 FAs and analyzed (4429)

## Table S1: Baseline Characteristics of the Full ASCEND Population (Cohort A)

| **Baseline Characteristic** | **Aspirin** | | | | **Omega-3 FAs** | | | | **Overall**  **(n=15,480)** | |
| --- | --- | --- | --- | --- | --- | --- | --- | --- | --- | --- |
|  | **Active**  **(n=7740)** | | **Placebo**  **(n=7740)** | | **Active**  **(n=7740)** | | **Placebo**  **(n=7740)** | |  |  |
|  |  |  |  |  |  |  |  |  |  |  |
| **Age at randomization**  **(years)** |  |  |  |  |  |  |  |  |  |  |
| Mean (SD) | 63.2±9.2 | | 63.3±9.2 | | 63.3±9.2 | | 63.3±9.2 | | 63.3±9.2 | |
| <60 | 2795 | (36.1%) | 2795 | (36.1%) | 2791 | (36.1%) | 2799 | (36.2%) | 5590 | (36.1%) |
| ≥60<70 | 3123 | (40.3%) | 3124 | (40.4%) | 3127 | (40.4%) | 3120 | (40.3%) | 6247 | (40.4%) |
| ≥70 | 1822 | (23.5%) | 1821 | (23.5%) | 1822 | (23.5%) | 1821 | (23.5%) | 3643 | (23.5%) |
|  |  |  |  |  |  |  |  |  |  |  |
| **Sex** |  |  |  |  |  |  |  |  |  |  |
| Male | 4843 | (62.6%) | 4841 | (62.5%) | 4842 | (62.6%) | 4842 | (62.6%) | 9684 | (62.6%) |
| Female | 2897 | (37.4%) | 2899 | (37.5%) | 2898 | (37.4%) | 2898 | (37.4%) | 5796 | (37.4%) |
|  |  |  |  |  |  |  |  |  |  |  |
| **Type of diabetes*** |  |  |  |  |  |  |  |  |  |  |
| Type 1 | 458 | (5.9%) | 453 | (5.9%) | 460 | (5.9%) | 451 | (5.8%) | 911 | (5.9%) |
| Type 2 | 7282 | (94.1%) | 7287 | (94.1%) | 7280 | (94.1%) | 7289 | (94.2%) | 14569 | (94.1%) |
|  |  |  |  |  |  |  |  |  |  |  |
| **Duration of diabetes**  **(years)** |  |  |  |  |  |  |  |  |  |  |
| Median (IQR) | 7(3-13) | | 7 (3-13) | | 7 (3-12) | | 7(3-13) | | 7(3-13) | |
| ≥0<5 years | 2446 | (31.6%) | 2445 | (31.6%) | 2443 | (31.6%) | 2448 | (31.6%) | 4891 | (31.6%) |
| ≥5<10 years | 2166 | (28.0%) | 2168 | (28.0%) | 2167 | (28.0%) | 2167 | (28.0%) | 4334 | (28.0%) |
| ≥10<20 years | 1767 | (22.8%) | 1770 | (22.9%) | 1770 | (22.9%) | 1767 | (22.8%) | 3537 | (22.8%) |
| ≥20 years | 934 | (12.1%) | 928 | (12.0%) | 932 | (12.0%) | 930 | (12.0%) | 1862 | (12.0%) |
| Unknown | 427 | (5.5%) | 429 | (5.5%) | 428 | (5.5%) | 428 | (5.5%) | 856 | (5.5%) |
|  |  |  |  |  |  |  |  |  |  |  |
| **Diabetes management** |  |  |  |  |  |  |  |  |  |  |
| Diet only | 1271 | (16.4%) | 1258 | (16.3%) | 1229 | (15.9%) | 1300 | (16.8%) | 2529 | (16.3%) |
| Oral hypoglycaemic agent(s) only | 4510 | (58.3%) | 4510 | (58.3%) | 4533 | (58.6%) | 4487 | (58.0%) | 9020 | (58.3%) |
| Insulin +/-oral hypoglycaemic agent(s) | 1959 | (25.3%) | 1972 | (25.5%) | 1978 | (25.6%) | 1953 | (25.2%) | 3931 | (25.4%) |
|  |  |  |  |  |  |  |  |  |  |  |
| **Participant-reported diabetic retinopathy** |  |  |  |  |  |  |  |  |  |  |
| Yes | 1526 | (19.7%) | 1497 | (19.3%) | 1549 | (20.0%) | 1474 | (19.0%) | 3023 | (19.5%) |
| No | 6144 | (79.4%) | 6169 | (79.7%) | 6121 | (79.1%) | 6192 | (80.0%) | 12313 | (79.5%) |
| Unknown | 70 | (0.9%) | 74 | (1.0%) | 70 | (0.9%) | 74 | (1.0%) | 144 | (0.9%) |
|  |  |  |  |  |  |  |  |  |  |  |
| **Participant-reported treatment for hypertension** |  |  |  |  |  |  |  |  |  |  |
| Yes | 4766 | (61.6%) | 4767 | (61.6%) | 4768 | (61.6%) | 4765 | (61.6%) | 9533 | (61.6%) |
| No | 2919 | (37.7%) | 2916 | (37.7%) | 2917 | (37.7%) | 2918 | (37.7%) | 5835 | (37.7%) |
| Unknown | 55 | (0.7%) | 57 | (0.7%) | 55 | (0.7%) | 57 | (0.7%) | 112 | (0.7%) |
|  |  |  |  |  |  |  |  |  |  |  |
| **Systolic blood pressure**  **(mmHg)** † |  |  |  |  |  |  |  |  |  |  |
| Mean (SD) | 136.1±15.2 | | 136.2±15.3 | | 136.2±15.4 | | 136.2±15.1 | | 136.2±15.3 | |
| <130 | 1694 | (21.9%) | 1700 | (22.0%) | 1695 | (21.9%) | 1699 | (22.0%) | 3394 | (21.9%) |
| ≥130<140 | 1550 | (20.0%) | 1541 | (19.9%) | 1547 | (20.0%) | 1544 | (19.9%) | 3091 | (20.0%) |
| ≥140 | 2263 | (29.2%) | 2292 | (29.6%) | 2279 | (29.4%) | 2276 | (29.4%) | 4555 | (29.4%) |
| Unknown | 2233 | (28.9%) | 2207 | (28.5%) | 2219 | (28.7%) | 2221 | (28.7%) | 4440 | (28.7%) |
|  |  |  |  |  |  |  |  |  |  |  |
| **Diastolic blood pressure**  **(mmHg)** † |  |  |  |  |  |  |  |  |  |  |
| Mean (SD) | 77.0±9.4 | | 77.2±9.5 | | 77.1±9.5 | | 77.1±9.5 | | 77.1±9.5 | |
| <75 | 2113 | (27.3%) | 2110 | (27.3%) | 2097 | (27.1%) | 2126 | (27.5%) | 4223 | (27.3%) |
| ≥75 <85 | 2325 | (30.0%) | 2294 | (29.6%) | 2331 | (30.1%) | 2288 | (29.6%) | 4619 | (29.8%) |
| ≥85 | 1066 | (13.8%) | 1125 | (14.5%) | 1089 | (14.1%) | 1102 | (14.2%) | 2191 | (14.2%) |
| Unknown | 2236 | (28.9%) | 2211 | (28.6%) | 2223 | (28.7%) | 2224 | (28.7%) | 4447 | (28.7%) |
|  |  |  |  |  |  |  |  |  |  |  |
| **Body mass index**  **(kg/m^2^)** ‡ |  |  |  |  |  |  |  |  |  |  |
| Mean (SD) | 30.8±6.3 | | 30.6±6.3 | | 30.7±6.3 | | 30.8±6.2 | | 30.7±6.3 | |
| <25 | 1080 | (14.0%) | 1169 | (15.1%) | 1129 | (14.6%) | 1120 | (14.5%) | 2249 | (14.5%) |
| ≥25, <30 | 2753 | (35.6%) | 2776 | (35.9%) | 2771 | (35.8%) | 2758 | (35.6%) | 5529 | (35.7%) |
| ≥30<35 | 2150 | (27.8%) | 2090 | (27.0%) | 2131 | (27.5%) | 2109 | (27.2%) | 4240 | (27.4%) |
| ≥35 | 1515 | (19.6%) | 1446 | (18.7%) | 1453 | (18.8%) | 1508 | (19.5%) | 2961 | (19.1%) |
| Unknown | 242 | (3.1%) | 259 | (3.3%) | 256 | (3.3%) | 245 | (3.2%) | 501 | (3.2%) |
|  |  |  |  |  |  |  |  |  |  |  |
|  |  |  |  |  |  |  |  |  |  |  |
| **Cigarette smoking** |  |  |  |  |  |  |  |  |  |  |
| Current | 639 | (8.3%) | 640 | (8.3%) | 639 | (8.3%) | 640 | (8.3%) | 1279 | (8.3%) |
| Former | 3526 | (45.6%) | 3525 | (45.5%) | 3527 | (45.6%) | 3524 | (45.5%) | 7051 | (45.5%) |
| Never | 3489 | (45.1%) | 3488 | (45.1%) | 3489 | (45.1%) | 3488 | (45.1%) | 6977 | (45.1%) |
| Unknown | 86 | (1.1%) | 87 | (1.1%) | 85 | (1.1%) | 88 | (1.1%) | 173 | (1.1%) |
|  |  |  |  |  |  |  |  |  |  |  |
| **Non-study medication** |  |  |  |  |  |  |  |  |  |  |
| ACE-inhibitor or ARB | 4520 | (58.4%) | 4535 | (58.6%) | 4569 | (59.0%) | 4486 | (58.0%) | 9055 | (58.5%) |
| Aspirin use before screening | 2740 | (35.4%) | 2768 | (35.8%) | 2744 | (35.5%) | 2764 | (35.7%) | 5508 | (35.6%) |
| Thiazide or related diuretic | 1480 | (19.1%) | 1477 | (19.1%) | 1448 | (18.7%) | 1509 | (19.5%) | 2957 | (19.1%) |
| Calcium channel blocker | 1926 | (24.9%) | 1847 | (23.9%) | 1912 | (24.7%) | 1861 | (24.0%) | 3773 | (24.4%) |
| Statin | 5854 | (75.6%) | 5799 | (74.9%) | 5791 | (74.8%) | 5862 | (75.7%) | 11653 | (75.3%) |
|  |  |  |  |  |  |  |  |  |  |  |
| **Total cholesterol (mmol/L)** |  |  |  |  |  |  |  |  |  |  |
| Mean (SD) | 4.2±0.9 | | 4.2±0.9 | | 4.2±0.9 | | 4.2±0.9 | | 4.2±0.9 | |
| <4 | 2293 | (29.6%) | 2261 | (29.2%) | 2284 | (29.5%) | 2270 | (29.3%) | 4554 | (29.4%) |
| ≥4 <5 | 1875 | (24.2%) | 1920 | (24.8%) | 1900 | (24.5%) | 1895 | (24.5%) | 3795 | (24.5%) |
| ≥5 | 735 | (9.5%) | 735 | (9.5%) | 745 | (9.6%) | 725 | (9.4%) | 1470 | (9.5%) |
| Not available | 2837 | (36.7%) | 2824 | (36.5%) | 2811 | (36.3%) | 2850 | (36.8%) | 5661 | (36.6%) |
|  |  |  |  |  |  |  |  |  |  |  |
| **HDL cholesterol (mmol/L)** |  |  |  |  |  |  |  |  |  |  |
| Mean (SD) | 1.3±0.4 | | 1.3±0.4 | | 1.3±0.4 | | 1.3±0.4 | | 1.3±0.4 | |
| <1 | 1116 | (14.4%) | 1054 | (13.6%) | 1104 | (14.3%) | 1066 | (13.8%) | 2170 | (14.0%) |
| ≥1<1.5 | 2708 | (35.0%) | 2815 | (36.4%) | 2775 | (35.9%) | 2748 | (35.5%) | 5523 | (35.7%) |
| ≥1.5 | 1072 | (13.9%) | 1035 | (13.4%) | 1036 | (13.4%) | 1071 | (13.8%) | 2107 | (13.6%) |
| Not available | 2844 | (36.7%) | 2836 | (36.6%) | 2825 | (36.5%) | 2855 | (36.9%) | 5680 | (36.7%) |
|  |  |  |  |  |  |  |  |  |  |  |
| **Non-HDL cholesterol (mmol/L)** |  |  |  |  |  |  |  |  |  |  |
| Mean (SD) | 2.9±0.9 | | 2.9±0.8 | | 2.9±0.9 | | 2.9±0.8 | | 2.9±0.8 | |
| <2.5 | 1691 | (21.8%) | 1699 | (22.0%) | 1689 | (21.8%) | 1701 | (22.0%) | 3390 | (21.9%) |
| ≥2.5 <3.5 | 2213 | (28.6%) | 2180 | (28.2%) | 2200 | (28.4%) | 2193 | (28.3%) | 4393 | (28.4%) |
| ≥3.5 | 992 | (12.8%) | 1025 | (13.2%) | 1026 | (13.3%) | 991 | (12.8%) | 2017 | (13.0%) |
| Not available | 2844 | (36.7%) | 2836 | (36.6%) | 2825 | (36.5%) | 2855 | (36.9%) | 5680 | (36.7%) |
|  |  |  |  |  |  |  |  |  |  |  |
| **Glycosylated haemoglobin - HbA1c** |  |  |  |  |  |  |  |  |  |  |
| IFCC (mmol/mol) mean (SD) | 54.7±12.9 | | 54.9±12.9 | | 54.9±13.0 | | 54.7±12.8 | | 54.8±12.9 | |
| DCCT (%) mean (SD) | 7.2±1.2 | | 7.2±1.2 | | 7.2±1.2 | | 7.2±1.2 | | 7.2±1.2 | |
| <48 (6.5) | 1661 | (21.5%) | 1611 | (20.8%) | 1631 | (21.1%) | 1641 | (21.2%) | 3272 | (21.1%) |
| ≥48 (6.5), <64 (8.0) | 2314 | (29.9%) | 2350 | (30.4%) | 2333 | (30.1%) | 2331 | (30.1%) | 4664 | (30.1%) |
| ≥64 (8.0) | 926 | (12.0%) | 951 | (12.3%) | 960 | (12.4%) | 917 | (11.8%) | 1877 | (12.1%) |
| Not available | 2839 | (36.7%) | 2828 | (36.5%) | 2816 | (36.4%) | 2851 | (36.8%) | 5667 | (36.6%) |
|  |  |  |  |  |  |  |  |  |  |  |
| **CKD-EPI estimated GFR**  **(ml/min/1.73m^2^)** § |  | |  |  |  |  |  |  |  |  |
| Mean (SD) | 85.2±21.1 | | 85.2±21.1 | | 85.2±21.3 | | 85.2±20.8 | | 85.2±21.1 | |
| ≥90 | 2265 | (29.3%) | 2258 | (29.2%) | 2256 | (29.1%) | 2267 | (29.3%) | 4523 | (29.2%) |
| ≥60<90 | 1986 | (25.7%) | 2030 | (26.2%) | 2032 | (26.3%) | 1984 | (25.6%) | 4016 | (25.9%) |
| <60 | 649 | (8.4%) | 627 | (8.1%) | 640 | (8.3%) | 636 | (8.2%) | 1276 | (8.2%) |
| Not available | 2840 | (36.7%) | 2825 | (36.5%) | 2812 | (36.3%) | 2853 | (36.9%) | 5665 | (36.6%) |
|  |  |  |  |  |  |  |  |  |  |  |
| **Urinary albumin:creatinine ratio (mg/mmol)¶** |  |  |  |  |  |  |  |  |  |  |
| Median (IQR) | 0.56  (0.00-1.33) | | 0.55  (0.18-1.34) | | 0.56  (0.18-1.36) | | 0.55  (0.14-1.32) | | 0.55  (0.16-1.34) | |
| <3 | 4267 | (55.1%) | 4259 | (55.0%) | 4253 | (54.9%) | 4273 | (55.2%) | 8526 | (55.1%) |
| ≥3 | 621 | (8.0%) | 627 | (8.1%) | 657 | (8.5%) | 591 | (7.6%) | 1248 | (8.1%) |
| Not available | 2852 | (36.8%) | 2854 | (36.9%) | 2830 | (36.6%) | 2876 | (37.2%) | 5706 | (36.9%) |
|  |  |  |  |  |  |  |  |  |  |  |
| **Townsend Deprivation Index**** |  |  |  |  |  |  |  |  |  |  |
| <-3 | 2502 | (32.3%) | 2602 | (33.6%) | 2573 | (33.2%) | 2531 | (32.7%) | 5104 | (33.0%) |
| ≥-3<0 | 3023 | (39.1%) | 2999 | (38.7%) | 2954 | (38.2%) | 3068 | (39.6%) | 6022 | (38.9%) |
| ≥0<2 | 1037 | (13.4%) | 1000 | (12.9%) | 1055 | (13.6%) | 982 | (12.7%) | 2037 | (13.2%) |
| ≥2<4 | 686 | (8.9%) | 629 | (8.1%) | 654 | (8.4%) | 661 | (8.5%) | 1315 | (8.5%) |
| ≥4<6 | 351 | (4.5%) | 352 | (4.5%) | 348 | (4.5%) | 355 | (4.6%) | 703 | (4.5%) |
| ≥6 | 121 | (1.6%) | 140 | (1.8%) | 137 | (1.8%) | 124 | (1.6%) | 261 | (1.7%) |
| Unknown | 20 | (0.3%) | 18 | (0.2%) | 19 | (0.2%) | 19 | (0.2%) | 38 | (0.2%) |
|  |  |  |  |  |  |  |  |  |  |  |
| **Ethnic origin** |  |  |  |  |  |  |  |  |  |  |
| White | 7467 | (96.5%) | 7468 | (96.5%) | 7467 | (96.5%) | 7468 | (96.5%) | 14935 | (96.5%) |
| Indian/Pakistani/Bangladeshi | 91 | (1.2%) | 93 | (1.2%) | 92 | (1.2%) | 92 | (1.2%) | 184 | (1.2%) |
| African/Caribbean | 70 | (0.9%) | 70 | (0.9%) | 70 | (0.9%) | 70 | (0.9%) | 140 | (0.9%) |
| Other/unknown | 112 | (1.4%) | 109 | (1.4%) | 111 | (1.4%) | 110 | (1.4%) | 221 | (1.4%) |
|  |  |  |  |  |  |  |  |  |  |  |

DCCT = Diabetes Control and Complications Trial; FAs = Fatty acids; GFR = Glomerular Filtration Rate; HDL=High-density lipoprotein; IFCC =International Federation of Clinical Chemistry; SD = Standard Deviation; IQR = Interquartile range

Figures presented are counts with percentages unless otherwise stated. Percentages may not total 100 because of rounding.

*The presence of type 2 diabetes was based on a broad clinical definition involving the participant’s age at the diagnosis of diabetes, the use of insulin within one year after diagnosis, and the body-mass index.

† From blood and urine consent forms, generally before randomization.

‡ The body-mass index (the weight in kilograms divided by the square of the height in metres) was based on values for height and weight the participants reported on their randomization questionnaires.

§ Calculated from blood cystatin c concentration using the CKD-EPI formula.^381^

¶There was an analysis rule in ASCEND which stated that those with a below detectable threshold albumin component of their urinary albumin creatinine ratio would be recorded as zero. This applied to just over 25% of participants in the active group of the aspirin arm, and to just under 25% of participants in the placebo group. Hence the interquartile range included zero.

**Townsend scores of 0 represent an area with a UK-average level of deprivation, positive values indicate areas of above-average deprivation, and negative values indicate areas of below-average deprivation.

## Table S2: Baseline Characteristics of the DESP-Linked Population (Cohort B1)

| **Baseline Characteristic** | **Aspirin** | | | | **Omega-3 FAs** | | | | **Overall**  **(n=7360)** | |
| --- | --- | --- | --- | --- | --- | --- | --- | --- | --- | --- |
|  | **Active**  **(n=3687)** | | **Placebo**  **(n=3673)** | | **Active**  **(n=3692)** | | **Placebo**  **(n=3668)** | |  |  |
|  |  |  |  |  |  |  |  |  |  |  |
| **Age at randomization**  **(years)** |  |  |  |  |  |  |  |  |  |  |
| Mean (SD) | 63.5±8.9 | | 63.5±9.0 | | 63.5±8.9 | | 63.5±9.0 | | 63.5±8.9 | |
| <60 | 1287 | (34.9%) | 1287 | (35.0%) | 1283 | (34.8%) | 1291 | (35.2%) | 2574 | (35.0%) |
| ≥60<70 | 1530 | (41.5%) | 1517 | (41.3%) | 1535 | (41.6%) | 1512 | (41.2%) | 3047 | (41.4%) |
| ≥70 | 870 | (23.6%) | 869 | (23.7%) | 874 | (23.7%) | 865 | (23.6%) | 1739 | (23.6%) |
|  |  |  |  |  |  |  |  |  |  |  |
| **Sex** |  |  |  |  |  |  |  |  |  |  |
| Male | 2314 | (62.8%) | 2280 | (62.1%) | 2301 | (62.3%) | 2293 | (62.5%) | 4594 | (62.4%) |
| Female | 1373 | (37.2%) | 1393 | (37.9%) | 1391 | (37.7%) | 1375 | (37.5%) | 2766 | (37.6%) |
|  |  |  |  |  |  |  |  |  |  |  |
| **Type of diabetes*** |  |  |  |  |  |  |  |  |  |  |
| Type 1 | 186 | (5.0%) | 187 | (5.1%) | 181 | (4.9%) | 192 | (5.2%) | 373 | (5.1%) |
| Type 2 | 3501 | (95.0%) | 3486 | (94.9%) | 3511 | (95.1%) | 3476 | (94.8%) | 6987 | (94.9%) |
|  |  |  |  |  |  |  |  |  |  |  |
| **Duration of diabetes**  **(years)** |  |  |  |  |  |  |  |  |  |  |
| Median (IQR) | 7 (3-12) | | 6 (3-12) | | 7 (3-12) | | 6 (3-12) | | 6 (3-12) | |
| ≥0<5 years | 1185 | (32.1%) | 1202 | (32.7%) | 1198 | (32.4%) | 1189 | (32.4%) | 2387 | (32.4%) |
| ≥5<10 years | 1062 | (28.8%) | 1040 | (28.3%) | 1048 | (28.4%) | 1054 | (28.7%) | 2102 | (28.6%) |
| ≥10<20 years | 832 | (22.6%) | 849 | (23.1%) | 856 | (23.2%) | 825 | (22.5%) | 1681 | (22.8%) |
| ≥20 years | 397 | (10.8%) | 381 | (10.4%) | 381 | (10.3%) | 397 | (10.8%) | 778 | (10.6%) |
| Unknown | 211 | (5.7%) | 201 | (5.5%) | 209 | (5.7%) | 203 | (5.5%) | 412 | (5.6%) |
|  |  |  |  |  |  |  |  |  |  |  |
| **Diabetes management** |  |  |  |  |  |  |  |  |  |  |
| Diet only | 591 | (16.0%) | 621 | (16.9%) | 585 | (15.8%) | 627 | (17.1%) | 1212 | (16.5%) |
| Oral hypoglycaemic agents) only | 2253 | (61.1%) | 2219 | (60.4%) | 2265 | (61.3%) | 2207 | (60.2%) | 4472 | (60.8%) |
| Insulin +/- oral hypoglycaemic agent(s) | 843 | (22.9%) | 833 | (22.7%) | 842 | (22.8%) | 834 | (22.7%) | 1676 | (22.8%) |
|  |  |  |  |  |  |  |  |  |  |  |
| **Participant-reported diabetic retinopathy** |  |  |  |  |  |  |  |  |  |  |
| Yes | 662 | (18.0%) | 646 | (17.6%) | 674 | (18.3%) | 634 | (17.3%) | 1308 | (17.8%) |
| No | 2984 | (80.9%) | 3007 | (81.9%) | 2988 | (80.9%) | 3003 | (81.9%) | 5991 | (81.4%) |
| Unknown | 41 | (1.1%) | 20 | (0.5%) | 30 | (0.8%) | 31 | (0.8%) | 61 | (0.8%) |
|  |  |  |  |  |  |  |  |  |  |  |
| **Participant-reported treatment for hypertension** |  |  |  |  |  |  |  |  |  |  |
| Yes | 2252 | (61.1%) | 2250 | (61.3%) | 2265 | (61.3%) | 2237 | (61.0%) | 4502 | (61.2%) |
| No | 1408 | (38.2%) | 1390 | (37.8%) | 1395 | (37.8%) | 1403 | (38.2%) | 2798 | (38.0%) |
| Unknown | 27 | (0.7%) | 33 | (0.9%) | 32 | (0.9%) | 28 | (0.8%) | 60 | (0.8%) |
|  |  |  |  |  |  |  |  |  |  |  |
| **Systolic blood pressure**  **(mmHg)** † |  |  |  |  |  |  |  |  |  |  |
| Mean (SD) | 136.1±15.2 | | 136.0±15.3 | | 135.9±15.2 | | 136.1±15.2 | | 136.1±15.2 | |
| <130 | 823 | (22.3%) | 835 | (22.7%) | 835 | (22.6%) | 823 | (22.4%) | 1658 | (22.5%) |
| ≥130<140 | 744 | (20.2%) | 760 | (20.7%) | 744 | (20.2%) | 760 | (20.7%) | 1504 | (20.4%) |
| ≥140 | 1109 | (30.1%) | 1115 | (30.4%) | 1127 | (30.5%) | 1097 | (29.9%) | 2224 | (30.2%) |
| Unknown | 1011 | (27.4%) | 963 | (26.2%) | 986 | (26.7%) | 988 | (26.9%) | 1974 | (26.8%) |
|  |  |  |  |  |  |  |  |  |  |  |
| **Diastolic blood pressure**  **(mmHg)** † |  |  |  |  |  |  |  |  |  |  |
| Mean (SD) | 77.0±9.4 | | 77.2±9.4 | | 77.1±9.3 | | 77.1±9.5 | | 77.1±9.4 | |
| <75 | 1001 | (27.1%) | 1035 | (28.2%) | 1022 | (27.7%) | 1014 | (27.6%) | 2036 | (27.7%) |
| ≥75 <85 | 1156 | (31.4%) | 1141 | (31.1%) | 1162 | (31.5%) | 1135 | (30.9%) | 2297 | (31.2%) |
| ≥85 | 518 | (14.0%) | 531 | (14.5%) | 519 | (14.1%) | 530 | (14.4%) | 1049 | (14.3%) |
| Unknown | 1012 | (27.4%) | 966 | (26.3%) | 989 | (26.8%) | 989 | (27.0%) | 1978 | (26.9%) |
|  |  |  |  |  |  |  |  |  |  |  |
| **Body mass index**  **(kg/m^2^)** ‡ |  |  |  |  |  |  |  |  |  |  |
| Mean (SD) | 30.7±6.2 | | 30.6±6.2 | | 30.6±6.1 | | 30.7±6.2 | | 30.7±6.2 | |
| <25 | 520 | (14.1%) | 554 | (15.1%) | 535 | (14.5%) | 539 | (14.7%) | 1074 | (14.6%) |
| ≥25, <30 | 1306 | (35.4%) | 1327 | (36.1%) | 1339 | (36.3%) | 1294 | (35.3%) | 2633 | (35.8%) |
| ≥30<35 | 1037 | (28.1%) | 986 | (26.8%) | 1019 | (27.6%) | 1004 | (27.4%) | 2023 | (27.5%) |
| ≥35 | 698 | (18.9%) | 692 | (18.8%) | 675 | (18.3%) | 715 | (19.5%) | 1390 | (18.9%) |
| Unknown | 126 | (3.4%) | 114 | (3.1%) | 124 | (3.4%) | 116 | (3.2%) | 240 | (3.3%) |
|  |  |  |  |  |  |  |  |  |  |  |
|  |  |  |  |  |  |  |  |  |  |  |
| **Cigarette smoking** |  |  |  |  |  |  |  |  |  |  |
| Current | 281 | (7.6%) | 296 | (8.1%) | 294 | (8.0%) | 283 | (7.7%) | 577 | (7.8%) |
| Former | 1703 | (46.2%) | 1714 | (46.7%) | 1728 | (46.8%) | 1689 | (46.0%) | 3417 | (46.4%) |
| Never | 1656 | (44.9%) | 1620 | (44.1%) | 1629 | (44.1%) | 1647 | (44.9%) | 3276 | (44.5%) |
| Unknown | 47 | (1.3%) | 43 | (1.2%) | 41 | (1.1%) | 49 | (1.3%) | 90 | (1.2%) |
|  |  |  |  |  |  |  |  |  |  |  |
| **Non-study medication** |  |  |  |  |  |  |  |  |  |  |
| ACE-inhibitor or ARB | 2174 | (59.0%) | 2102 | (57.2%) | 2167 | (58.7%) | 2109 | (57.5%) | 4276 | (58.1%) |
| Aspirin use before screening | 1283 | (34.8%) | 1244 | (33.9%) | 1250 | (33.9%) | 1277 | (34.8%) | 2527 | (34.3%) |
| Thiazide or related diuretic | 676 | (18.3%) | 684 | (18.6%) | 673 | (18.2%) | 687 | (18.7%) | 1360 | (18.5%) |
| Calcium channel blocker | 924 | (25.1%) | 881 | (24.0%) | 923 | (25.0%) | 882 | (24.0%) | 1805 | (24.5%) |
| Statin | 2819 | (76.5%) | 2777 | (75.6%) | 2779 | (75.3%) | 2817 | (76.8%) | 5596 | (76.0%) |
|  |  |  |  |  |  |  |  |  |  |  |
| **Total cholesterol (mmol/L)** |  |  |  |  |  |  |  |  |  |  |
| Mean (SD) | 4.1±0.9 | | 4.1±0.8 | | 4.1±0.8 | | 4.1±0.9 | | 4.1±0.9 | |
| <4 | 1123 | (30.5%) | 1124 | (30.6%) | 1120 | (30.3%) | 1127 | (30.7%) | 2247 | (30.5%) |
| ≥4 <5 | 941 | (25.5%) | 937 | (25.5%) | 969 | (26.2%) | 909 | (24.8%) | 1878 | (25.5%) |
| ≥5 | 318 | (8.6%) | 331 | (9.0%) | 314 | (8.5%) | 335 | (9.1%) | 649 | (8.8%) |
| Not available | 1305 | (35.4%) | 1281 | (34.9%) | 1289 | (34.9%) | 1297 | (35.4%) | 2586 | (35.1%) |
|  |  |  |  |  |  |  |  |  |  |  |
| **HDL cholesterol (mmol/L)** |  |  |  |  |  |  |  |  |  |  |
| Mean (SD) | 1.3±0.4 | | 1.3±0.3 | | 1.3±0.4 | | 1.3±0.4 | | 1.3±0.4 | |
| <1 | 538 | (14.6%) | 474 | (12.9%) | 505 | (13.7%) | 507 | (13.8%) | 1012 | (13.8%) |
| ≥1<1.5 | 1318 | (35.7%) | 1415 | (38.5%) | 1373 | (37.2%) | 1360 | (37.1%) | 2733 | (37.1%) |
| ≥1.5 | 522 | (14.2%) | 496 | (13.5%) | 516 | (14.0%) | 502 | (13.7%) | 1018 | (13.8%) |
| Not available | 1309 | (35.5%) | 1288 | (35.1%) | 1298 | (35.2%) | 1299 | (35.4%) | 2597 | (35.3%) |
|  |  |  |  |  |  |  |  |  |  |  |
| **Non-HDL cholesterol (mmol/L)** |  |  |  |  |  |  |  |  |  |  |
| Mean (SD) | 2.9±0.8 | | 2.9±0.8 | | 2.9±0.8 | | 2.9±0.8 | | 2.9±0.8 | |
| <2.5 | 827 | (22.4%) | 845 | (23.0%) | 831 | (22.5%) | 841 | (22.9%) | 1672 | (22.7%) |
| ≥2.5 <3.5 | 1098 | (29.8%) | 1072 | (29.2%) | 1106 | (30.0%) | 1064 | (29.0%) | 2170 | (29.5%) |
| ≥3.5 | 453 | (12.3%) | 468 | (12.7%) | 457 | (12.4%) | 464 | (12.6%) | 921 | (12.5%) |
| Not available | 1309 | (35.5%) | 1288 | (35.1%) | 1298 | (35.2%) | 1299 | (35.4%) | 2597 | (35.3%) |
|  |  |  |  |  |  |  |  |  |  |  |
| **Glycosylated haemoglobin - HbA1c** |  |  |  |  |  |  |  |  |  |  |
| IFCC (mmol/mol) mean (SD) | 54.3±12.9 | | 54.3±12.5 | | 54.4±12.6 | | 54.2±12.7 | | 54.3±12.7 | |
| DCCT (%) mean (SD) | 7.1±1.2 | | 7.1±1.2 | | 7.1±1.2 | | 7.1±1.2 | | 7.1±1.2 | |
| <48 (6.5) | 841 | (22.8%) | 799 | (21.8%) | 807 | (21.9%) | 833 | (22.7%) | 1640 | (22.3%) |
| ≥48 (6.5), <64 (8.0) | 1123 | (30.5%) | 1157 | (31.5%) | 1172 | (31.7%) | 1108 | (30.2%) | 2280 | (31.0%) |
| ≥64 (8.0) | 418 | (11.3%) | 435 | (11.8%) | 424 | (11.5%) | 429 | (11.7%) | 853 | (11.6%) |
| Not available | 1305 | (35.4%) | 1282 | (34.9%) | 1289 | (34.9%) | 1298 | (35.4%) | 2587 | (35.1%) |
|  |  |  |  |  |  |  |  |  |  |  |
| **CKD-EPI estimated GFR**  **(ml/min/1.73m^2^)** § |  | |  |  |  |  |  |  |  |  |
| Mean (SD) | 85.0±20.6 | | 84.3±20.7 | | 84.7±20.7 | | 84.7±20.6 | | 84.7±20.6 | |
| ≥90 | 1082 | (29.3%) | 1047 | (28.5%) | 1058 | (28.7%) | 1071 | (29.2%) | 2129 | (28.9%) |
| ≥60<90 | 994 | (27.0%) | 1024 | (27.9%) | 1042 | (28.2%) | 976 | (26.6%) | 2018 | (27.4%) |
| <60 | 304 | (8.2%) | 320 | (8.7%) | 302 | (8.2%) | 322 | (8.8%) | 624 | (8.5%) |
| Not available | 1307 | (35.4%) | 1282 | (34.9%) | 1290 | (34.9%) | 1299 | (35.4%) | 2589 | (35.2%) |
|  |  |  |  |  |  |  |  |  |  |  |
| **Urinary albumin:creatinine ratio (mg/mmol)¶** |  |  |  |  |  |  |  |  |  |  |
| Median (IQR) | 0.56  (0.00-1.29) | | 0.54  (0.18-1.27) | | 0.55  (0.17-1.29) | | 0.54  (0.00-1.26) | | 0.54  (0.16-1.28) | |
| <3 | 2087 | (56.6%) | 2105 | (57.3%) | 2099 | (56.9%) | 2093 | (57.1%) | 4192 | (57.0%) |
| ≥3 | 290 | (7.9%) | 282 | (7.7%) | 300 | (8.1%) | 272 | (7.4%) | 572 | (7.8%) |
| Not available | 1310 | (35.5%) | 1286 | (35.0%) | 1293 | (35.0%) | 1303 | (35.5%) | 2596 | (35.3%) |
|  |  |  |  |  |  |  |  |  |  |  |
| **Townsend Deprivation Index**** |  |  |  |  |  |  |  |  |  |  |
| <-3 | 1217 | (33.0%) | 1289 | (35.1%) | 1262 | (34.2%) | 1244 | (33.9%) | 2506 | (34.0%) |
| ≥-3<0 | 1474 | (40.0%) | 1440 | (39.2%) | 1436 | (38.9%) | 1478 | (40.3%) | 2914 | (39.6%) |
| ≥0<2 | 502 | (13.6%) | 468 | (12.7%) | 497 | (13.5%) | 473 | (12.9%) | 970 | (13.2%) |
| ≥2<4 | 294 | (8.0%) | 275 | (7.5%) | 291 | (7.9%) | 278 | (7.6%) | 569 | (7.7%) |
| ≥4<6 | 141 | (3.8%) | 141 | (3.8%) | 140 | (3.8%) | 142 | (3.9%) | 282 | (3.8%) |
| ≥6 | 52 | (1.4%) | 54 | (1.5%) | 58 | (1.6%) | 48 | (1.3%) | 106 | (1.4%) |
| Unknown | 7 | (0.2%) | 6 | (0.2%) | 8 | (0.2%) | 5 | (0.1%) | 13 | (0.2%) |
|  |  |  |  |  |  |  |  |  |  |  |
| **Ethnic origin** |  |  |  |  |  |  |  |  |  |  |
| White | 3553 | (96.4%) | 3530 | (96.1%) | 3554 | (96.3%) | 3529 | (96.2%) | 7083 | (96.2%) |
| Indian/Pakistani/Bangladeshi | 50 | (1.4%) | 50 | (1.4%) | 51 | (1.4%) | 49 | (1.3%) | 100 | (1.4%) |
| African/Caribbean | 26 | (0.7%) | 35 | (1.0%) | 28 | (0.8%) | 33 | (0.9%) | 61 | (0.8%) |
| Other/unknown | 58 | (1.6%) | 58 | (1.6%) | 59 | (1.6%) | 57 | (1.6%) | 116 | (1.6%) |
|  |  |  |  |  |  |  |  |  |  |  |

DCCT = Diabetes Control and Complications Trial; FAs=Fatty acids; GFR = Glomerular Filtration Rate; HDL=High-density lipoprotein; IFCC =International Federation of Clinical Chemistry; IQR = Interquartile range; SD = Standard Deviation

Figures presented are counts with percentages unless otherwise stated. Percentages may not total 100 because of rounding.

*The presence of type 2 diabetes was based on a broad clinical definition involving the participant’s age at the diagnosis of diabetes, the use of insulin within one year after diagnosis, and the body-mass index.

† From blood and urine consent forms, generally before randomization.

‡ The body-mass index (the weight in kilograms divided by the square of the height in metres) was based on values for height and weight the participants reported on their randomization questionnaires.

§ Calculated from blood cystatin c concentration using the CKD-EPI formula.^381^

¶There was an analysis rule in ASCEND which stated that those with a below detectable threshold albumin component of their urinary albumin creatinine ratio would be recorded as zero. This applied to just over 25% of participants in the active group of the aspirin arm, and to just under 25% of participants in the placebo group. Hence the interquartile range included zero.

**Townsend scores of 0 represent an area with a UK-average level of deprivation, positive values indicate areas of above-average deprivation, and negative values indicate areas of below-average deprivation.

## Table S3 Heterogeneity of baseline characteristics between those included and excluded from the DESP-linked cohort (B1)

| **Baseline Characteristic** | **DESP-Linkage Exercise** | | | | | **P Values*** |
| --- | --- | --- | --- | --- | --- | --- |
|  | **Included**  **(n=7360)** | | **Excluded**  **(n=8120)** | | |  |
|  |  |  | |  |  |  |
| **Age at randomization (years)** |  |  | |  |  |  |
| Mean (SD) | 63.5±8.9 | | | 63.0±9.4 | | P = 0.002 |
| <60 | 2574 | (35.0%) | | 3016 | (37.1%) |  |
| ≥60<70 | 3047 | (41.4%) | | 3200 | (39.4%) |  |
| ≥70 | 1739 | (23.6%) | | 1904 | (23.4%) | P = 0.01 |
|  |  |  | |  |  |  |
| **Sex** |  |  | |  |  |  |
| Male | 4594 | (62.4%) | | 5090 | (62.7%) |  |
| Female | 2766 | (37.6%) | | 3030 | (37.3%) | P = 0.75 |
|  |  |  | |  |  |  |
| **Type of diabetes** |  |  | |  |  |  |
| Type 1 | 373 | (5.1%) | | 538 | (6.6%) |  |
| Type 2 | 6987 | (94.9%) | | 7582 | (93.4%) | P<0.001 |
|  |  |  | |  |  |  |
| **Duration of diabetes (years)** |  |  | |  |  |  |
| Median (IQR) | 6 (3-12) | | | 7 (3-12) | | P<0.001 |
| ≥0<5 years | 2387 | (32.4%) | | 2504 | (30.8%) |  |
| ≥5<10 years | 2102 | (28.6%) | | 2232 | (27.5%) |  |
| ≥10<20 years | 1681 | (22.8%) | | 1856 | (22.9%) |  |
| ≥20 years | 778 | (10.6%) | | 1084 | (13.3%) | P<0.001 |
| Unknown | 412 | (5.6%) | | 444 | (5.5%) |  |
|  |  |  | |  |  |  |
| **Diabetes management** |  |  | |  |  |  |
| Diet only | 1212 | (16.5%) | | 1317 | (16.2%) |  |
| Oral hypoglycaemic agent(s) only | 4472 | (60.8%) | | 4548 | (56.0%) |  |
| Insulin +/- oral hypoglycaemic agent(s) | 1676 | (22.8%) | | 2255 | (27.8%) | P<0.001 |
|  |  |  | |  |  |  |
| **Participant-reported diabetic retinopathy** |  |  | |  |  |  |
| Yes | 1308 | (17.8%) | | 1715 | (21.1%) |  |
| No | 5991 | (81.4%) | | 6322 | (77.9%) | P<0.001 |
| Unknown | 61 | (0.8%) | | 83 | (1.0%) |  |
|  |  |  | |  |  |  |
| **Participant-reported treatment for hypertension** |  |  | |  |  |  |
| Yes | 4502 | (61.2%) | | 5031 | (62.0%) |  |
| No | 2798 | (38.0%) | | 3037 | (37.4%) | P = 0.39 |
| Unknown | 60 | (0.8%) | | 52 | (0.6%) |  |
|  |  |  | |  |  |  |
| **Systolic blood pressure**  **(mmHg)** |  |  | |  |  |  |
| Mean (SD) | 136.1±15.2 | | | 136.3±15.3 | | P = 0.49 |
| <130 | 1658 | (22.5%) | | 1736 | (21.4%) |  |
| ≥130<140 | 1504 | (20.4%) | | 1587 | (19.5%) |  |
| ≥140 | 2224 | (30.2%) | | 2331 | (28.7%) | P = 0.97 |
| Unknown | 1974 | (26.8%) | | 2466 | (30.4%) |  |
|  |  |  | |  |  |  |
| **Diastolic blood pressure**  **(mmHg)** |  |  | |  |  |  |
| Mean (SD) | 77.1±9.4 | | | 77.1±9.5 | | P = 0.93 |
| <75 | 2036 | (27.7%) | | 2187 | (26.9%) |  |
| ≥75 <85 | 2297 | (31.2%) | | 2322 | (28.6%) |  |
| ≥85 | 1049 | (14.3%) | | 1142 | (14.1%) | P = 0.23 |
| Unknown | 1978 | (26.9%) | | 2469 | (30.4%) |  |
|  |  |  | |  |  |  |
| **Body mass index**  **(kg/m^2^)** |  |  | |  |  |  |
| Mean (SD) | 30.7±6.2 | | | 30.8±6.4 | | P = 0.42 |
| <25 | 1074 | (14.6%) | | 1175 | (14.5%) |  |
| ≥25, <30 | 2633 | (35.8%) | | 2896 | (35.7%) |  |
| ≥30<35 | 2023 | (27.5%) | | 2217 | (27.3%) |  |
| ≥35 | 1390 | (18.9%) | | 1571 | (19.3%) | P = 0.91 |
| Unknown | 240 | (3.3%) | | 261 | (3.2%) |  |
|  |  |  | |  |  |  |
| **Cigarette smoking** |  |  | |  |  |  |
| Current | 577 | (7.8%) | | 702 | (8.6%) |  |
| Former | 3417 | (46.4%) | | 3634 | (44.8%) |  |
| Never | 3276 | (44.5%) | | 3701 | (45.6%) | P = 0.04 |
| Unknown | 90 | (1.2%) | | 83 | (1.0%) |  |
|  |  |  | |  |  |  |
| **Non-study medication** |  |  | |  |  |  |
| ACE inhibitor or ARB | 4276 | (58.1%) | | 4779 | (58.9%) | P = 0.35 |
| Aspirin use before screening | 2527 | (34.3%) | | 2981 | (36.7%) | P = 0.002 |
| Thiazide or related diuretic | 1360 | (18.5%) | | 1597 | (19.7%) | P = 0.06 |
| Calcium channel blocker | 1805 | (24.5%) | | 1968 | (24.2%) | P = 0.69 |
| Statin | 5596 | (76.0%) | | 6057 | (74.6%) | P = 0.04 |
|  |  |  | |  |  |  |
| **Total cholesterol**  **(mmol/L)** |  |  | |  |  |  |
| Mean (SD) | 4.1±0.9 | | | 4.2±0.9 | | P = 0.005 |
| <4 | 2247 | (30.5%) | | 2307 | (28.4%) |  |
| ≥4 <5 | 1878 | (25.5%) | | 1917 | (23.6%) |  |
| ≥5 | 649 | (8.8%) | | 821 | (10.1%) | P = 0.23 |
| Not available | 2586 | (35.1%) | | 3075 | (37.9%) |  |
|  |  |  | |  |  |  |
| **HDL cholesterol**  **(mmol/L)** |  |  | |  |  |  |
| Mean (SD) | 1.3±0.4 | | | 1.3±0.4 | | P = 0.78 |
| <1 | 1012 | (13.8%) | | 1158 | (14.3%) |  |
| ≥1<1.5 | 2733 | (37.1%) | | 2790 | (34.4%) |  |
| ≥1.5 | 1018 | (13.8%) | | 1089 | (13.4%) | P = 0.08 |
| Not available | 2597 | (35.3%) | | 3083 | (38.0%) |  |
|  |  |  | |  |  |  |
| **Non-HDL cholesterol**  **(mmol/L)** |  |  | |  |  |  |
| Mean (SD) | 2.9±0.8 | | | 2.9±0.9 | | P = 0.002 |
| <2.5 | 1672 | (22.7%) | | 1718 | (21.2%) |  |
| ≥2.5 <3.5 | 2170 | (29.5%) | | 2223 | (27.4%) |  |
| ≥3.5 | 921 | (12.5%) | | 1096 | (13.5%) | P = 0.01 |
| Not available | 2597 | (35.3%) | | 3083 | (38.0%) |  |
|  |  |  | |  |  |  |
| **Glycosylated haemoglobin - HbA1c** |  |  | |  |  |  |
| IFCC (mmol/mol) mean (SD) | 54.3±12.7 | | | 55.3±13.1 | | P<0.001 |
| DCCT (%) mean (SD) | 7.1±1.2 | | | 7.2±1.2 | | P<0.001 |
| <48 (6.5) | 1640 | (22.3%) | | 1632 | (20.1%) |  |
| ≥48 (6.5), <64 (8.0) | 2280 | (31.0%) | | 2384 | (29.4%) |  |
| ≥64 (8.0) | 853 | (11.6%) | | 1024 | (12.6%) | P = 0.005 |
| Not available | 2587 | (35.1%) | | 3080 | (37.9%) |  |
|  |  |  | |  |  |  |
| **CKD-EPI estimated GFR**  **(ml/min/1.73m^2^)** |  |  | |  |  |  |
| Mean (SD) | 84.7±20.6 | | | 85.7±21.4 | | P = 0.02 |
| ≥90 | 2129 | (28.9%) | | 2394 | (29.5%) |  |
| ≥60<90 | 2018 | (27.4%) | | 1998 | (24.6%) |  |
| <60 | 624 | (8.5%) | | 652 | (8.0%) | P = 0.01 |
| Not available | 2589 | (35.2%) | | 3076 | (37.9%) |  |
|  |  |  | |  |  |  |
| **Urinary albumin:creatinine ratio**  **(mg/mmol)** |  |  | |  |  |  |
| Median (IQR) | 0.54 (0.16-1.28) | | | 0.56 (0.17-1.41) | | P<0.001 |
| <3 | 4192 | (57.0%) | | 4334 | (53.4%) |  |
| ≥3 | 572 | (7.8%) | | 676 | (8.3%) | P = 0.03 |
| Not available | 2596 | (35.3%) | | 3110 | (38.3%) |  |
|  |  |  | |  |  |  |
| **Townsend Deprivation Index** |  |  | |  |  |  |
| <-3 | 2506 | (34.0%) | | 2598 | (32.0%) |  |
| ≥-3<0 | 2914 | (39.6%) | | 3108 | (38.3%) |  |
| ≥0<2 | 970 | (13.2%) | | 1067 | (13.1%) |  |
| ≥2<4 | 569 | (7.7%) | | 746 | (9.2%) |  |
| ≥4<6 | 282 | (3.8%) | | 421 | (5.2%) |  |
| ≥6 | 106 | (1.4%) | | 155 | (1.9%) | P<0.001 |
| Unknown | 13 | (0.2%) | | 25 | (0.3%) |  |
|  |  |  | |  |  |  |
| **Ethnic origin** |  |  | |  |  |  |
| White | 7083 | (96.2%) | | 7852 | (96.7%) |  |
| Indian/Pakistani/Bangladeshi | 100 | (1.4%) | | 84 | (1.0%) |  |
| African/Caribbean | 61 | (0.8%) | | 79 | (1.0%) |  |
| Other/unknown | 116 | (1.6%) | | 105 | (1.3%) | P = 0.09 |

DCCT = Diabetes Control and Complications Trial; FAs = Fatty acids; GFR = Glomerular Filtration Rate; HDL=High-density lipoprotein; IFCC =International Federation of Clinical Chemistry; IQR = Interquartile range; SD = Standard Deviation

Figures presented are counts with percentages unless otherwise stated.

*Formal testing for differences in baseline characteristics between those included in and excluded from the analysis cohort used the Chi-squared test for heterogeneity for categorical variables and grouped continuous variables (including age, duration of diabetes, systolic blood pressure, diastolic blood pressure, BMI, total cholesterol, HDL-cholesterol, non-HDL cholesterol, HbA1c, eGFR and urinary albumin:creatinine ratio), the Mantel-Haenszel Chi-square test for trend for ordinal variables (i.e. Townsend Deprivation Index), and the t-test or Wilcoxon-Mann-Whitney U test for continuous variables with a normal (summarised by mean±SD) or non-normal (summarised by median and interquartile range) distribution respectively. Categories that were “unknown” or “not available” were excluded.

Percentages may not total 100 because of rounding

## Table S4 Baseline Characteristics of the VFQ Responders (Cohort C)

| **Baseline Characteristic** | **Aspirin** | | | | **Omega-3 FAs** | | | | **Overall**  **(n=8846)** | |
| --- | --- | --- | --- | --- | --- | --- | --- | --- | --- | --- |
|  | **Active**  **(n=4447)** | | **Placebo**  **(n=4399)** | | **Active**  **(n=4417)** | | **Placebo**  **(n=4429)** | |  |  |
|  |  |  |  |  |  |  |  |  |  |  |
| **Age at randomization**  **(years)** |  |  |  |  |  |  |  |  |  |  |
| Mean (SD) | 62.4±8.3 | | 62.5±8.3 | | 62.4±8.3 | | 62.5±8.3 | | 62.5±8.3 | |
| <60 | 1679 | (37.8%) | 1660 | (37.7%) | 1673 | (37.9%) | 1666 | (37.6%) | 3339 | (37.7%) |
| ≥60<70 | 1938 | (43.6%) | 1922 | (43.7%) | 1916 | (43.4%) | 1944 | (43.9%) | 3860 | (43.6%) |
| ≥70 | 830 | (18.7%) | 817 | (18.6%) | 828 | (18.7%) | 819 | (18.5%) | 1647 | (18.6%) |
|  |  |  |  |  |  |  |  |  |  |  |
| **Sex** |  |  |  |  |  |  |  |  |  |  |
| Male | 2785 | (62.6%) | 2744 | (62.4%) | 2758 | (62.4%) | 2771 | (62.6%) | 5529 | (62.5%) |
| Female | 1662 | (37.4%) | 1655 | (37.6%) | 1659 | (37.6%) | 1658 | (37.4%) | 3317 | (37.5%) |
|  |  |  |  |  |  |  |  |  |  |  |
| **Type of diabetes*** |  |  |  |  |  |  |  |  |  |  |
| Type 1 | 270 | (6.1%) | 289 | (6.6%) | 288 | (6.5%) | 271 | (6.1%) | 559 | (6.3%) |
| Type 2 | 4177 | (93.9%) | 4110 | (93.4%) | 4129 | (93.5%) | 4158 | (93.9%) | 8287 | (93.7%) |
|  |  |  |  |  |  |  |  |  |  |  |
| **Duration of diabetes**  **(years)** |  |  |  |  |  |  |  |  |  |  |
| Median (IQR) | 7 (3-12) | | 6 (3-12) | | 7 (3-12) | | 6 (3-12) | | 7(3-12) | |
| ≥0<5 years | 1459 | (32.8%) | 1459 | (33.2%) | 1436 | (32.5%) | 1482 | (33.5%) | 2918 | (33.0%) |
| ≥5<10 years | 1305 | (29.3%) | 1239 | (28.2%) | 1273 | (28.8%) | 1271 | (28.7%) | 2544 | (28.8%) |
| ≥10<20 years | 966 | (21.7%) | 986 | (22.4%) | 986 | (22.3%) | 966 | (21.8%) | 1952 | (22.1%) |
| ≥20 years | 499 | (11.2%) | 521 | (11.8%) | 515 | (11.7%) | 505 | (11.4%) | 1020 | (11.5%) |
| Unknown | 218 | (4.9%) | 194 | (4.4%) | 207 | (4.7%) | 205 | (4.6%) | 412 | (4.7%) |
|  |  |  |  |  |  |  |  |  |  |  |
| **Diabetes management** |  |  |  |  |  |  |  |  |  |  |
| Diet only | 766 | (17.2%) | 742 | (16.9%) | 728 | (16.5%) | 780 | (17.6%) | 1508 | (17.0%) |
| Oral hypoglycaemic agent(s) only | 2585 | (58.1%) | 2581 | (58.7%) | 2577 | (58.3%) | 2589 | (58.5%) | 5166 | (58.4%) |
| Insulin +/- oral hypoglycaemic agent(s) | 1096 | (24.6%) | 1076 | (24.5%) | 1112 | (25.2%) | 1060 | (23.9%) | 2172 | (24.6%) |
|  |  |  |  |  |  |  |  |  |  |  |
| **Participant-reported diabetic retinopathy** |  |  |  |  |  |  |  |  |  |  |
| Yes | 824 | (18.5%) | 804 | (18.3%) | 851 | (19.3%) | 777 | (17.5%) | 1628 | (18.4%) |
| No | 3590 | (80.7%) | 3561 | (81.0%) | 3537 | (80.1%) | 3614 | (81.6%) | 7151 | (80.8%) |
| Unknown | 33 | (0.7%) | 34 | (0.8%) | 29 | (0.7%) | 38 | (0.9%) | 67 | (0.8%) |
|  |  |  |  |  |  |  |  |  |  |  |
| **Participant-reported treatment for hypertension** |  |  |  |  |  |  |  |  |  |  |
| Yes | 2690 | (60.5%) | 2696 | (61.3%) | 2705 | (61.2%) | 2681 | (60.5%) | 5386 | (60.9%) |
| No | 1732 | (38.9%) | 1673 | (38.0%) | 1689 | (38.2%) | 1716 | (38.7%) | 3405 | (38.5%) |
| Unknown | 25 | (0.6%) | 30 | (0.7%) | 23 | (0.5%) | 32 | (0.7%) | 55 | (0.6%) |
|  |  |  |  |  |  |  |  |  |  |  |
| **Systolic blood pressure**  **(mmHg)** † |  |  |  |  |  |  |  |  |  |  |
| Mean (SD) | 135.7±15.1 | | 135.9±14.9 | | 135.9±15.1 | | 135.7±14.9 | | 135.8±14.9 | |
| <130 | 1022 | (23.0%) | 1001 | (22.8%) | 999 | (22.6%) | 1024 | (23.1%) | 2023 | (22.9%) |
| ≥130<140 | 899 | (20.2%) | 923 | (21.0%) | 908 | (20.6%) | 914 | (20.6%) | 1822 | (20.6%) |
| ≥140 | 1301 | (29.3%) | 1325 | (30.1%) | 1311 | (29.7%) | 1315 | (29.7%) | 2626 | (29.7%) |
| Unknown | 1225 | (27.5%) | 1150 | (26.1%) | 1199 | (27.1%) | 1176 | (26.6%) | 2375 | (26.8%) |
|  |  |  |  |  |  |  |  |  |  |  |
| **Diastolic blood pressure**  **(mmHg)** † |  |  |  |  |  |  |  |  |  |  |
| Mean (SD) | 77.3±9.3 | | 77.6±9.1 | | 77.5±9.2 | | 77.4±9.1 | | 77.4±9.2 | |
| <75 | 1192 | (26.8%) | 1179 | (26.8%) | 1161 | (26.3%) | 1210 | (27.3%) | 2371 | (26.8%) |
| ≥75 <85 | 1389 | (31.2%) | 1399 | (31.8%) | 1398 | (31.7%) | 1390 | (31.4%) | 2788 | (31.5%) |
| ≥85 | 639 | (14.4%) | 672 | (15.3%) | 658 | (14.9%) | 653 | (14.7%) | 1311 | (14.8%) |
| Unknown | 1227 | (27.6%) | 1149 | (26.1%) | 1200 | (27.2%) | 1176 | (26.6%) | 2376 | (26.9%) |
|  |  |  |  |  |  |  |  |  |  |  |
| **Body mass index**  **(kg/m^2^)** ‡ |  |  |  |  |  |  |  |  |  |  |
| Mean (SD) | 30.8±6.3 | | 30.4±5.9 | | 30.6±6.1 | | 30.6±6.1 | | 30.6±6.1 | |
| <25 | 636 | (14.3%) | 677 | (15.4%) | 638 | (14.4%) | 675 | (15.2%) | 1313 | (14.8%) |
| ≥25, <30 | 1604 | (36.1%) | 1626 | (37.0%) | 1616 | (36.6%) | 1614 | (36.4%) | 3230 | (36.5%) |
| ≥30<35 | 1223 | (27.5%) | 1206 | (27.4%) | 1222 | (27.7%) | 1207 | (27.3%) | 2429 | (27.5%) |
| ≥35 | 859 | (19.3%) | 760 | (17.3%) | 810 | (18.3%) | 809 | (18.3%) | 1619 | (18.3%) |
| Unknown | 125 | (2.8%) | 130 | (3.0%) | 131 | (3.0%) | 124 | (2.8%) | 255 | (2.9%) |
|  |  |  |  |  |  |  |  |  |  |  |
|  |  |  |  |  |  |  |  |  |  |  |
|  |  |  |  |  |  |  |  |  |  |  |
| **Cigarette smoking** |  |  |  |  |  |  |  |  |  |  |
| Current | 264 | (5.9%) | 291 | (6.6%) | 278 | (6.3%) | 277 | (6.3%) | 555 | (6.3%) |
| Former | 1995 | (44.9%) | 1952 | (44.4%) | 1959 | (44.4%) | 1988 | (44.9%) | 3947 | (44.6%) |
| Never | 2139 | (48.1%) | 2104 | (47.8%) | 2131 | (48.2%) | 2112 | (47.7%) | 4243 | (48.0%) |
| Unknown | 49 | (1.1%) | 52 | (1.2%) | 49 | (1.1%) | 52 | (1.2%) | 101 | (1.1%) |
|  |  |  |  |  |  |  |  |  |  |  |
| **Non-study medication** |  |  |  |  |  |  |  |  |  |  |
| ACE-inhibitor or ARB | 2559 | (57.5%) | 2556 | (58.1%) | 2597 | (58.8%) | 2518 | (56.9%) | 5115 | (57.8%) |
| Aspirin use before screening | 1598 | (35.9%) | 1559 | (35.4%) | 1582 | (35.8%) | 1575 | (35.6%) | 3157 | (35.7%) |
| Thiazide or related diuretic | 818 | (18.4%) | 821 | (18.7%) | 805 | (18.2%) | 834 | (18.8%) | 1639 | (18.5%) |
| Calcium channel blocker | 1074 | (24.2%) | 1014 | (23.1%) | 1040 | (23.5%) | 1048 | (23.7%) | 2088 | (23.6%) |
| Statin | 3424 | (77.0%) | 3345 | (76.0%) | 3356 | (76.0%) | 3413 | (77.1%) | 6769 | (76.5%) |
|  |  |  |  |  |  |  |  |  |  |  |
| **Total cholesterol (mmol/L)** |  |  |  |  |  |  |  |  |  |  |
| Mean (SD) | 4.1±0.9 | | 4.2±0.9 | | 4.2±0.9 | | 4.1±0.8 | | 4.1±0.9 | |
| <4 | 1360 | (30.6%) | 1321 | (30.0%) | 1321 | (29.9%) | 1360 | (30.7%) | 2681 | (30.3%) |
| ≥4 <5 | 1115 | (25.1%) | 1131 | (25.7%) | 1112 | (25.2%) | 1134 | (25.6%) | 2246 | (25.4%) |
| ≥5 | 398 | (8.9%) | 419 | (9.5%) | 413 | (9.4%) | 404 | (9.1%) | 817 | (9.2%) |
| Not available | 1574 | (35.4%) | 1528 | (34.7%) | 1571 | (35.6%) | 1531 | (34.6%) | 3102 | (35.1%) |
|  |  |  |  |  |  |  |  |  |  |  |
| **HDL cholesterol (mmol/L)** |  |  |  |  |  |  |  |  |  |  |
| Mean (SD) | 1.3±0.4 | | 1.3±0.4 | | 1.3±0.4 | | 1.3±0.4 | | 1.3±0.4 | |
| <1 | 632 | (14.2%) | 590 | (13.4%) | 612 | (13.9%) | 610 | (13.8%) | 1222 | (13.8%) |
| ≥1<1.5 | 1576 | (35.4%) | 1626 | (37.0%) | 1581 | (35.8%) | 1621 | (36.6%) | 3202 | (36.2%) |
| ≥1.5 | 662 | (14.9%) | 648 | (14.7%) | 646 | (14.6%) | 664 | (15.0%) | 1310 | (14.8%) |
| Not available | 1577 | (35.5%) | 1535 | (34.9%) | 1578 | (35.7%) | 1534 | (34.6%) | 3112 | (35.2%) |
|  |  |  |  |  |  |  |  |  |  |  |
| **Non-HDL cholesterol (mmol/L)** |  |  |  |  |  |  |  |  |  |  |
| Mean (SD) | 2.9±0.8 | | 2.9±0.8 | | 2.9±0.9 | | 2.9±0.8 | | 2.9±0.8 | |
| <2.5 | 1032 | (23.2%) | 1021 | (23.2%) | 1006 | (22.8%) | 1047 | (23.6%) | 2053 | (23.2%) |
| ≥2.5 <3.5 | 1288 | (29.0%) | 1270 | (28.9%) | 1254 | (28.4%) | 1304 | (29.4%) | 2558 | (28.9%) |
| ≥3.5 | 550 | (12.4%) | 573 | (13.0%) | 579 | (13.1%) | 544 | (12.3%) | 1123 | (12.7%) |
| Not available | 1577 | (35.5%) | 1535 | (34.9%) | 1578 | (35.7%) | 1534 | (34.6%) | 3112 | (35.2%) |
|  |  |  |  |  |  |  |  |  |  |  |
| **Glycosylated haemoglobin - HbA1c** |  |  |  |  |  |  |  |  |  |  |
| IFCC (mmol/mol) mean (SD) | 53.9±12.1 | | 54.2±12.2 | | 54.2±12.1 | | 53.9±12.3 | | 54.0±12.2 | |
| DCCT (%) mean (SD) | 7.1±1.1 | | 7.1±1.1 | | 7.1±1.1 | | 7.1±1.1 | | 7.1±1.1 | |
| <48 (6.5) | 1011 | (22.7%) | 946 | (21.5%) | 963 | (21.8%) | 994 | (22.4%) | 1957 | (22.1%) |
| ≥48 (6.5), <64 (8.0) | 1363 | (30.6%) | 1429 | (32.5%) | 1376 | (31.2%) | 1416 | (32.0%) | 2792 | (31.6%) |
| ≥64 (8.0) | 496 | (11.2%) | 494 | (11.2%) | 503 | (11.4%) | 487 | (11.0%) | 990 | (11.2%) |
| Not available | 1577 | (35.5%) | 1530 | (34.8%) | 1575 | (35.7%) | 1532 | (34.6%) | 3107 | (35.1%) |
|  |  |  |  |  |  |  |  |  |  |  |
| **CKD-EPI estimated GFR**  **(ml/min/1.73m^2^)** § |  |  |  |  |  |  |  |  |  |  |
| Mean (SD) | 87.9±19.6 | | 87.7±19.9 | | 87.7±20.2 | | 87.8±19.3 | | 87.8±19.8 | |
| ≥90 | 1459 | (32.8%) | 1443 | (32.8%) | 1429 | (32.4%) | 1473 | (33.3%) | 2902 | (32.8%) |
| ≥60<90 | 1139 | (25.6%) | 1160 | (26.4%) | 1148 | (26.0%) | 1151 | (26.0%) | 2299 | (26.0%) |
| <60 | 273 | (6.1%) | 267 | (6.1%) | 268 | (6.1%) | 272 | (6.1%) | 540 | (6.1%) |
| Not available | 1576 | (35.4%) | 1529 | (34.8%) | 1572 | (35.6%) | 1533 | (34.6%) | 3105 | (35.1%) |
|  |  |  |  |  |  |  |  |  |  |  |
| **Urinary albumin:creatinine ratio (mg/mmol)** ¶ |  |  |  |  |  |  |  |  |  |  |
| Median (IQR) | 0.50  (0.00-1.16) | | 0.49  (0.00-1.12) | | 0.50  (0.00-1.17) | | 0.50  (0.00-1.12) | | 0.50  (0.00-1.14) | |
| <3 | 2571 | (57.8%) | 2549 | (57.9%) | 2520 | (57.1%) | 2600 | (58.7%) | 5120 | (57.9%) |
| ≥3 | 303 | (6.8%) | 310 | (7.0%) | 323 | (7.3%) | 290 | (6.5%) | 613 | (6.9%) |
| Not available | 1573 | (35.4%) | 1540 | (35.0%) | 1574 | (35.6%) | 1539 | (34.7%) | 3113 | (35.2%) |
|  |  |  |  |  |  |  |  |  |  |  |
| **Townsend Deprivation Index**** |  |  |  |  |  |  |  |  |  |  |
| <-3 | 1537 | (34.6%) | 1564 | (35.6%) | 1574 | (35.6%) | 1527 | (34.5%) | 3101 | (35.1%) |
| ≥-3<0 | 1830 | (41.2%) | 1758 | (40.0%) | 1762 | (39.9%) | 1826 | (41.2%) | 3588 | (40.6%) |
| ≥0<2 | 537 | (12.1%) | 534 | (12.1%) | 555 | (12.6%) | 516 | (11.7%) | 1071 | (12.1%) |
| ≥2<4 | 322 | (7.2%) | 303 | (6.9%) | 292 | (6.6%) | 333 | (7.5%) | 625 | (7.1%) |
| ≥4<6 | 161 | (3.6%) | 162 | (3.7%) | 162 | (3.7%) | 161 | (3.6%) | 323 | (3.7%) |
| ≥6 | 52 | (1.2%) | 66 | (1.5%) | 64 | (1.4%) | 54 | (1.2%) | 118 | (1.3%) |
| Unknown | 8 | (0.2%) | 12 | (0.3%) | 8 | (0.2%) | 12 | (0.3%) | 20 | (0.2%) |
|  |  |  |  |  |  |  |  |  |  |  |
| **Ethnic origin** |  |  |  |  |  |  |  |  |  |  |
| White | 4309 | (96.9%) | 4255 | (96.7%) | 4279 | (96.9%) | 4285 | (96.7%) | 8564 | (96.8%) |
| Indian/Pakistani/Bangladeshi | 53 | (1.2%) | 54 | (1.2%) | 53 | (1.2%) | 54 | (1.2%) | 107 | (1.2%) |
| African/Caribbean | 26 | (0.6%) | 28 | (0.6%) | 22 | (0.5%) | 32 | (0.7%) | 54 | (0.6%) |
| Other/unknown | 59 | (1.3%) | 62 | (1.4%) | 63 | (1.4%) | 58 | (1.3%) | 121 | (1.4%) |
|  |  |  |  |  |  |  |  |  |  |  |

DCCT = Diabetes Control and Complications Trial; GFR = Glomerular Filtration Rate; FAs=Fatty acids, HDL=High-density lipoprotein; IFCC =International Federation of Clinical Chemistry; IQR = Interquartile range; SD = Standard Deviation

Figures presented are counts with percentages unless otherwise stated. Percentages may not total 100 because of rounding.

*The presence of type 2 diabetes was based on a broad clinical definition involving the participant’s age at the diagnosis of diabetes, the use of insulin within one year after diagnosis, and the body-mass index.

† From blood and urine consent forms, generally before randomization.

‡ The body-mass index (the weight in kilograms divided by the square of the height in metres) was based on values for height and weight the participants reported on their randomization questionnaires.

§ Calculated from blood cystatin c concentration using the CKD-EPI formula.^381^

¶There was an analysis rule in ASCEND which stated that those with a below detectable threshold albumin component of their urinary albumin creatinine ratio, would be recorded as zero. This applied to just over 25% of participants in the active group of the aspirin arm, and to just under 25% of participants in the placebo group. Hence the interquartile range included zero.

**Townsend scores of 0 represent an area with a UK-average level of deprivation, positive values indicate areas of above-average deprivation, and negative values indicate areas of below-average deprivation.

## Table S5 Heterogeneity of baseline characteristics between those included and excluded from the VFQ cohort

| **Baseline Characteristic** | **VFQ Exercise** | | | | **P Values*** |
| --- | --- | --- | --- | --- | --- |
|  | **Included (8846)** | | **Excluded* (n=6634)** | |  |
|  |  |  |  |  |  |
| **Age at randomization (years)** |  |  |  |  |  |
| Mean (SD) | 62.5±8.3 | | 64.3±10.2 | | P<0.001 |
| <60 | 3339 | (37.7%) | 2251 | (33.9%) |  |
| ≥60<70 | 3860 | (43.6%) | 2387 | (36.0%) |  |
| ≥70 | 1647 | (18.6%) | 1996 | (30.1%) | P<0.001 |
|  |  |  |  |  |  |
| **Sex** |  |  |  |  |  |
| Male | 5529 | (62.5%) | 4155 | (62.6%) |  |
| Female | 3317 | (37.5%) | 2479 | (37.4%) | P = 0.88 |
|  |  |  |  |  |  |
| **Type of diabetes** |  |  |  |  |  |
| Type 1 | 559 | (6.3%) | 352 | (5.3%) |  |
| Type 2 | 8287 | (93.7%) | 6282 | (94.7%) | P = 0.009 |
|  |  |  |  |  |  |
| **Duration of diabetes (years)** |  |  |  |  |  |
| Median (IQR) | 7(3-12) | | 7 (4-13) | | P<0.001 |
| ≥0<5 years | 2918 | (33.0%) | 1973 | (29.7%) |  |
| ≥5<10 years | 2544 | (28.8%) | 1790 | (27.0%) |  |
| ≥10<20 years | 1952 | (22.1%) | 1585 | (23.9%) |  |
| ≥20 years | 1020 | (11.5%) | 842 | (12.7%) | P<0.001 |
| Unknown | 412 | (4.7%) | 444 | (6.7%) |  |
|  |  |  |  |  |  |
| **Diabetes management** |  |  |  |  |  |
| Diet only | 1508 | (17.0%) | 1021 | (15.4%) |  |
| Oral hypoglycaemic agent(s) only | 5166 | (58.4%) | 3854 | (58.1%) |  |
| Insulin+/- oral hypoglycaemic agents | 2172 | (24.6%) | 1759 | (26.5%) | P = 0.002 |
|  |  |  |  |  |  |
| **Participant-reported diabetic retinopathy** |  |  |  |  |  |
| Yes | 1628 | (18.4%) | 1395 | (21.0%) |  |
| No | 7151 | (80.8%) | 5162 | (77.8%) | P<0.001 |
| Unknown | 67 | (0.8%) | 77 | (1.2%) |  |
|  |  |  |  |  |  |
| **Participant-reported treatment for hypertension** |  |  |  |  |  |
| Yes | 5386 | (60.9%) | 4147 | (62.5%) |  |
| No | 3405 | (38.5%) | 2430 | (36.6%) | P = 0.03 |
| Unknown | 55 | (0.6%) | 57 | (0.9%) |  |
|  |  |  |  |  |  |
| **Systolic blood pressure**  **(mmHg)** |  |  |  |  |  |
| Mean (SD) | 135.8±14.9 | | 136.6±15.7 | | P = 0.005 |
| <130 | 2023 | (22.9%) | 1371 | (20.7%) |  |
| ≥130<140 | 1822 | (20.6%) | 1269 | (19.1%) |  |
| ≥140 | 2626 | (29.7%) | 1929 | (29.1%) | P = 0.14 |
| Unknown | 2375 | (26.8%) | 2065 | (31.1%) |  |
|  |  |  |  |  |  |
| **Diastolic blood pressure**  **(mmHg)** |  |  |  |  |  |
| Mean (SD) | 77.4±9.2 | | 76.6±9.8 | | P<0.001 |
| <75 | 2371 | (26.8%) | 1852 | (27.9%) |  |
| ≥75 <85 | 2788 | (31.5%) | 1831 | (27.6%) |  |
| ≥85 | 1311 | (14.8%) | 880 | (13.3%) | P<0.001 |
| Unknown | 2376 | (26.9%) | 2071 | (31.2%) |  |
|  |  |  |  |  |  |
| **Body mass index**  **(kg/m^2^)** |  |  |  |  |  |
| Mean (SD) | 30.6±6.1 | | 30.9±6.4 | | P = 0.004 |
| <25 | 1313 | (14.8%) | 936 | (14.1%) |  |
| ≥25, <30 | 3230 | (36.5%) | 2299 | (34.7%) |  |
| ≥30<35 | 2429 | (27.5%) | 1811 | (27.3%) |  |
| ≥35 | 1619 | (18.3%) | 1342 | (20.2%) | P = 0.007 |
| Unknown | 255 | (2.9%) | 246 | (3.7%) |  |
|  |  |  |  |  |  |
| **Cigarette smoking** |  |  |  |  |  |
| Current | 555 | (6.3%) | 724 | (10.9%) |  |
| Former | 3947 | (44.6%) | 3104 | (46.8%) |  |
| Never | 4243 | (48.0%) | 2734 | (41.2%) | P<0.001 |
| Unknown | 101 | (1.1%) | 72 | (1.1%) |  |
|  |  |  |  |  |  |
|  |  |  |  |  |  |
| **Non-study medication** |  |  |  |  |  |
| ACE inhibitor or ARB | 5115 | (57.8%) | 3940 | (59.4%) | P = 0.051 |
| Aspirin use before screening | 3157 | (35.7%) | 2351 | (35.4%) | P = 0.76 |
| Thiazide or related diuretic | 1639 | (18.5%) | 1318 | (19.9%) | P<0.001 |
| Calcium channel blocker | 2088 | (23.6%) | 1685 | (25.4%) | P = 0.01 |
| Statin | 6769 | (76.5%) | 4884 | (73.6%) | P<0.001 |
|  |  |  |  |  |  |
| **Total cholesterol**  **(mmol/L)** |  |  |  |  |  |
| Mean (SD) | 4.1±0.9 | | 4.2±0.9 | | P = 0.07 |
| <4 | 2681 | (30.3%) | 1873 | (28.2%) |  |
| ≥4 <5 | 2246 | (25.4%) | 1549 | (23.4%) |  |
| ≥5 | 817 | (9.2%) | 653 | (9.8%) | P = 0.046 |
| Not available | 3102 | (35.1%) | 2559 | (38.6%) |  |
|  |  |  |  |  |  |
| **HDL cholesterol**  **(mmol/L)** |  |  |  |  |  |
| Mean (SD) | 1.3±0.4 | | 1.3±0.4 | | P<0.001 |
| <1 | 1222 | (13.8%) | 948 | (14.3%) |  |
| ≥1<1.5 | 3202 | (36.2%) | 2321 | (35.0%) |  |
| ≥1.5 | 1310 | (14.8%) | 797 | (12.0%) | P<0.001 |
| Not available | 3112 | (35.2%) | 2568 | (38.7%) |  |
|  |  |  |  |  |  |
| **Non-HDL cholesterol**  **(mmol/L)** |  |  |  |  |  |
| Mean (SD) | 2.9±0.8 | | 2.9±0.9 | | P<0.001 |
| <2.5 | 2053 | (23.2%) | 1337 | (20.2%) |  |
| ≥2.5 <3.5 | 2558 | (28.9%) | 1835 | (27.7%) |  |
| ≥3.5 | 1123 | (12.7%) | 894 | (13.5%) | P = 0.002 |
| Not available | 3112 | (35.2%) | 2568 | (38.7%) |  |
|  |  |  |  |  |  |
| **Glycosylated haemoglobin - HbA1c** |  |  |  |  |  |
| IFCC (mmol/mol) mean (SD) | 54.0±12.2 | | 55.9±13.8 | | P<0.001 |
| DCCT (%) mean (SD) | 7.1±1.1 | | 7.3±1.3 | | P<0.001 |
| <48 (6.5) | 1957 | (22.1%) | 1315 | (19.8%) |  |
| ≥48 (6.5), <64 (8.0) | 2792 | (31.6%) | 1872 | (28.2%) |  |
| ≥64 (8.0) | 990 | (11.2%) | 887 | (13.4%) | P<0.001 |
| Not available | 3107 | (35.1%) | 2560 | (38.6%) |  |
|  |  |  |  |  |  |
| **CKD-EPI estimated GFR**  **(ml/min/1.73m^2^)** |  |  |  |  |  |
| Mean (SD) | 87.8±19.8 | | 81.5±22.3 | | P<0.001 |
| ≥90 | 2902 | (32.8%) | 1621 | (24.4%) |  |
| ≥60<90 | 2299 | (26.0%) | 1717 | (25.9%) |  |
| <60 | 540 | (6.1%) | 736 | (11.1%) | P<0.001 |
| Not available | 3105 | (35.1%) | 2560 | (38.6%) |  |
|  |  |  |  |  |  |
| **Urinary albumin:creatinine ratio**  **(mg/mmol)** ‡ |  |  |  |  |  |
| Median (IQR) | 0.50 (0.00-1.14) | | 0.66 (0.24-1.66) | | P<0.001 |
| <3 | 5120 | (57.9%) | 3406 | (51.3%) |  |
| ≥3 | 613 | (6.9%) | 635 | (9.6%) | P<0.001 |
| Not available | 3113 | (35.2%) | 2593 | (39.1%) |  |
|  |  |  |  |  |  |
| **Townsend Deprivation Index** |  |  |  |  |  |
| <-3 | 3101 | (35.1%) | 2003 | (30.2%) |  |
| ≥-3<0 | 3588 | (40.6%) | 2434 | (36.7%) |  |
| ≥0<2 | 1071 | (12.1%) | 966 | (14.6%) |  |
| ≥2<4 | 625 | (7.1%) | 690 | (10.4%) |  |
| ≥4<6 | 323 | (3.7%) | 380 | (5.7%) |  |
| ≥6 | 118 | (1.3%) | 143 | (2.2%) | P<0.001 |
| Unknown | 20 | (0.2%) | 18 | (0.3%) |  |
|  |  |  |  |  |  |
| **Ethnic origin** |  |  |  |  |  |
| White | 8564 | (96.8%) | 6371 | (96.0%) |  |
| Indian/Pakistani/Bangladeshi | 107 | (1.2%) | 77 | (1.2%) |  |
| African/Caribbean | 54 | (0.6%) | 86 | (1.3%) |  |
| Other/unknown | 121 | (1.4%) | 100 | (1.5%) | P<0.001 |

DCCT = Diabetes Control and Complications Trial; FAs = Fatty acids; GFR = Glomerular Filtration Rate; HDL=High-density lipoprotein; IFCC =International Federation of Clinical Chemistry; IQR = Interquartile range; SD = Standard Deviation

Figures presented are counts with percentages unless otherwise stated.

*Formal testing for differences in baseline characteristics between those included in and excluded from the analysis cohort used the Chi-squared test for heterogeneity for categorical variables and grouped continuous variables (including age, duration of diabetes, systolic blood pressure, diastolic blood pressure, body mass index, total cholesterol, HDL-cholesterol, non-HDL cholesterol, HbA1c, eGFR and urinary albumin:creatinine ratio), the Mantel-Haenszel Chi-square test for trend for ordinal variables (i.e. Townsend Deprivation Index), and the t-test or Wilcoxon-Mann-Whitney U test for continuous variables with a normal (summarised by mean±SD) or non-normal (summarised by median and interquartile range) distribution respectively. Categories that were “unknown” or “not available” were excluded.

Percentages may not total 100 because of rounding

1. Sight-threating eye bleeding was included in the secondary endpoint of “Major haemorrhage” in ASCEND [↑](#footnote-ref-1)
2. In this section of the questionnaire, participants could record information about any other serious eye conditions via freetext, which was later coded by a clinician at the coordinating centre [↑](#footnote-ref-2)
3. If events are identified from more than one source, they will be represented in every subgroup, however, the overall incidence will be based on the earliest occurrence across all three data sources. [↑](#footnote-ref-3)
4. In blinded preliminary analyses, only a small number of individuals had the highest retinopathy grades, and therefore the following combinations were grouped into the ≥R_2_/≥R_0_ category: ≥R_2_/R_0_ or R_0_/≥R_2_, ≥R_2_/R_1_ or R_1_/≥R_2_ and ≥R_2_/≥R_2_ [↑](#footnote-ref-4)
5. Before the linkage data were received, it was conceivable for individual DESPs to use one of several standardised charts to measure visual acuity. To facilitate analyses of visual acuity derived from different charts, grades were converted into a new code called the “ASCEND-Eye Recode Score”. Appendix 4 is the table that was used for this purpose; each row represents a complete line of letters of equivalent size on each chart. Incremental scores between rows indicated that a participant was able to read some, but not all of the smaller letters on the next row. In such cases, scores were rounded down to the nearest complete line. [↑](#footnote-ref-5)
6. An increase of 3 or more in the ASCEND-Eye Recode scoring system, corresponds to a loss of 15 or more ETDRS letters, which was recommended by the US Food and Drug Administration, as a clinically-relevant outcome measure in ophthalmology trials. A 15 letter change represents a doubling of the visual angle (or a doubling of the size of letters on a standard ETDRS chart). [↑](#footnote-ref-6)
